# Growth Hormone Mediators and Glycemic Control in Youths With Type 2 Diabetes: A Secondary Analysis of a Randomized Clinical Trial

**DOI:** 10.1001/jamanetworkopen.2024.0447

**Published:** 2024-02-29

**Authors:** Chang Lu, Danielle Wolfs, Laure El ghormli, Lynne L. Levitsky, Lorraine E. Levitt Katz, Lori M. Laffel, Mary-Elizabeth Patti, Elvira Isganaitis

**Affiliations:** 1 Division of Endocrinology, Boston Children’s Hospital, Harvard Medical School, Boston, Massachusetts; 2 Joslin Diabetes Center, Harvard Medical School, Boston, Massachusetts; 3 The Biostatistics Center, George Washington University, Washington, DC; 4 Division of Pediatric Endocrinology and Diabetes, Massachusetts General Hospital, Harvard Medical School, Boston; 5 Division of Endocrinology and Diabetes, Children’s Hospital of Philadelphia, The Perelman School of Medicine at the University of Pennsylvania, Philadelphia

## Abstract

**Importance:**

Youth-onset type 2 diabetes (T2D) has a more aggressive phenotype than adult-onset T2D, including rapid loss of glycemic control and increased complication risk.

**Objective:**

To identify associations of growth hormone mediators with glycemic failure, beta cell function, and insulin sensitivity in youth-onset T2D.

**Design, Setting, and Participants:**

This post hoc secondary analysis of the Treatment Options for Type 2 Diabetes in Adolescents and Youth (TODAY) randomized clinical trial, which enrolled participants from July 2004 to February 2009, included 398 participants from 15 university-affiliated medical centers with available plasma samples from baseline and 36 months. Participants were youths aged 10 to 17 years with a duration of T2D of less than 2 years who were randomized to metformin, metformin plus lifestyle intervention, or metformin plus rosiglitazone. Participants were followed up for a mean (SD) of 3.9 (1.5) years during the trial, ending in 2011. Statistical analysis was performed from August 2022 to November 2023.

**Exposure:**

Plasma insulin-like growth factor-1 (IGF-1), growth hormone receptor (GHR), and insulin-like growth factor binding protein 1 (IGFBP-1).

**Main Outcomes and Measures:**

Main outcomes were (1) loss of glycemic control during the TODAY study, defined as hemoglobin A_1c_ (HbA_1c_) level of 8% or more for 6 months or inability to wean from insulin therapy, and (2) baseline and 36-month measures of glycemia (fasting glucose, HbA_1c_), insulin sensitivity (1/fasting C-peptide), high-molecular-weight adiponectin, and beta cell function (C-peptide index, C-peptide oral disposition index).

**Results:**

This analysis included 398 participants (mean [SD] age, 13.9 [2.0] years; 248 girls [62%]; 166 Hispanic participants [42%]; 134 non-Hispanic Black participants [34%], and 84 non-Hispanic White participants [21%]). A greater increase in IGF-1 level between baseline and 36 months was associated with lower odds of glycemic failure (odds ratio [OR], 0.995 [95% CI, 0.991-0.997]; *P* < .001) and higher C-peptide index per 100-ng/mL increase in IGF-1 (β [SE], 0.015 [0.003]; *P* < .001). A greater increase in log_2_ GHR level between baseline and 36 months was associated with higher odds of glycemic failure (OR, 1.75 [95% CI, 1.05-2.99]; *P* = .04) and lower C-peptide index (β [SE], −0.02 [0.006]; *P* < .001). A greater increase in log_2_ IGFBP-1 level between baseline and 36 months was associated with higher odds of glycemic failure (OR, 1.37 [95% CI, 1.09-1.74]; *P* = .007) and higher high-molecular-weight adiponectin (β [SE], 431 [156]; *P* = .007).

**Conclusions and Relevance:**

This study suggests that changes in plasma growth hormone mediators are associated with loss of glycemic control in youth-onset T2D, with IGF-1 associated with lower risk and GHR and IGFBP-1 associated with increased risk.

**Trial Registration:**

ClinicalTrials.gov Identifier: NCT00081328

## Key Points

**Question:**

Are changes in mediators of growth hormone signaling during puberty associated with characteristics of youth-onset type 2 diabetes (T2D)?

**Findings:**

In this post hoc secondary analysis of a randomized clinical trial of 398 participants with youth-onset T2D, changes from baseline to 3 years in plasma concentrations of growth hormone mediators, including insulin-like growth factor-1, growth hormone receptor, and insulin-like growth factor binding protein 1, were associated with glycemic failure and measures of insulin resistance and beta cell function.

**Meaning:**

These data suggest that growth hormone mediators may be associated with the unique phenotype of pediatric T2D.

## Teaser

This secondary analysis of a randomized clinical trial examines associations of growth hormone mediators with glycemic failure, beta cell function, and insulin sensitivity in youth-onset type 2 diabetes.

## Introduction

Type 2 diabetes (T2D), once considered a disease of aging, has reached unprecedented levels among children and adolescents and disproportionately affects Black, Hispanic, and socioeconomically disadvantaged youths.^1^ Compared with adults with T2D, children with T2D have more severe insulin resistance and accelerated beta cell failure, yet therapies for children are limited and less effective.^2,3^ Treatment Options for Type 2 Diabetes in Adolescents and Youth (TODAY) was a multicenter, longitudinal study of youth-onset T2D.^4^ In the primary randomized clinical trial phase of the study (N = 699), nearly half the participants reached the primary outcome of glycemic failure within 4 years.^5^ In the observational phase of the study (termed *TODAY2*), 60% of participants developed complications in the 10-year follow-up period, highlighting the aggressive natural history of youth-onset T2D.^6^

The mechanisms underlying rapidly worsening glycemia and beta cell function in youth-onset T2D remain unclear. Among children, T2D occurs almost exclusively during or after puberty, suggesting that hormonal dynamics may play a role in the pathogenesis and/or progression.^7,8,9^ Puberty is marked by activation of sex hormones and peak secretion of growth hormone (GH) and its effectors.^10^ Growth hormone signaling may have complex effects on carbohydrate metabolism through several mediators, including insulin-like growth factor-1 (IGF-1), growth hormone receptor (GHR), and IGF binding proteins (IGFBPs).^11,12,13^ These GH mediators may directly or indirectly affect body composition, insulin sensitivity, and insulin secretion, potentially influencing the emergence of T2D and its natural history.

Thus, our objective was to study associations of GH mediators with measures of glycemic control, insulin sensitivity, and beta cell function, using samples from the TODAY study cohort at baseline and at 36 months—a time point at which most of the cohort had completed puberty and approximately half the participants had a loss of glycemic control.^5^ We hypothesized that baseline concentrations of, and changes in, IGF-1, GHR, and IGFBP-1 from baseline to 36 months may be associated with the primary outcome of glycemic failure in the TODAY trial. Secondarily, we hypothesized that plasma IGF-1, GHR, and IGFBP-1 concentrations would be associated with measures of glycemia, insulin sensitivity, and beta cell function.

## Methods

### TODAY Study Design and Cohort

The TODAY study design, methods, and results have been previously reported (study protocol in Supplement 1).^5^ In brief, TODAY was a randomized clinical trial in which 699 youths were enrolled from 15 US clinical centers between July 2004 and February 2009, based on inclusion criteria of age 10 to 17 years, T2D duration less than 2 years, body mass index (BMI; calculated as weight in kilograms divided by height in meters squared) in the 85th percentile or higher, negative pancreatic autoantibodies, and plasma C-peptide level greater than 0.6 ng/mL (to convert to nanomoles per liter, multiply by 0.331) (eFigure 1 in Supplement 2). Participants were randomized to metformin monotherapy, metformin plus lifestyle intervention, or metformin plus rosiglitazone. Participants were followed up for 2 to 6.5 years (mean [SD] follow-up, 3.9 [1.5] years) within the initial TODAY trial; the primary outcome was time to glycemic failure, defined as hemoglobin A_1c_ (HbA_1c_) level of 8% or more (to convert to proportion of total hemoglobin, multiply by 0.01; to convert to mmol/mol, multiply by 10.93 and subtract 23.5) for 6 months, or acute metabolic decompensation requiring insulin. Participants were then transitioned from study medications to diabetes care in the community. Physical examinations and anthropometric measurements were obtained throughout the study. Tanner stage was based on physical examination of breasts and pubic hair for girls and testicular volume and pubic hair for boys and dichotomized to Tanner stage 1, 2, and 3 vs Tanner stage 4 and 5. The following post hoc analyses used all remaining stored plasma samples from the baseline and 36-month time points (n = 398). Time-to-event survival analysis of this substudy cohort demonstrated similar rates of glycemic failure as reported in the full cohort (N = 699) (eFigure 2 in Supplement 2).^5^ Of the subset, 310 participants had available samples at both baseline and 36 months. To assess potential effects of missing data, we compared the subgroup of 310 individuals with paired samples vs the 88 individuals with only baseline samples (eTables 2 and 4 in Supplement 2) and performed sensitivity analyses with the full subcohort (n = 398) vs those with paired samples (n = 310). All 15 TODAY study centers (Baylor College of Medicine, Case Western Reserve University, Children's Hospital Los Angeles, Children's Hospital of Philadelphia, Children's Hospital of Pittsburgh, Columbia University Medical Center, Joslin Diabetes Center, Massachusetts General Hospital, Saint Louis University, State University of New York Upstate Medical University, University of Colorado Denver, University of Oklahoma Health Sciences Center, University of Texas Health Science Center at San Antonio, Washington University in St Louis, and Yale University) obtained institutional review board approval and written informed assent and/or consent from participants and parents or guardians. This report followed the Consolidated Standards of Reporting Trials (CONSORT) reporting guideline for randomized studies.

### Measures of Glycemia, Insulin Sensitivity, and Insulin Secretion

Assessments of glycemia in TODAY included HbA_1c_ level and a 2-hour oral glucose tolerance test (OGTT) every 6 months during the first year and annually thereafter, at the time of the primary outcome, and at study end. Plasma glucose and C-peptide measurements during the OGTT were used to calculate insulin secretion and sensitivity indices. Insulin sensitivity was assessed as 1/fasting C-peptide.^14,15^ Given that fasting C-peptide may be affected by beta cell function, we used high-molecular-weight adiponectin (HMWA) as an additional insulin sensitivity measure.^14,16^ C-peptide index, a measure of beta cell function, is calculated as a change in C-peptide from 0 to 30 minutes/change in glucose from 0 to 30 minutes during the OGTT.^17^ The C-peptide oral disposition index (CODI), a measure of beta cell insulin secretion in response to insulin resistance, is calculated as the product of 1/fasting insulin × C-peptide index.^17^ Wherever possible, we used C-peptide–based indices rather than insulin-based indices to avoid potential confounding by exogenous insulin.

### IGF-1, GHR, and IGFBP-1 Assays

We measured IGF-1 using a commercial enzyme-linked immunosorbent assay (ELISA) kit (No. 22-IGFHU-E01; ALPCO) according to manufacturer instructions. Study samples were run once, and standard curves were run in duplicate on 96-well plates. The standards for IGF-1 were fitted to a trinomial curve using a BioTek Gen5 Microplate Reader and Imager Software (No. BTGENSCPRIM; Agilent Technologies Inc). We similarly measured GHR levels using a commercial ELISA kit (No. ab260060; Abcam plc); standards for GHR were fitted to a 4-parameter curve. For IGF-1 and GHR, concentrations were determined by interpolating blank-subtracted absorbances against the standard curve, then multiplying by the dilution factor. Intra-assay and interassay coefficients of variation for control samples were 2.2% and 9.1%, respectively, for IGF-1 and 6.5% and 14.0%, respectively, for GHR. To account for higher inter-assay coefficients of variation, each IGF-1 and GHR sample concentration was scaled to a control sample run in duplicate on all plates. Assays for IGF-1 and GHR were performed between March and August 2022. Insulin-like growth factor binding protein 1 was measured using a magnetic bead-based multiplex protein array (R&D Systems), as reported previously.^18^ For IGFBP-1, intra-assay and interassay coefficients of variation were 4.6% to 5.5% and 7.1% to 10.8%, respectively.

### Statistical Analysis

Statistical analysis was performed from August 2022 to November 2023. For between-group comparisons of participants with glycemic failure vs glycemic control, we used the *t* test for continuous variables and the χ^2^ test for categorical variables. We used a paired *t* test when comparing baseline and 36-month values for the same participant. Growth hormone receptor and IGFBP-1 concentrations were log_2_ transformed because the data were not normally distributed. Logged concentrations of GHR and IGFBP-1 were used in all analyses. We performed multivariable logistic regression with covariate adjustment to assess associations of baseline levels or a 36-month change in levels of each hormone (IGF-1, log_2_ GHR, and log_2_ IGFBP-1) with the odds of glycemic failure. All logistic regression models included sex, age, race and ethnicity, BMI *z* score, treatment group, baseline HbA_1c_ level, and Tanner stage; 36-month models additionally adjusted for baseline hormone level. The participant and parent or another knowledgeable adult reported race and ethnicity to a study interviewer. The race and ethnicity categories were determined based on National Institutes of Health reporting guidelines. Race and ethnicity were recorded to corroborate that the demographic characteristics of our cohort are representative of the epidemiology of T2D in youths. In addition, reporting of race and ethnicity allowed us to include these as covariates in adjusted models. We used multivariable linear regression to assess the association of baseline levels or a 36-month change in levels of each hormone with metabolic measures, including HbA_1c_ level, fasting glucose, 1/fasting C-peptide, HMWA, C-peptide index, and CODI. All linear models adjusted for sex, age, race and ethnicity, BMI *z* score, and Tanner stage, and 36-month models additionally adjusted for treatment group, baseline glycemic measure, and baseline hormone levels. In reporting analyses including IGF-1, a ×100 multiplier was applied to the β coefficients and SEs to facilitate interpretation of the results. *P* < .05 was considered statistically significant. Statistical analyses were performed using R, version 2023.03.0+386 (R Project for Statistical Computing) and Prism, version 9.5.0 (GraphPad).

## Results

### Demographic Characteristics

A subset of 398 participants (mean [SD] age, 13.9 [2.0] years; 248 girls [62%]; 166 Hispanic participants [42%]; 134 non-Hispanic Black participants [34%], and 84 non-Hispanic White participants [21%]) from the TODAY study were included in this ancillary analysis (eTable 1 in Supplement 2). Of these, 310 participants had both baseline and 36-month samples, and 88 participants had only baseline samples available for analysis. A total of 182 participants (46%) reached the primary outcome of glycemic failure, whereas 216 participants (54%) maintained glycemic control. By 36 months, 164 of 182 participants (90%) in the glycemic failure group had reached the primary outcome (mean [SD] time to primary outcome, 15.2 [9.8] months); 18 of 182 participants (10%) in this group had a loss of glycemic control by the end of the 5-year randomized clinical trial phase of the TODAY study. At baseline, participants had a mean (SD) BMI *z* score of 2.2 (0.5) and 353 of 392 participants (90%) had a Tanner stage of 4 or 5. Compared with the glycemic control group, the glycemic failure group included more Hispanic and non-Hispanic Black participants (Hispanic, 45% [82 of 182] vs 39% [84 of 216]; non-Hispanic Black, 37% [68 of 1820] vs 31% [66 of 216]; *P* = .02) and had a longer T2D duration (mean [SD] duration, 8.2 [6.0] months vs 7.0 [5.4] months; *P* = .04). Subgroup analysis comparing participants with samples at both time points (n = 310) with those with only baseline samples (n = 88) showed that the 2 subgroups were similar except for slightly older age and lower height among those with baseline samples only (eTable 2 in Supplement 2). Given these differences, we performed sensitivity analyses in which we repeated all analyses for the 310 participants with paired samples; we found similar associations of GH mediators with glycemic measures, with any differences noted.

### Measures of Glycemia, Insulin Sensitivity, and Beta Cell Function

Glycemic measures in the TODAY study have been reported previously.^17^ The mean (SD) HbA_1c_ level was 6.1% (0.8%) at baseline and 7.7% (2.6%) at 36 months. Compared with the glycemic control group at baseline, the glycemic failure group had higher HbA_1c_ levels, fasting glucose levels, and 2-hour OGTT glucose values and lower C-peptide levels, C-peptide indexes, and CODI values. At 36 months, all measures of glycemia, insulin sensitivity, and beta cell function were worse in the glycemic failure group compared with the glycemic control group (eTable 3 in Supplement 2).

### GH Mediators and Loss of Glycemic Control

We tested whether GH mediators changed between baseline and 36 months and whether they differed based on glycemic outcome. Levels of GH mediators did not differ based on TODAY treatment group assignment but did differ between the glycemic control and glycemic failure groups. Compared with the glycemic control group, the glycemic failure group had lower plasma IGF-1 levels at baseline (mean [SD] level, 367 [141] ng/mL vs 396 [132] ng/mL; *P* = .03) and at 36 months (mean [SD] level, 282 [106] ng/mL vs 332 [124] ng/mL; *P* < .001) (Figure, A). The mean IGF-1 level was lower at 36 months than at baseline in both the glycemic failure group (mean [SD] level, 282 [106] ng/mL vs 367 [141] ng/mL; *P* < .001) and glycemic control group (mean [SD] level, 332 [124] ng/mL vs 396 [132] ng/mL; *P* < .001). The mean plasma log_2_ GHR level was higher in the glycemic failure group than in the glycemic control group at 36 months only (mean [SD] level, 5.5 [0.6] ng/mL vs 5.3 [0.6] ng/mL; *P* = .03) and did not differ between baseline and 36 months for either group (Figure, B). The mean plasma log_2_ IGFBP-1 levels increased from baseline to 36 months within each outcome group (mean [SD] level, 1.6 [1.5] ng/mL vs 2.3 [1.7] ng/L in the glycemic failure group; *P* < .001; mean [SD] level, 1.4 [1.2] vs 1.8 [1.6] ng/mL in the glycemic control group; *P* < .001), but they were higher in the glycemic failure group compared with the glycemic control group at 36 months only (mean [SD] level, 2.3 [1.7] ng/L vs 1.8 [1.6] ng/mL; *P* = .009) (Figure, C).

**Figure.**
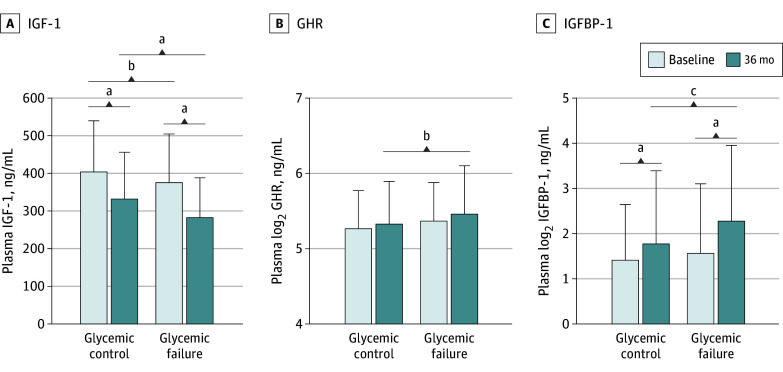
Growth Hormone Mediator Levels at Baseline and 36-Month Visits Based on Outcome in the Treatment Options for Type 2 Diabetes in Adolescents and Youth Study Plasma concentrations of insulin-like growth factor-1 (IGF-1) (n = 310), growth hormone receptor (GHR) (n = 310), and insulin-like growth factor binding protein 1 (IGFBP-1) (n = 286) between trial outcome groups that lost glycemic control vs maintained glycemic control at baseline and 36 months. *P* values refer to the 2-tailed paired *t* test (for baseline vs 36-month comparisons) and the unpaired *t* test (for glycemic failure vs glycemic control groups). ^a^*P* < .001. ^b^*P* < .05. ^c^*P* < .01.

We next used logistic regression to determine whether GH mediator concentrations were associated with the odds of glycemic failure during the TODAY trial, adjusting for sex, age, race and ethnicity, BMI *z* score, treatment group, baseline HbA_1c_ level, and Tanner stage in all models and additionally for baseline hormone level in the 36-month model. Baseline IGF-1, log_2_ GHR, and log_2_ IGFBP-1 levels were not associated with odds of glycemic failure. By contrast, changes in GH mediators between baseline and 36 months were significantly associated with odds of glycemic failure. An increase in IGF-1 level from baseline to 36 months was associated with lower odds of glycemic failure (odds ratio [OR], 0.995 [95% CI, 0.991-0.997]; *P* < .001; Table 1) and higher C-peptide index per 100-ng/mL increase in IGF-1 level (β [SE], 0.015 [0.003]; *P* < .001; Table 2). An increase in log_2_ GHR level from baseline to 36 months was associated with higher odds of glycemic failure (OR, 1.75 [95% CI, 1.05-2.99]; *P* = .04; Table 1) and lower C-peptide index (β [SE], −0.02 [0.006]; *P* < .001; Table 3). Similarly, an increase in log_2_ IGFBP-1 level between baseline and 36 months was associated with higher odds of glycemic failure (OR, 1.37 [95% CI, 1.09-1.74]; *P* = .007; Table 1) and higher high-molecular-weight adiponectin (β [SE], 431 [156]; *P* = .007; Table 4).

**Table 1.** Associations of Plasma Hormone Levels With Odds of Primary Outcome in the Treatment Options for Type 2 Diabetes in Adolescents and Youth Study

Multivariable model	Odds ratio (95% CI)	*P* value^a^
IGF-1 level		
Baseline^b^	0.999 (0.997-1.000)	.18
Change from baseline to 36 mo^c^	0.995 (0.991-0.997)	<.001
Log^2^ GHR level		
Baseline^b^	1.17 (0.68-2.01)	.57
Change from baseline to 36 mo^c^	1.75 (1.05-2.99)	.04
Log_2_ IGFBP-1 level		
Baseline^b^	1.07 (0.90-1.29)	.44
Change from baseline to 36 mo^c^	1.37 (1.09-1.74)	.007

Abbreviations: GHR, growth hormone receptor; IGF-1, insulin-like growth factor-1; IGFBP-1, insulin-like growth factor binding protein 1.

^a^ From logistic regression models with covariate adjustments.

^b^ Adjusted for sex, age, race, body mass index *z* score, treatment group, baseline hemoglobin A_1c_, and Tanner stage.

^c^ Adjusted for sex, age, race, body mass index *z* score, treatment group, baseline hemoglobin A_1c_, Tanner stage, and baseline hormone level.

**Table 2.** Associations of Plasma IGF-1 Levels With Glycemic Measures at Baseline and 36 Months^a^

Glycemic measure	Baseline IGF-1 vs baseline glycemic measures^b^		Change in IGF-1 vs 36-mo glycemic measures^c^	
	β (SE) × 100	*P* value	β (SE) × 100	*P* value
Hemoglobin A_1c_, %	−0.08 (0.03)	.01	−0.66 (0.14)	<.001
Fasting glucose, mg/dL	−2.0 (1.0)	.06	−20.0 (4.0)	<.001
1/Fasting C-peptide, mL/ng	−0.008 (0.005)	.14	−0.08 (0.09)	.40
HMWA, ng/mL	58 (76)	.44	620 (212)	.004
C-peptide index, ng/mL per mg/dL	0.005 (0.003)	.12	0.015 (0.003)	<.001
CODI, mL/uU × ng/mL per mg/dL	0.0002 (0.0002)	.32	0.0008 (0.0002)	<.001

Abbreviations: CODI, C-peptide oral disposition index; HMWA, high-molecular-weight adiponectin; IGF-1, insulin-like growth factor-1.

^a^ Data are β coefficients and SE from linear regression models, multiplied by 100, with associated *P* values.

^b^ Adjusted for sex, age, race, baseline body mass index *z* score, and Tanner stage.

^c^ Adjusted for sex, age, race, baseline body mass index *z* score, Tanner stage, treatment group, baseline glycemic measure, and baseline IGF-1.

**Table 3.** Associations of Plasma Log_2_ GHR Levels With Glycemic Measures at Baseline and 36 Months^a^

Glycemic measure	Baseline log_2_ GHR vs baseline glycemic measures^b^		Change in log_2_ GHR vs 36-mo glycemic measures^c^	
	β (SE)	*P* value	β (SE)	*P* value
Hemoglobin A_1c_, %	0.36 (0.09)	<.001	0.96 (0.26)	<.001
Fasting glucose, mg/dL	11.4 (2.7)	<.001	43.4 (7.9)	<.001
1/Fasting C-peptide, mL/ng	−0.09 (−0.01)	<.001	−0.02 (0.17)	.93
HMWA, ng/mL	−735 (204)	<.001	−1340 (390)	<.001
C-peptide index, ng/mL per mg/dL	−0.01 (0.008)	.19	−0.02 (0.006)	<.001
CODI, mL/uU × ng/mL per mg/dL	−0.001 (0.0005)	.01	−0.002 (0.0003)	<.001

Abbreviations: CODI, C-peptide oral disposition index; GHR, growth hormone receptor; HMWA, high-molecular-weight adiponectin.

^a^ Data are β coefficients, SE, and *P* values from linear regression models with covariate adjustments.

^b^ Adjusted for sex, age, race, baseline body mass index *z* score, and Tanner stage.

^c^ Adjusted for sex, age, race, baseline body mass index *z* score, Tanner stage, treatment group, baseline glycemic measure, and baseline GHR.

**Table 4.** Associations of Plasma Log_2_ IGFBP-1 Levels With Glycemic Measures at Baseline and 36 Months^a^

Glycemic measure	Baseline log_2_ IGFBP-1 vs baseline glycemic measures^b^		Change in log_2_ IGFBP-1 vs 36-mo glycemic measures^c^	
	β (SE)	*P* value	β (SE)	*P* value
Hemoglobin A_1c_, %	0.02 (0.03)	.50	0.43 (0.11)	<.001
Fasting glucose, mg/dL	1.6 (1.0)	.10	19.6 (3.3)	<.001
1/Fasting C-peptide, mL/ng	0.04 (0.005)	<.001	−0.01 (0.07)	.88
HMWA, ng/mL	150 (71)	<.001	431 (156)	.007
C-peptide index, ng/mL per mg/dL	−0.002 (0.003)	.40	−0.007 (0.003)	.15
CODI, mL/uU × ng/mL per mg/dL	0.0001 (0.0002)	.55	0.0001 (0.0002)	.76

Abbreviations: CODI, C-peptide oral disposition index; HMWA, high-molecular-weight adiponectin; IGFBP-1, insulin-like growth factor binding protein 1.

^a^ Data are β coefficients, SE, and *P* values from linear regression models with covariate adjustments.

^b^ Adjusted for sex, age, race, baseline body mass index *z* score, and Tanner stage.

^c^ Adjusted for sex, age, race, baseline body mass index *z* score, Tanner stage, treatment group, baseline glycemic measure, and baseline IGFBP-1.

### GH Mediators and Glycemic Measures

#### Insulin-Like Growth Factor-1

We next used linear regression to model the associations of GH mediators with glycemic measures at baseline and 36 months, adjusting for sex, age, race and ethnicity, BMI *z* score, and Tanner stage. In addition, the 36-month models adjusted for treatment group, baseline glycemic measure, and baseline hormone levels. The β coefficients and SEs were multiplied by 100 to reflect changes in glycemic measures per every 100-ng/mL change in IGF-1 concentration. At baseline, a higher IGF-1 level was associated with a lower HbA_1c_ level (Table 2). A greater increase in IGF-1 level from baseline to 36 months was associated with lower HbA_1c_ and fasting glucose levels and higher HMWA, C-peptide index, and CODI at 36 months (Table 2).

#### Growth Hormone Receptor

At baseline, higher log_2_ GHR concentrations were associated with higher HbA_1c_ and fasting glucose levels and lower 1/fasting C-peptide, HMWA, and CODI (Table 3). A greater increase in log_2_ GHR level from baseline to 36 months was associated with higher HbA_1c_ and fasting glucose levels and lower HMWA, C-peptide index, and CODI at 36 months.

#### Insulin-Like Growth Factor Binding Protein 1

At baseline, a higher log_2_ IGFBP-1 level was associated with higher 1/fasting C-peptide and HMWA (Table 4). A greater increase in log_2_ IGFBP-1 level between baseline and 36 months was associated with higher HbA_1c_ level, fasting glucose level, and HMWA at 36 months. In the sensitivity analysis restricted to the 310 participants with paired samples, the association of baseline log_2_ IGFBP-1 level with HMWA was no longer statistically significant; however, the direction of the association remained the same.

### Correlations of IGF-1, GHR, and IGFBP-1 Levels

We explored the association between pairs of GH mediators using a Pearson correlation of hormone concentrations at baseline (eFigure 3 in Supplement 2). The log_2_ GHR level was inversely correlated with the IGF-1 level (*r* = −0.33; *P* < .001) and the log_2_ IGFBP-1 level (*r* = −0.19; *P* < .001), and the IGF-1 level was inversely correlated with the log_2_ IGFBP-1 level (*r* = −0.13; *P* = .02).

## Discussion

In children, T2D usually emerges during or after puberty, a period of rapid growth. Youth-onset T2D is typically more aggressive than adult-onset T2D, yet no prior studies have tested whether GH or its mediators are associated with glycemic outcomes in youth-onset T2D. Using the TODAY study, one of the largest studies on treatment and natural history of pediatric T2D to our knowledge, we chose to study 3 GH mediators: (1) IGF-1, the primary mediator of GH action; (2) GHR, a surrogate of GH bioavailability and action; and (3) IGFBP-1, a regulator of IGF-1 bioavailability and potential mediator of insulin sensitivity. We identified significant associations between these GH mediators and glycemic outcomes in youths with T2D.

In our analysis, youths with a loss of glycemic control in the TODAY study had lower IGF-1 levels, higher IGFBP-1 levels, and higher GHR levels at 36 months than participants who maintained glycemic control. A greater increase in IGF-1 level between baseline and 36 months was associated with lower odds of glycemic failure, while greater increases in IGFBP-1 and GHR levels were associated with increased odds of glycemic failure. In addition, we found significant correlations among the 3 hormones, which may reflect a direct association between glucose metabolism and GH signaling or a common upstream mechanism causing changes in both processes, such as puberty, obesity, or hepatic insulin resistance. Future studies of the combined signaling effects of GH mediators on glucose metabolism will be important to elucidate the mechanisms underlying these associations.

A greater increase in IGF-1 level between baseline and 36 months was associated with a lower HbA_1c_ level and higher measures of beta cell function (insulinogenic index, oral disposition index). Although we are not aware of any prior analyses of IGF-1 levels in youth-onset T2D, lower IGF-1 levels have been reported previously in youths with obesity (compared with youths with normal weight) and in adults with obesity and diabetes.^19,20^ However, whether a lower IGF-1 level is the cause or result of obesity and diabetes is unclear. In adults with T2D, plasma IGF-1 and C-peptide levels have been reported to be positively correlated.^21^ In a cohort of low-birth-weight children without diabetes, a higher IGF-1 level at 5 years was associated with higher insulin secretion (insulinogenic index) at 8 years.^22^ Together, these data suggest a potential role of IGF-1 in preserving beta cell function. Consistent with this possibility, rodent studies have shown that IGF-1 overexpression in beta cells increases beta cell mass through neogenesis and replication.^23^ Insulin-like growth factor-1 may also enhance insulin action based on homology to insulin and binding to the insulin receptor,^24^ and administration of recombinant human IGF-1 in adults with obesity improves glycemia and insulin sensitivity.^25,26^ Whether modulating the IGF-1 pathway in youths with T2D may be beneficial remains an important question with implications for future therapies.

We found that a higher baseline GHR level and a greater increase in GHR level between baseline and 36 months were positively associated with hyperglycemia, insulin resistance, and lower measures of beta cell function in youths with T2D. To our knowledge, no previous studies have examined GHR levels in youth-onset T2D. Growth hormone has direct effects on glucose metabolism independent of its primary effector IGF-1, including increased hepatic gluconeogenesis, increased adipocyte lipolysis, decreased peripheral glucose uptake, and modulation of IGFBPs.^27,28,29,30,31,32,33^ Growth hormone binding protein, the extracellular ligand-binding domain of GHR, has been shown to prolong the half-life of GH and attenuate receptor binding to stimulate IGF-1 synthesis in the liver, and thus it may affect glucose metabolism indirectly.^34^ This may explain the inverse association between GHR and IGF-1 that we observed. Increased GHR signaling in pancreatic islets has been shown to regulate pubertal beta cell expansion in rats.^35^ However, little is known about the association of plasma GHR levels with systemic metabolism. Plasma GHR levels were recently reported to be associated with incident T2D in a large cohort study,^36^ and reductions in GHR levels were identified as a causal mediator of improvements in glycemia after bariatric surgery in adults with T2D.^37^ Prior studies showed that circulating GHR positively correlates with hepatosteatosis and adiposity in children with obesity,^38^ and we did find in our exploratory analyses that GHR level is positively associated with both BMI *z* score and fat mass in both sexes. Therefore, it is possible that an increase in GHR level due to increased BMI and insulin resistance in individuals with T2D may attenuate GH activity in the liver to stimulate IGF-1 synthesis. However, our analysis included an adjustment for BMI *z* score, suggesting that associations between GHR level and glycemic outcomes exist independent of BMI. Our study has identified GHR level as a novel biomarker of decrease in glycemic control in youths with T2D. Future studies to elucidate mechanisms are needed.

At baseline in the TODAY trial, IGFBP-1 level was positively associated with insulin sensitivity (1/C-peptide). This finding is consistent with prior studies indicating that insulin directly suppresses hepatocyte IGFBP-1 production^39^ and that IGFBP-1 level is associated with insulin sensitivity.^20,40^ One study previously demonstrated that IGFBP-1 levels are lower in youths with new-onset T2D and hyperinsulinemia.^41^ As previously reported in the TODAY trial, IGFBP-1 level was inversely associated with BMI.^18^ Increases in IGFBP-1 levels between baseline and 36 months were associated with higher glycemic measures (HbA_1c_ and fasting glucose levels), likely due to loss of beta cell function and consequent loss of the inhibitory association of insulin with IGFBP-1 production.^42^ Insulin-like growth factor binding protein 1 modulates IGF-1 bioavailability and has been shown to inhibit IGF-1 effects, which may explain the inverse association between the 2 hormones in our analysis.^43^ Insulin-like growth factor binding protein 1, as well as IGFBP-2 through 5, may have IGF-independent associations with glucose uptake and insulin sensitivity and have been associated with the pathogenesis of metabolic syndrome, diabetes, and diabetes complications.^11,12,44,45,46,47,48,49^

### Strengths and Limitations

Our study has some strengths, including the use of the TODAY cohort, which captures a large and diverse population of youths with T2D with longitudinal glycemic assessments. Paired comparisons at baseline and 36 months minimized potential confounding due to interindividual differences. We additionally performed linear and logistic regression models to adjust for confounders.

Our study also has several limitations. First, the TODAY study compared treatment options for youths with T2D and therefore did not include a control group without diabetes. Second, a large proportion of the cohort was in late puberty or after puberty at baseline, which limited our ability to perform subgroup analyses based on Tanner staging. However, analyses with adjustment for Tanner stage did not alter the significance of associations. Third, we examined hormone levels only at baseline and at 36 months, and most of our subcohort reached the primary outcome prior to the second measurement of GH mediators. As such, we cannot infer a direction of causality between GH mediator changes and glycemic outcome in the logistic regressions using primary outcome as the outcome measure. Analysis of additional time points earlier in the disease course would help to answer this important question. Furthermore, we noted baseline differences in glycemia and beta cell function between the glycemic failure and glycemic control groups; however, we accounted for baseline HbA_1c_ and beta cell measures as a covariate in our regression analyses. For measurement of insulin sensitivity, we used 1/fasting C-peptide, which correlates well with criterion standard insulin sensitivity measures^15^ but may be suboptimal in individuals with decreased beta cell function. As such, we corroborated our analysis by using adiponectin as a secondary measure of insulin sensitivity. Fourth, our study is limited to the measurement of circulating GH mediators, so we are unable to directly identify the source tissue and target organs of hormone action. Future studies should aim to assess gene expression of IGF-1, GHR, and IGFBP-1 in liver, fat, and other tissues in association with glycemic measures.

## Conclusions

In this post hoc secondary analysis of the TODAY randomized clinical trial, we found novel associations of plasma GH mediator levels with glycemic failure, measures of glycemia, insulin sensitivity, and beta cell function in youths with T2D. However, additional mechanistic or longitudinal studies would help to elucidate the mechanism by which GH mediators affect glycemia, or vice versa. Whether alterations in GH mediators may facilitate progression from prediabetes to T2D or accelerated onset of diabetes complications in youth-onset T2D are important topics for future studies.

## Notes

Supplement 1.
Study Protocol

Supplement 2. **eFigure 1.** CONSORT Study Flow Diagram of the TODAY Secondary Analysis **eFigure 2.** Time-to-Event Survival Analysis of the TODAY Substudy Cohort **eTable 1.** Anthropometric, Demographic, and Clinical Characteristics at Study Entry Based on Primary Outcome in TODAY **eTable 2.** Anthropometric, Demographic, and Clinical Characteristics Compared Between Subcohort With Only Baseline Samples and Subcohort With Available Samples at Baseline and 36 Months **eTable 3.** Glycemic Measures at 36-Month Study Timepoint Based on Primary Outcome in TODAY **eTable 4.** Glycemic Measures at 36-Month Study Timepoint Compared Between Subcohort With Only Baseline Samples and Subcohort With Available Samples at Baseline and 36 Months **eFigure 3.** Correlations Between GH Mediators

Supplement 3.
Data Sharing Statement
