# Supplementary material for: Growth Hormone Mediators and Glycemic Control in Youths With Type 2 Diabetes: A Secondary Analysis of a Randomized Clinical Trial
Source: JAMA Netw Open. 2024 Feb 29;7(2):e240447. doi: 10.1001/jamanetworkopen.2024.0447 (PMC10905312; doi:10.1001/jamanetworkopen.2024.0447)
Supplement: Supplement 1. — Study Protocol [file jamanetwopen-e240447-s001.pdf]

Studies to Treat Or Prevent Pediatric Type 2 Diabetes

STOPP-T2D

TREATMENT PROTOCOL

Treatment Options for type 2 Diabetes in Adolescents and Youth

Sponsored by
National Institute of Diabetes and Digestive and Kidney Diseases
National Institutes of Health

Distributed by
STOPP-T2D Coordinating Center
George Washington University Biostatistics Center
6110 Executive Boulevard, Suite 750
Rockville, MD 20852

version 1.9
January 24, 2011

Table of Contents

1 Introduction and Rationale 1
1.1 Specific Aims and Objectives 1
1.2 Overall Design and Study Interventions 1
1.3 Background and Significance 2
1.3.1 Type 2 Diabetes in Children and Adolescents 2
1.3.2 Pharmacological Treatment of Pediatric Type 2 Diabetes 3
1.3.3 Metformin 4
1.3.4 Thiazolidinediones (TZD) 4
1.3.5 Intensive Lifestyle Intervention 5
2 Outcomes and Objectives 6
2.1 Primary 6
2.2 Secondary 7
2.2.1 Glycemic Control 7
2.2.2 Safety 7
2.2.3 Insulin Sensitivity and Secretion 7
2.2.4 Body Composition 8
2.2.5 Nutrition 9
2.2.6 Physical Activity 10
2.2.7 Physical Fitness 10
2.2.8 Cardiovascular Risk Factors 11
2.2.9 Microvascular Complications 12
2.2.10 Cardiac Function 13
2.2.11 Quality of Life and Psychological Measures 13
2.2.12 Resource Utilization and Costs 14
2.2.13 Gene Expression 14
2.2.14 Pregnancy Outcomes 15
3 Recruitment, Screening, and Enrollment 15
3.1 Recruitment Goals and Strategies 15
3.2 Screening Procedures 15
3.2.1 Staged Screening 16
3.2.2 Informed Consent and Assent 16
3.2.3 Eligibility Criteria Prior to Run-in 17
3.2.4 Eligibility Criteria After Run-in Prior to Randomization 18
3.3 Randomization 19
4 Treatment Administration and Patient Management 19
4.1 Pre-randomization (Run-in) Period 19
4.1.1 Medical Management 20
4.1.2 Standard Diabetes Education 21
4.1.3 Demonstration of Adherence 22
4.2 Post-randomization Period 22
4.2.1 Medical Management 22
4.2.2 Hypoglycemia 23
4.2.3 Ongoing Standard Diabetes Education 23
4.3 Masking 23
4.4 TODAY Lifestyle Program (TLP) 24
4.4.1 Program Goals 24
4.4.2 Program Description 24
4.4.3 Program Components 25
4.4.4 Treatment Structure 27

4.4.5 Resource Needs ... 27
4.5 Patient Reinforcement ... 28
4.5.1 Behaviors Targeted for Reinforcement of Medical Management ... 28
4.5.2 Behaviors Targeted for Reinforcement of Intensive Lifestyle Intervention ... 28
4.5.3 Implementation of the Incentive System ... 28
4.6 Adjunct Care ... 28
4.6.1 Treatment of Dyslipidemia ... 29
4.6.2 Treatment of Hypertension ... 29
4.7 Add-on Insulin Therapy ... 29
4.8 Pregnancy and Sexual Activity ... 31
5 Research Procedures and Approach ... 32
5.1 Data Collection ... 32
5.2 Outcomes Assessment Pilot ... 34
5.3 Participant Retention Program ... 34
5.4 Confidentiality ... 35
6 Safety and Monitoring ... 36
6.1 Data Safety Monitoring Board (DSMB) ... 36
6.2 Safety Monitoring and Risk Management ... 36
6.2.1 Depression ... 36
6.2.2 Laboratory Monitoring ... 36
6.2.3 Potential Risks ... 37
6.2.4 Procedures to Minimize Risks ... 37
6.3 Unmasking ... 40
6.4 Adverse Event and Serious Adverse Event Reporting ... 40
6.4.1 Purpose of Adverse Event Reporting ... 40
6.4.2 Definitions of Adverse Events ... 41
6.4.3 Non-serious Adverse Events ... 41
6.4.4 Serious Adverse Events ... 42
6.4.5 Tracking of Adverse Events by the Study Group ... 42
7 Data Processing and Management ... 43
7.1 Data Management System ... 43
7.2 Data Transfer ... 43
7.3 Central Data Management System ... 43
7.4 Quality Control ... 43
7.5 Backup, Data Security, and Confidentiality ... 44
7.6 Tracking Study Progress ... 44
7.7 Archival and Study Close-out ... 44
8 Statistical Considerations ... 44
8.1 Sample Size ... 44
8.2 Statistical Analysis ... 45
9 Study Administration ... 46
9.1 Organization ... 46
9.2 Central Laboratories and Reading Centers ... 47
9.3 Training and Certification ... 48
9.4 Site Visits ... 48
9.5 Study Website ... 48
9.6 Conflict of Interest Policy ... 49
9.7 Publications and Presentations Policy ... 49
9.8 Protocol Amendments ... 49
10 Literature Cited ... 49

Abbreviations Used

ADA	American Diabetes Association
AE	Adverse Event
BDI	Beck Depression Inventory
BP	Blood Pressure
BUN	Blood Urea Nitrogen
CBL	Central Blood Laboratory
CDE	Certified Diabetes Educator
CDI	Children’s Depression Inventory
CHQ	Child Health Questionnaire
CoC	Coordinating Center
CSA	Computer Science and Applications, Inc. (accelerometer)
DKA	Diabetic Ketoacidosis
DSMB	Data Safety Monitoring Board
DXA	Dual-energy X-ray Absorptiometry
EDEQ	Eating Disorders Examination Questionnaire
FBG	Fasting Blood Glucose
FDA	Food and Drug Administration
FES	Family Environment Scale
FFQ	Food Frequency Questionnaire
GXT	Graded Exercise Test
HOMA	Homeostasis Model Assessment
IRB	Institutional Review Board
LMC	Lifestyle Materials Core
MNSI	Michigan Neuropathy Screening Instrument
MOP	Manual of Procedures
MVPA	Moderate to Vigorous Physical Activity
NAFLD	Non Alcoholic Fatty Liver Disease
NIDDK	National Institute of Diabetes and Digestive and Kidney Diseases
NIH	National Institutes of Health
OGTT	Oral Glucose Tolerance Test
PAL	Personal Activity/nutrition Leader
PCOS	Polycystic Ovarian Syndrome
PDPAR	Previous Day Physical Activity Recall
PEDS QL	Pediatric Quality of Life
PWC	Physical Work Capacity
QEWP-R	Questionnaire on Eating and Weight Pattern–Revised
QoL	Quality of Life
QUICKI	Quantitative Insulin Sensitivity Check Index
SAE	Serious Adverse Event
SDE	Standard Diabetes Education
SMBG	Self Monitoring Blood Glucose
T1DM	Type 1 Diabetes Mellitus
T2DM	Type 2 Diabetes Mellitus
TLP	TODAY Lifestyle Program
TZD	Thiazolidinediones
UKPDS	United Kingdom Prospective Diabetes Study
ULN	Upper Limit of Normal
YRBS	Youth Risk Behavior Survey

1 Introduction and Rationale

The National Institute of Diabetes and Digestive and Kidney Diseases (NIDDK) of the National Institutes of Health (NIH) has sponsored a collaborative agreement entitled Studies to Treat Or Prevent Pediatric Type 2 Diabetes (STOPP-T2D) to conduct a clinical treatment trial, Treatment Options for type 2 Diabetes in Adolescents and Youth (TODAY), and a school-based primary prevention trial of type 2 diabetes (T2DM) in children and youth. The STOPP-T2D Steering Committee has appointed two committees to address these efforts: (1) the Treatment Protocol Committee and (2) the Prevention Protocol Committee. The TODAY Treatment Protocol Committee is composed of investigators associated with the 15 clinical centers (Baylor College of Medicine, Case Western Reserve University, Children's Hospital Los Angeles, Children's Hospital of Philadelphia, Children's Hospital of Pittsburgh, Columbia University, Joslin Diabetes Center, Massachusetts General Hospital, Saint Louis University, State University of New York Upstate Medical University, University of Colorado Health Sciences Center, University of Oklahoma Health Sciences Center, University of Texas Health Sciences Center at San Antonio, Washington University in St Louis, and Yale University), the NIDDK project office, the coordinating center (George Washington University Biostatistics Center), the study chair, and other experts.

This document is the protocol of the multi-center TODAY trial designed to evaluate the safety and efficacy of three treatment regimens for T2DM in children and youth. The protocol was written by the TODAY Treatment Protocol Committee, approved by an External Review Board, and approved by the Institutional Review Boards (IRB) of each participating clinical center prior to the initiation of recruitment. More detailed study procedures are provided in the study's manual of procedures (MOP).

1.1 Specific Aims and Objectives

The primary objective of the TODAY trial is to compare the efficacy of the three treatment arms on time to treatment failure based on glycemic control. The secondary aims are to:

- compare and evaluate the safety of the three treatment arms;
- compare the effects of the three treatments on the pathophysiology of T2DM with regards to beta cell function and insulin resistance, body composition, nutrition, physical activity and aerobic fitness, cardiovascular risk factors, microvascular complications, cardiac function, quality of life, and psychological outcomes;
- evaluate the influence of individual and family behaviors on treatment response; and
- compare the relative cost effectiveness of the three treatment arms.

1.2 Overall Design and Study Interventions

The three treatment regimens are: (1) metformin alone, (2) metformin plus rosiglitazone, and (3) metformin plus an intensive lifestyle intervention called the TODAY Lifestyle Program (TLP). The study recruits patients over a three-year period and follows patients for a minimum of two years. Patients are randomized within two years of the diagnosis of T2DM. The primary outcome is time to treatment failure as defined below. The anticipated duration of the TODAY trial is seven years, including pre-trial planning and post-trial analysis and reporting.

1.3 Background and Significance

T2DM has dramatically increased throughout the world in many ethnic groups and among people with diverse social and economic backgrounds. Over the last decade, the increase in the number of children and youth with T2DM has been labeled an “epidemic” [ADA 2000]. Before the 1990s, it was rare for most pediatric centers to have patients with T2DM. By 1994, T2DM patients represented up to 16% of new cases of diabetes in children in urban areas [Pinhas-Hamiel et al. 1996], and by 1999, depending on geographic location, the range of percent of new cases due to T2DM was between 8-45% and disproportionately represented in minority populations [Dabelea et al. 1999; Rosenbloom et al. 1999].

T2DM in children and youth, as in adults, is due to the combination of insulin resistance and relative β-cell failure. It appears that there are a host of genetic and environmental risk factors for insulin resistance and limited β-cell reserve. The epidemic of pediatric T2DM is coincident with the rise in the number of children who are overweight or at risk for overweight and with a decrease in the physical activity pattern of youth [Dietz et al. 1998; Goran et al. 1995; Troiano and Flegal 1998; Kimm et al. 2000]. There has been a strong association between T2DM and the onset of puberty, a positive family history of T2DM, and elements of the metabolic syndrome such as acanthosis nigricans and polycystic ovarian syndrome (PCOS) [Arslanian 2000; Arslanian et al. 1994].

Preceding the development of frank diabetes, children and youth experience a period of prediabetes. Prediabetes is defined as either elevated fasting glucose or impaired glucose tolerance [ADA/NIDDK 2002]. Despite the dramatic increase in the number of cases of prediabetes and T2DM in pediatric populations, there have been no published large-scale studies investigating the pathophysiology, treatment, and complications of these disorders in children and youth. The long-term complications and costs associated with T2DM make such studies imperative. Between 1997 and 2002, the estimated cost of diabetes with regard to direct medical cost increased from $44 billion to $92 billion, and the total cost increased from $98 billion to $132 billion [ADA 2003]. The vast majority of monies are spent on the long-term complications of this disorder [ADA 2002]. Since the long-term microvascular and cardiovascular complications relate to duration of diabetes and to control of glycemia, it could be hypothesized that the increasing number of children and youth diagnosed with T2DM, if not effectively treated, could dramatically add to the economic burden of this disease over the ensuing decades.

1.3.1 Type 2 Diabetes in Children and Adolescents

Except in American Indian youth, there are no population-based data available with regard to prevalence of T2DM. Instead, only clinic-based reports indicate that there has been a tremendous increase in the number of children and adolescents with T2DM. A ten-fold increase in T2DM in African American children during the period 1982-1994 was reported by Pinhas-Hamiel et al. [1996]. By 1994, they reported that a third of new cases of diabetes in children and youth were attributed to T2DM, with an odds-ratio of developing the disease of 6.1 in African American girls and 3.5 in African American boys compared to Caucasians. In urban Allegheny County, Pennsylvania, by the middle of the 1990s, there was a three-fold increase in diabetes in nonwhites compared to whites and a two-fold increase from the decade before, presumably due to an increase in T2DM in that cohort [Libman et al. 1998]. In Ventura County, California, in Mexican American youth < 17 years of age with diabetes, 31% were diagnosed with T2DM [Neufeld et al. 1998]. The prevalence of T2DM in American Indian/Native American and Native Canadian youth is higher than amongst the other ethnic minorities [Dean et al. 1998; Kim et al. 1999; Savage et al. 1979].

In 1996, the Indian Health Service estimated diabetes prevalence for 15-19 year olds to be 0.45%, an increase of 54% from 1988 [Rios Burrows et al. 1999]. It appears that not only are American Indian/Native Americans and Native Canadians at highest risk to develop T2DM, but that their outcome is poor. Dean et al. [2002] reported that in adults 18-33 years of age who were diagnosed before age 17 years, there was a 9% mortality rate during the observation period, a 6.3% dialysis rate, and a 38% pregnancy loss due to poor glycemic control. In all these groups, there is a skewed female:male sex ratio. For Native Americans it is estimated to be as high as 4-6:1, while for other groups, it is closer to 1.7:1 for females compared to males [Fagot-Campagna et al. 2000].

T2DM occurs almost exclusively in children and youth who are overweight or at risk for overweight (BMI > 85th percentile for age). At the time of diagnosis, most pediatric patients are in the midst of Tanner Stage 2-4 puberty. Puberty contributes to insulin resistance due to augmentation of growth hormone secretion, and if these normal pubertal physiologic changes are not compensated for by increased insulin secretion, frank diabetes will develop. Half to three-quarters of patients have a parent and close to ninety percent have at least one first or second degree relative with T2DM. The clinical presentation of T2DM in youth ranges from mild asymptomatic hyperglycemia to severe ketoacidosis [Silverstein and Rosenbloom 2000; Fagot Campagna et al. 2000; ADA 2000]. In those who present with clinical symptoms due to hyperglycemia, glycosuria and weight loss are present in 20-40%, ketonuria is present in 33% and ketoacidosis is found in 5-10% [Fagot-Campagna et al. 2000; ADA 2000]. Patients without clinical symptoms are diagnosed as the result of routine blood or urine testing during a health care visit or by investigating a variety of complaints such as chronic infection, sleep apnea, hyperlipidemia, hypertension, and hirsutism or irregular periods associated with PCOS. It may be difficult to distinguish T1DM from T2DM at presentation. The absence of autoantibodies is a prerequisite for the diagnosis of T2DM [ADA 2000]. In addition, evidence of residual insulin secretion is suggestive of T2DM rather than T1DM.

Patients with T2DM have dual abnormalities of insulin resistance and insulin deficiency. It is hypothesized that to achieve the level of glycemic control required to optimize long-term outcome and decrease or prevent microvascular complications, treatment regimens should theoretically be designed to improve insulin resistance and preserve residual β-cell function. The available anti-diabetic agents have not been adequately evaluated in pediatric patients. This is particularly relevant with regard to using combination therapy to improve glycemic control or lifestyle interventions aimed at obesity and sedentary behavior.

1.3.2 Pharmacological Treatment of Pediatric Type 2 Diabetes

The available treatment regimens for pediatric patients with T2DM include diabetes education and counseling, the setting of glycemic targets and the institution of pharmacotherapy [Jones 1998; Silverstein and Rosenbloom 2000; Kaufman 2003]. Patients and their families should be made aware of the benefits of increasing physical activity, following an appropriate nutrition plan and adhering to the diabetes regimen. The pharmacologic treatment of T2DM in pediatric patients has not been systematically studied. Currently, many affected youth with T2DM are initially placed on monotherapy with insulin or metformin [Jones et al. 2002; Silverstein and Rosenbloom 2000] as these agents are approved for use in children. However, over the long-term, most patients on monotherapy do not continue to achieve glycemic targets [UKPDS 1998; Lebovitz 1999]. Other drugs currently available for the treatment of T2DM in adults have not been well studied in youth. In addition, studies in adults suggest that although the addition of a second agent can improve glycemic control temporarily, most patients on multiple drug regimens fail to sustain long-term good glycemic control. Studies are needed to better delineate optimal treatment

regimens for children with T2DM. The pharmacologic therapies for this study, which include using metformin alone and metformin in combination with rosiglitazone, were chosen because metformin is approved in pediatrics and because theoretically both of these agents improve insulin sensitivity. Additional agents were not chosen because the estimated number of patients available for recruitment would not support a trial with more than three arms.

1.3.3 Metformin

Metformin improves glycemic control by improving hepatic insulin sensitivity and lowering hepatic glucose production [DeFronzo et al. 1991; Stumvoll et al. 1995; Johnson et al. 1993; Wollen and Bailey 1998; Cusi et al. 1996]. Metformin has been used in the clinical arena for more than four decades, and it has been demonstrated to have adequate safety and efficacy. It is the only oral agent approved by the FDA for use in children and is considered first-line therapy by most pediatric endocrinologists [ADA 2000]. In the 1999 multi-center trial submitted to the FDA for approval in pediatric patients, metformin significantly lowered fasting plasma glucose and HbA1c in drug-naïve patients compared to placebo [Jones et al. 2002]. In 82 patients 10-16 years of age, metformin at a dosage up to 1,000 mg twice daily was used for as long as 16 weeks in a randomized double-blind placebo-controlled trial. Incident cases were enrolled if they had fasting plasma glucose levels between 126-240 mg/dL, BMI > 50th percentile for age, C-peptide ≥ 0.5 nmol/L and HbA1c ≥ 7.0%. Metformin significantly improved glycemic control with an adjusted mean change from baseline in fasting plasma glucose of -42.9 mg/dL for the metformin group compared with +21.4 mg/dL for the placebo group ($p<0.001$). There was also a significant difference in HbA1c in the metformin group compared to the placebo group (7.5 vs 8.6%; $p < 0.001$). This was accomplished without an increase in adverse events. Since most patients with T2DM are obese, an added benefit with metformin therapy is the lack of weight gain [Stumvoll et al. 1995; Bailey and Turner 1996; Campbell and Howlett 1995; Garber et al. 1997; Fontbonne et al. 1996]. Other established benefits include improvement in dyslipidemia, lowering of fibrinolytic abnormalities, and amelioration of PCOS [Jeppesen et al. 1994; Bailey and Turner 1996; Perriello et al. 1994].

The adverse effects of metformin include development of lactic acidosis, which is rare but potentially life-threatening [Bailey and Turner 1996; UKPDS 1998]. Lactic acidosis has generally occurred in the presence of severe renal disease or cardiac failure, which are unlikely to be present in pediatric patients. More commonly, metformin may cause gastrointestinal disturbance [DeFronzo and Goodman 1995]. Although this may occur in up to 15-30% of those who take the drug, discontinuation of treatment for this side effect is only required in approximately 5% of patients.

1.3.4 Thiazolidinediones (TZD)

The thiazolidinediones (TZD) represent a class of oral antidiabetic agents that have been shown to improve metabolic control in patients with T2DM [DeFronzo 1999]. The glucose lowering effect of this class of drugs is mediated through an improvement of insulin sensitivity [Kemnitz et al. 1994; Saltiel and Olefsky 1996; Miyazaki et al. 2001; Aronoff et al. 2000; Phillips et al. 2001]. TZDs reduce insulin resistance in adipose tissue, muscle, and liver.

TZDs are high affinity ligands of the gamma isoform of the peroxisome proliferator-activated receptor (PPARγ), a member of the nuclear receptor super family of transcription factors. PPARγ is predominantly expressed in an adipose-selective manner in both rodents

and humans, although it is also expressed in other organs, including skeletal muscle and liver. The clinical potency of available TZDs has been shown to correlate closely with their ability to bind to the PPARγ receptor [Olefsky and Saltiel 2000].

Besides its insulin-sensitizing ability, one theoretical advantage of using TZD as a therapeutic agent in T2DM is the potential that these agents might preserve pancreatic insulin secretion. The mechanism by which TZDs might preserve insulin secretion is not fully understood; however, hypothetically the preservation of pancreatic function might occur through amelioration of lipotoxicity and lowering of FFA levels [Girard 2000; Greene 1999; McGarry and Dobbins 1999; Unger and Orci 2000]. Preservation of beta cell function is a key aspect of diabetes control since one of the two major pathogenic factors leading to hyperglycemia in diabetes is a reduction in insulin secretion when pancreatic beta cells can no longer compensate for insulin resistance by producing elevated levels of insulin.

Currently, there are two TZDs available and FDA approved in the US for the treatment of adult T2DM: (1) rosiglitazone (Avandia, Glaxo-Smith Kline) and (2) pioglitazone (Actos, Lilly/Takeda). The effective clinical dose for rosiglitazone is 4-8 mg/day while for pioglitazone it is 15-45 mg/day. Rosiglitazone is available in a combination pill with metformin that has been approved for use in adults with T2DM. The known side effects of this class of drugs include liver toxicity, fluid retention/edema, congestive heart failure, anemia, and weight gain. Although hepatic toxicity has been observed with troglitazone, which is no longer commercially available for human use, similar toxicity has not been observed so far with the second generation TZDs rosiglitazone or pioglitazone. Edema and congestive failure usually occur with a background of cardiac or renal disease—comorbidities less likely found in pediatric patients within two years of diabetes diagnosis. Rosiglitazone has also been associated with weight gain and fat redistribution in some patients [Fonseca et al. 2000]. In vitro data suggest that rosiglitazone is a potent trigger for pre-adipocyte differentiation [Tontonoz et al. 2000]. However, in humans, some studies indicate that, although rosiglitazone may cause an increase in subcutaneous fat, visceral fat and the ratio of visceral fat to subcutaneous fat may be decreased [Adams et al. 1997; Miyazaki et al. 2002].

At present, there is a multi-center clinical trial of rosiglitazone in youth to determine safety and efficacy. As in adult studies, there is no evidence of hepatic toxicity with rosiglitazone. There do not appear to be specific concerns about the safety of the TZDs in youth compared to adults. The combination of TZD and metformin therapies may improve insulin sensitivity in pediatric patients and be well tolerated, as it is in adults [Fonseca et al. 2000].

1.3.5 Intensive Lifestyle Intervention

Obesity is a major problem throughout the world with a significant proportion of US youth being overweight. It has recently been reported that 20% of children and youth in the U.S. are overweight (BMI > 95th percentile for gender and age) or at risk for overweight (BMI > 85th percentile for gender and age) [National Center for Health Statistics 1994; Department of Health and Human Services 1996]. There is ample evidence that children become overweight because of the interplay between genetic factors, excess energy intake from high fat, high calorie, low nutrient diets, and sedentary behaviors with too much time spent watching TV and playing video games [Kimm et al. 2000; Coditz et al. 1990; Manson et al. 1991; West and Kalbfleisch 1971]. Once overweight, children and youth may develop hyperinsulinemia and decreased insulin sensitivity, putting them at risk to develop T2DM.

In the treatment of T2DM in adults, it is beneficial to decrease insulin resistance by reducing body weight via a lifestyle program focused on the development of healthier dietary

and physical activity habits. A number of adult studies have shown that weight loss associated with improvements in eating behavior, diet, and physical activity have resulted in significant reductions in fasting plasma glucose and insulin levels, hepatic glucose output, and peripheral insulin resistance, hypertension, and dyslipidemia [Blackburn 1995; Goldstein 1992; Wing et al. 1987; Maggio and Pi-Sunyer 1997; Henry 1986]. Three uncontrolled trials in adults with T2DM treated with oral agents have shown the benefit of weight loss associated with lifestyle modification on reducing mortality [Wing et al. 1987; Lean et al. 1990; Chaturvedi and Fuller 1995]. The LookAHEAD trial is an ongoing NIH funded multi-site controlled trial of adult patients with T2DM and obesity. This trial is examining the impact of lifestyle modification (changes in eating and activity) and weight loss compared with standard care on morbidity and mortality. As the epidemic of T2DM in children and youth is relatively recent, there is little controlled evidence regarding the use of lifestyle modification to improve insulin sensitivity and glycemic control, induce weight loss, or affect other outcome measures, such as dyslipidemia and hypertension, in pediatric patients with T2DM.

2 Outcomes and Objectives

2.1 Primary

The primary objective of the TODAY trial is to compare the three treatment arms on time to treatment failure in patients enrolled from 10 to 17 years of age with T2DM. The study is analyzed as an ‘intention to treat’ study enrolling 750 patients (250 per arm). The study is powered to allow all three possible comparisons between the treatment groups while maintaining the overall significance level at 0.05. The three treatment group comparisons are: (1) metformin alone versus metformin plus intensive lifestyle program, (2) metformin alone versus metformin plus rosiglitazone, and (3) metformin plus intensive lifestyle program versus metformin plus rosiglitazone. Treatment failure is defined in one of two ways:

1. **HbA1c ≥ 8% over a 6-month period.** All regularly scheduled HbA1c values must be ≥ 8% over a 6-month period. If any one value is < 8%, after which HbA1c re-elevates to ≥ 8%, the clock will restart at the time of the re-elevation. At least two consecutive measurements must be ≥ 8% over 6 months.
2. **Inability to wean from temporary insulin therapy due to metabolic decompensation.** Participants who experience metabolic decompensation requiring temporary use of insulin, who cannot safely be weaned from insulin within three months, will be classified as treatment failures (see section 4.7).

The primary outcome of treatment failure is defined in terms of HbA1c, because it correlates with glycemic control and long-term diabetes outcome. Because HbA1c is the primary outcome, investigators and patients are blinded to HbA1c values. In addition, patients with abnormal reticulocyte count or HbA1c chromatogram indicating the presence of abnormal hemoglobin variants other than heterozygosity for S and C at the time of initial screening are excluded from participation due to interference with HbA1c assays. Homozygous S and C and S/C patients are excluded. These patients are referred for appropriate hematologic evaluation. Management of patients with metabolic decompensation requires insulin treatment. However, if insulin treatment cannot be withdrawn after three months, this is considered a failure of the primary therapy.

2.2 Secondary

There are a number of secondary outcomes in this trial. The results of these secondary outcomes help interpret the primary effect of the treatment regimens on HbA1c. These secondary aims have been chosen because they provide insight into the mechanism by which the treatment regimens affect durable glycemic control (e.g., effects on insulin resistance, sensitivity, diet and physical fitness) or because they provide information concerning the differential risks and benefits of the three treatment arms (e.g., studies of microvascular complications and cardiovascular risk). Data are collected from the child, the biological parents, and a designated family support person, or FSP, who participates in the intervention with the child (may be the same as the child's parent or guardian). The following sections describe the various outcome measures selected for the TODAY trial.

2.2.1 Glycemic Control

Mean HbA1c levels for the three treatment arms are compared throughout the trial as measures of the degree and durability of glycemic control. The overall goal is to maintain HbA1c levels as close to the normal range as possible in order to reduce long-term diabetes complications. During the trial, the investigator and the participant are blinded to HbA1c results. However, both are informed if (1) HbA1c ≥ 8%, (2) HbA1c is between 6-8% and has increased ≥ 0.8% (as determined by a difference from the prior visit value), or (3) HbA1c is at the target of ≤ 6%. This alert system allows clinicians to work with participants and families to reinforce adherence with the treatment protocol and to ensure safety.

In order to gain greater insight into possible differences in glycemic control between the treatment arms and to provide greater understanding of the durability or loss of targeted glycemic control, data are collected regarding daily fingerstick blood glucose readings.

Participants are taught self-monitoring of blood glucose levels and obtain blood glucose readings two times per day—in the morning on awakening (fasting) and one additional time (e.g., before dinner, lunch, two hours after dinner) each day. Additional testing is recommended during periods of illness. At all clinic visits, meters are downloaded; the number of tests performed per day as well as the 14-day and 30-day average glucose levels are recorded and compared for the three treatment arms.

In addition, participants are asked to obtain home glucose levels fasting and two hours following lunch and dinner for two days of the week before each clinic visit. Values are analyzed for mean fasting and postprandial levels and with regard to the incidence of postprandial hyperglycemia.

2.2.2 Safety

Comparisons are made with regard to the number of patients with treatment-related side effects among the three treatment arms. Abnormalities in laboratory tests (hemoglobin/hematocrit, liver function tests, calculated creatinine clearance), episodes of severe hypoglycemia, and incidence of side effects (e.g., gastrointestinal complaints, edema, weight gain) are tracked as outlined in detail in the chapter on safety and monitoring.

2.2.3 Insulin Sensitivity and Secretion

The efficacy and durability of the successful treatment of T2DM is determined to a great extent by the ability of a specific intervention to ameliorate insulin resistance and prolong or

restore effective beta cell function. There is significant information available on the natural history of insulin resistance and secretion, and on the effect of various treatment regimens on secretion and sensitivity in adult patients with T2DM. Little is currently known about these natural history and treatment issues in affected children and youth. In particular, significant questions remain about the impact of advancing pubertal status on insulin resistance and beta cell function in pediatric patients with T2DM. Therefore, an important component of TODAY is to determine (1) the influence of baseline insulin sensitivity and secretion on the response to therapy and (2) the effect of each therapy on the progression of changes in insulin sensitivity and secretion.

To determine the optimal assessment of insulin sensitivity and secretion in this large cohort of study patients, a number of factors were taken into consideration. The glucose clamp technique and the frequently sampled intravenous glucose tolerance test with minimal model analysis are considered the ‘gold standard’ for determination of insulin sensitivity and secretion. However, these are labor intensive, time consuming, and costly procedures that are not easily implemented in large clinical trials. A number of simpler methods have been developed that are preferable in the context of large clinical trials such as TODAY. The oral glucose tolerance test (OGTT), homeostasis model assessment (HOMA), and the quantitative insulin sensitivity check index (QUICKI) are used to compare insulin secretion and sensitivity among the three treatment groups. Use of these multiple measures helps compensate for not using the ‘gold standard’ measures as listed above. Values of glucose and insulin/C-peptide derived from the OGTT, when combined with anthropometric parameters, can predict insulin sensitivity and secretion indices derived from clamp measurements with reasonable accuracy [Breda et al. 2002; Stumvoll et al. 2000; Guzzaloni 2002]. The OGTT-derived equations that assess insulin sensitivity take into account BMI, 120-minute insulin levels, and 90-minute glucose values. The assessment of first phase insulin secretion is done with the fasting and 60 minute insulin concentrations.

A number of indices based on fasting measurement of glucose and glucose regulating hormones such as insulin (including C-peptide) and proinsulin have also been proven to closely correlate with corresponding clamp-derived indices of insulin sensitivity and secretion in diverse pediatric populations [Bonora et al. 2000; Hermans et al. 1999; Katz 2000; Guzzaloni 2002; Uwaifo 2002]. Since it is easy to obtain fasting blood tests, evaluation of these measurements to assess insulin sensitivity and secretion is ideal for a large clinical trial. HOMA-R (resistance) and B (secretion) are calculated using a computer-based model solution. QUICKI is calculated as $1/(\log[I_0] + \log[G_0])$.

Insulin sensitivity and secretion are determined with fasting glucose, insulin, C-peptide, and proinsulin levels, OGTT, HOMA, and QUICKI at baseline, 6 months, annually, primary endpoint, and end of study and are compared among the three treatment arms.

2.2.4 Body Composition

In children and adolescents, obesity is associated with insulin resistance, hypertension, dyslipidemia, non-alcoholic fatty liver disease (NAFLD), and left ventricular hypertrophy. The development of insulin resistance during puberty is most closely correlated to fat mass, independent of insulin, sex steroids, leptin, or IGF-1 status [Roemmich et al. 2002]. Obesity causes an increase in intramuscular triglyceride content in adolescents relative to lean controls that is associated with a decrease in insulin sensitivity [Sinha et al. 2002]. Weight loss in overweight adolescents improves both insulin sensitivity [Kay et al. 2001] and blood pressure [Brownell et al. 1983]. However, there are scarce data describing the effects of weight loss on these parameters in children or adolescents with diabetes. An important secondary outcome of TODAY is the investigation of whether weight loss, in particular a decrease in adiposity, improves glycemic control, insulin sensitivity, cardiovascular risk

factors, and blood pressure in children and adolescents with T2DM as it does in the adult diabetes population and in overweight adolescents. An accurate measurement of body fat is essential during this trial to determine the effectiveness of the lifestyle intervention, which focuses on weight loss, as well as to help clarify the mechanism of effect.

Overweight status and body composition are assessed by standardized anthropometric measurements (height, weight, BMI) as well as waist circumference, and abdominal height measured laterally with the patient supine [Pouliot et al. 1994]. Percent body fat and fat distribution are measured by dual-energy X-ray absorptiometry (DXA). A cross-calibration study of different body composition techniques versus DXA in children has shown that skinfold thickness measurements, bioelectrical impedence, and various combinations of anthropometric measures are highly inaccurate when compared to DXA [Goran et al. 1996]. Therefore, DXA is the main measure to assess the effect of the three treatment arms on adiposity. DXA (with pediatric software as appropriate) uses a three-compartment model of total body fat mass, bone mineral mass, and non-bone lean mass. This is of particular importance in a multi-ethnic study since bone mineral content and lean tissue mass differ by gender and ethnicity [Ellis et al. 1998; Cote and Adams 1993].

Height and weight are measured at each study visit. Participants undergo other anthropometric measurements and DXA analysis of body composition at baseline, six months, two years, primary endpoint, and end of study; results are compared among the three treatment arms.

In addition, biological parent height and weight are collected at baseline in order to evaluate the predictive effect of parent BMI on outcomes. Height and weight are collected from the family support person (FSP) on an annual basis in order to determine the effect of FSP weight loss or participation in the lifestyle intervention with participant anthropometrics and body composition.

2.2.5 Nutrition

Hypercaloric, high-fat diets are known risk factors for the development of diabetes, both through promotion of obesity and as an independent risk for insulin resistance. Diet change has been correlated with a decrease in diabetes risk, as well as weight loss, in a number of prevention and intervention studies, including the Diabetes Prevention Program [DPP Research Group 2002]. Therefore, evaluation of baseline diet characteristics, as well as the effect of treatment interventions on dietary change in participants, is considered to be an important secondary outcome of this trial. Diet assessment permits evaluation of the effectiveness of the lifestyle intervention relative to the standard education treatment arms. In addition, dietary assessment over the duration of the trial provides insight into which components of the intervention lead to the most significant and durable change in lifestyle. Such information is critical in translating the results of this study to future trials as well as to clinical practice.

A number of tools can be used to assess dietary intake, a variety of which were considered for this trial. Although there are few validity studies of food frequency approaches in adolescent populations, the available data suggest that validity and reproducibility are comparable to results obtained in adults [Rockett and Colditz 1997]. Recently, the Block Kid's Questionnaire was validated in low-income African-American children. This instrument has been further modified to incorporate common food choices among ethnically and regionally diverse youth aged 10-19 participating in another large childhood diabetes study, SEARCH (SEARCH for Diabetes in Youth, CDC/NIH). Modifications were based on previously validated work with food frequency questionnaire (FFQ) methodology in ethnically diverse populations [Mayer-Davis et al. 1999]. The

instrument is interview-administered and captures the last week of dietary intake. The SEARCH FFQ provides a validated instrument that also enables direct comparisons to the dietary intake of youth with diabetes participating in SEARCH. Dietary assessment is coordinated by the Diet Assessment Center, University of South Carolina, under the direction of Dr. Beth Mayer-Davis, from measurements taken at enrollment, six months, two years, primary endpoint, and end of study.

2.2.6 Physical Activity

Physical activity is comprised of activities that span a spectrum of intensity levels and can be assessed using activity recall questionnaires and/or objective measures of activity, such as pedometers and accelerometers. The most popular activity questionnaire currently used with youth is the Previous Day Physical Activity Recall (PDPAR) which involves completion of a questionnaire that assesses the previous days' activities and their relative intensities for the after school hours [Weston 1997]. The PDPAR analyzes the activity level over a short time frame and may not reflect activity levels that vary with seasons or as a result of an acute illness or time commitment [Kriska 1997]. In combination with accelerometer data, it offers a valuable comparison of differential activity levels across treatment arms. The 3-day PDPAR is completed at baseline, 6-months, two years, primary endpoint, and end of study.

One problem with activity recall measures is that lower intensity activities, such as walking, and subtle lifestyle changes, such as taking the stairs instead of the elevator, are harder to recall than higher intensity activities such as organized sports. Therefore, the activity questionnaire may not accurately quantify changes in lower intensity lifestyle activities [Kriska et al. 1990]. The CSA accelerometer allows the collection and storage of daily patterns of physical activity and is a more complex instrument than the pedometer. These monitors have been validated in a variety of laboratory and field settings. In adults, correlation coefficients ranging from r = 0.66 to r = 0.89 between CSA counts and metabolic measures [Melanson and Freedson 1995] have been obtained. In 7-15 year old boys and girls, the CSA was validated against heart rate telemetry with correlation coefficients between the two ranging from r = 0.50 - 0.74 [Janz 1994]. Comparisons with oxygen consumption during treadmill exercise and self-selected speed on a track found that the CSA was highly related to both and was highly sensitive to change in speed but not changes in grade [Nichols 2000]. Since the CSA has been found to successfully detect bouts of moderate intensity physical activity such as brisk walking [Masse 1999], this monitor is a good choice for TODAY where moderate intensity activity is the goal. Participants are given an accelerometer to wear at home at baseline, 6 months, two years, primary endpoint, and end of study for 7 days at the time of the PDPAR. Verbal and/or written instructions for the monitors are presented to the child with a diary that needs to be completed on the days that the monitor is worn.

2.2.7 Physical Fitness

Physical activity is defined as any bodily movement produced by skeletal muscles that results in energy expenditure. Physical fitness, on the other hand, characterizes the physiologic state of an individual, including aerobic power, muscular endurance, muscular strength, body composition, and/or flexibility. Both physical activity and fitness are independently related to changes in insulin sensitivity and improvement in the pathophysiology of T2DM. Those who are more active and expend more energy have higher fitness levels. However, there is only a moderate relationship between physical

activity and physical fitness [Jacobs 1993]. Therefore, the effect of the treatment interventions on both of these measures is compared.

Cardiorespiratory fitness is defined as the ability to absorb, transport, and use oxygen. Higher maximal oxygen uptake (VO_2 max) values indicate better cardiorespiratory fitness. Ideally it is assessed by direct measurement of oxygen uptake during a graded exercise test (GXT) on a treadmill or cycle ergometer. The measurement of VO_2 max is a challenging task in children [Rowland and Cunningham 1992; Howley 1995]. Motivating youth to exercise maximally is difficult and they may have difficulty obtaining VO_2 max. Furthermore, these tests require expensive metabolic measuring systems and take approximately an hour to complete.

Because of these difficulties, investigators have resorted to predicting VO_2 max. The most popular methodologies to predict VO_2 max in children have utilized cycle ergometry to determine the physical work capacity or PWC [Boreham 1990]. These tests involve obtaining heart rates at three submaximal workloads, plotting these heart rates against the workload, extrapolating to determine the workload at a given heart rate or at maximal heart rate, and then converting that maximal workload to oxygen uptake. Participants sit quietly while their heart rate is measured, until they have adapted to the laboratory and their heart rate is stable. They then begin the multistage fitness test, beginning at 150 kpm and increasing in 150 kpm increments every three minutes until a heart rate of 160 bpm is reached. The work required to produce a heart rate of 160 bpm is used to extrapolate the PWC at a heart rate of 170 bpm. This prediction of VO_2 max has several advantages. First, the PWC equipment and space needs are modest. Second, the PWC can be administered in 12-15 minutes and requires only the measurement of heart rate. Third, the PWC is ideal to use in a test-retest protocol since learning, practice, or training have little effect on its results.

In this protocol, participants undergo PWC 170 testing at baseline, six months, two years, primary endpoint, and end of study, and the effects of the three treatment arms on fitness are compared.

2.2.8 Cardiovascular Risk Factors

Both obesity and T2DM are associated with a significantly increased risk of cardiovascular morbidity and mortality in adults, and insulin resistance, present in both conditions, has been considered the underlying pathologic mechanism. Previous studies on adult populations have consistently demonstrated the associations among insulin resistance, hyperinsulinemia, and the traditional cardiovascular risk factors of hypertension and dyslipidemia [Reaven 1988; Zavaroni et al. 1989; Zavaroni et al. 1990]. However, the association with these traditional risk factors accounts for only approximately 50% of the cardiovascular risk associated with hyperinsulinemia and insulin resistance, an observation that has led to the identification of a number of ‘non-traditional’ risk factors for cardiovascular morbidity [Kullo et al. 2000]. In general, these newly identified risk factors indicate alterations in hemostasis and the presence of acute and chronic systemic inflammation. For example, increased risk of cardiovascular disease has been associated with alterations in the fibrinolytic pathway and other aspects of hemostasis, including regulation of the equilibrium between tissue-type plasminogen activator (t-PA) and its inhibitor plasminogen activator inhibitor–1 (PAI-1), von Willebrand factor, and factor VII antigen [De Maat et al. 1996; Juhan-Vague et al. 1999; Meigs et al. 2000]. Similarly, increased cardiovascular risk has been associated with a wide variety of markers that indicate a state of chronic inflammation, including white blood cell count, C-reactive protein, IL-6, TNF-α, and other acute phase reactants [Kullo et al. 2000]. The recognition of the relationships among these factors has drastically altered our understanding of the pathophysiology of insulin resistant

states and lead to reconsideration of the mechanism of action of a variety of agents, including the insulin-sensitizing thiazolidinediones.

Among children, less is known about the relationship between obesity, insulin resistance, T2DM, and cardiovascular risk. Several large epidemiologic studies in youth have shown an association between obesity, traditional risk factors (e.g., hypertension and dyslipidemia), and cardiovascular disease [Srinivasan et al. 2002; Zieske et al. 2002]. However, little is known about the non-traditional risk factors. In small series, obese children and adolescents have been found to have increased levels of fibrinogen, PAI-1, and D-dimer, as well as abnormalities in factor VIIc, von Willebrand factor, PAI-1, fibrinogen, and tissue plasminogen activator [Ferguson et al. 1998; Gallistl et al. 2000; Sudi et al. 2001]. Following weight loss interventions, decreased levels of PAI-1 and IL-6 have been demonstrated [Estelles et al. 2001; Gallistl et al. 2001] suggesting that lifestyle interventions may be able to alter cardiovascular risk in young patients.

These studies suggest that among obese adolescent patients with T2DM, there may be unfavorable patterns of both traditional and non-traditional risk factors implicated in cardiovascular morbidity and mortality. The development of cardiovascular risk is an important consideration in comparing the efficacy and long-term implications for the treatment interventions under study in this trial. To address this, the differential effects of the three treatment arms on both traditional and non-traditional markers are compared. Blood pressure is measured at every visit and specimens drawn for repeated measurements of lipids (free fatty acids, lipoprotein subclass levels, average LDL particle density, and total ApoB level), fibrinogen, c-reactive protein, plasminogen activator inhibitor-1, homocysteine (vitamin B-12 will be determined to evaluate homocysteine levels), and interleukin-6 at baseline, six months, annually, primary endpoint, and end of study.

2.2.9 Microvascular Complications

Microvascular complications associated with diabetes produce significant burdens for the individual patient and are responsible for a major part of the public health care costs associated with diabetes. Microvascular complications are more common among children with T2DM at the time of presentation than among those with type 1 diabetes (T1DM) [Takahashi et al. 1990; Yokoyama et al. 2000]. There is evidence, although limited, that suggests that progression rates of microvascular complications are also greater in youth with T2DM. Therefore, a comparison of the effect of the treatment interventions on the prevention and slowing of rates of development of microvascular complications associated with T2DM is an important secondary outcome of TODAY and could significantly modify the interpretation of the primary outcome results.

Quantitation of microalbuminuria is performed by obtaining spot urine measurements of microalbumin/creatinine ratio at baseline and annual visits. Abnormal values on spot urines are confirmed with two additional spot urine samples within three months; diagnosis of microalbuminuria is made as a result of two out of three positive tests. Creatinine clearance (by calculation) is determined at baseline and annual visits. Abnormal values are monitored more frequently. Results are compared for the three treatment regimens.

The presence of peripheral neuropathy is evaluated using the Michigan Neuropathy Screening Instrument (MNSI) [Feldman 1994], a simple and well-validated screening tool for detection of peripheral neuropathy in patients with T2DM. MNSI screening is performed at baseline, annual visits, primary endpoint, and end of study.

Retinopathy also appears to have increased in prevalence among children with T2DM at presentation [Yoshida et al. 2001], though data are limited to small case series. Due to financial limitations, routine identification and monitoring for retinopathy was not undertaken as a primary component of TODAY, though participants were encouraged to undergo

screening according to ADA Clinical Practice Guidelines and results of screening were obtained. However, in the final year of the study, all TODAY participants undergo retinopathy screening, including dilated retinal exam and retinal photography. Results are compared for the three treatment regimens.

2.2.10 Cardiac Function

Type 2 diabetes is associated with functional and anatomic changes in the heart in adults, including left ventricular and septal hypertrophy, decreased ejection fraction, and decreased distensibility. However, little is known about the association of type 2 diabetes and alterations in cardiac function in adolescents. Two recent papers [Chinali et al. 2008, Nadeau et al. 2009] report left ventricular hypertrophy and decreased cardiac adaptation to exercise in small groups of adolescents with type 2 diabetes compared to their obese non-diabetic peers. In the last year of TODAY, cardiac function is evaluated in all participants by resting echocardiography. Results are compared for the three treatment regimens.

2.2.11 Quality of Life and Psychological Measures

The psychological, emotional, and social status of a patient interacts with his or her chronic illness in complex ways. Difficulties in the family social or psychological structure can hasten onset of lifestyle related disorders, such as T2DM, and then further interfere with treatment. Onset of a chronic illness, particularly one that requires significant personal change, can adversely affect many aspects of a patient's and family's emotional well being. This intervention trial examines whether psychological characteristics of patients influence treatment outcome and whether the interventions have an effect on psychological problems and quality of life. The following questions are of interest: (1) whether the participant's and/or parents' psychological status at baseline has an important effect on the success of the three treatment arms, (2) whether the treatment assignment has an effect on psychological outcomes related to the diagnosis of diabetes, and (3) whether changes in the family support person's psychological status affect outcomes.

In the child, responses to psychological problems and quality of life measures are compared among the treatment groups at baseline, six months, 2 years, primary endpoint, and end of study using standardized and validated instruments. The specific instruments to assess psychological problems include: the Children's Depression Inventory (CDI) (up to age 16) [Kovacs 1981; Kovacs 1985; Kovacs 1992; Kovacs and Beck 1997; Reynolds 1992], the Beck Depression Inventory (BDI) (beginning at age 16) [Beck et al. 1961; Beck et al. 1988; Beck and Steer 1993; Beck and Steer 1996; Kendall et al. 1987], the Eating Disorders Examination Questionnaire (EDEQ), the Questionnaire on Eating and Weight Pattern–Revised (QEWP-R) (only if indicated by the score on the EDEQ) [Landgraf et al. 1996]. Quality of life is assessed using the Pediatric Quality of Life [Varni et al. 2003] for evaluation of health related quality of life.

The biological parent also completes the BDI, EDEQ, and QEWP-R (only if indicated by the score on the EDEQ) at baseline. The FSP completes the BDI, EDEQ, QEWP-R (only if indicated by the score on the EDEQ), Pediatric Quality of Life (adult proxy) and CHQ (P28) at baseline and 24 months.

At the end of study, a psychosocial inventory is completed by study staff in order to assist participants in their transition out of the TODAY study into regular clinical care and to provide appropriate direction to community resources. These data will also be used to describe the psychosocial challenges experienced by TODAY study participants.

2.2.12 Resource Utilization and Costs

Rising health care costs and limited health care resources have increased the focus placed on the economic aspects of health care. Analyses of resource utilization and costs contribute to decisions made by health service providers and policy makers regarding diabetes care. By incorporating measures of resource utilization, cost, and an intervention's effectiveness, cost-effectiveness analysis has become an important tool in decision making about the use of different treatments for the same condition. Several large trials of diabetes care interventions have demonstrated that enhanced treatment of diabetes improves health at an acceptable cost [DCCT Research Group 1996; Gary et al. 2000; Rodby et al. 1996].

Another secondary outcome of TODAY is an investigation of the resources, related costs, and cost-effectiveness of the three treatment arms. Resource utilization for each of the treatment arms is assessed throughout the trial and considers the intensity of services used in providing care and assuring adherence to each treatment regimen. The frequency of contact with physicians, nurse educators, psychological support staff, dietitians, and the use of drugs, equipment, and supplies are captured through study forms. Information on the time in which each of these health professionals is involved in treatment provision is obtained through surveys completed periodically by the providers.

The provision of care to youth and adolescents affects not just the study participants but also their caregivers. Participant and caregiver burdens may differ for each treatment. To consider this impact, several additional items that incur costs are assessed, including those of school absenteeism, work absenteeism (in children and adults), and the time spent in treatment activities by children and caregivers. These items are measured at baseline and periodically thereafter through surveys utilizing previously validated questions. We also assess academic and employment participation rates and progress since these outcomes may differ among treatment arms. Measurement of quality-adjusted life years is obtained through the HUI-2 (Health Utilities Index) [Furlong et al., 2001], which provides a preference-based measure of quality of life. The HUI-2 is administered at baseline, 6 months, annually, primary endpoint, and end of study.

The cost-effectiveness analysis considers the resources used and associated costs for each treatment arm throughout the time of the trial. The time frame of the analysis is short-term. The main analysis is based on the primary glycemia outcome in the trial and, thus, examines the costs of each treatment relative to the glycemia benefits obtained. By incorporating costs incurred by participants and caregivers, the analysis adopts a societal perspective, following the accepted standard for such studies [Gold et al. 1996; Drummond et al. 1999].

2.2.13 Gene Expression

The role that gene expression plays in the pathophysiology of type 2 diabetes, insulin resistant states, and long-term cardiovascular risk is not well-understood. The cohort of adolescents with type 2 diabetes provides a unique opportunity to establish a resource for exploration of gene-phenotype- outcome relationships. After randomization, we request that participants provide a blood sample for DNA that will be used for future studies of candidate genes for type 2 diabetes, obesity, insulin resistance, and cardiovascular complications of insulin resistance. A separate consent/assent is signed for donation of a DNA sample and refusal does not affect participation in the remainder of the TODAY protocol.

2.2.14 Pregnancy Outcomes

Few data have been reported on pregnancies in this demographic, including both the course of the pregnancy and the neonatal experience as well as outcomes for the mother and the baby. The importance of collecting data goes beyond the need to monitor and document safety in a clinical trial of experimental interventions.

Girls who become pregnant during TODAY are asked to participate in a separate data collection including pre-pregnancy history, care received during pregnancy, complications during the pregnancy, delivery data and perinatal complications. Participants sign a separate informed consent form and a medical release form so that the study can obtain relevant medical records and extract data.

3 Recruitment, Screening, and Enrollment

3.1 Recruitment Goals and Strategies

The primary source of participants for the TODAY study is the active patient population of the study sites and their usual referral sources. Supplemental strategies for increasing referrals (such as presentations to local physician groups) are also utilized. Site-specific recruitment strategies are developed by each local study team. The period of recruitment is anticipated to last four years.

The TODAY sites were chosen partially based on the ethnic/racial composition of their patients with T2DM. It is estimated that the ethnic/racial distribution of enrolled participants will be 33% African American, 31% Hispanic (largely Mexican American), 12% Native American, 20% non-Hispanic White, and 4% other (largely Asian American). Study sites will develop targeted strategies for maximizing the recruitment of the particular racial and ethnic groups seen in clinic.

Recruitment efforts are integrated into and supported by other TODAY activities, including national and local public relations efforts, on-going development and adaptation of recruitment and advertising materials, and presentations at local and national meetings. Recruitment of current patients as well as community-based recruitment will begin in parallel from the start of the enrollment period.

Recruitment progress is monitored regularly by the coordinating center and the Recruitment and Retention Committee. Limited, anonymous data are collected on individuals who refuse to participate in order to tailor the recruitment process. Assistance is offered to clinical centers struggling with recruitment efforts.

3.2 Screening Procedures

Screening for eligibility criteria involves completion of a screening visit and a 2-6 month run-in period. Screening proceeds from least invasive, most easily obtained criteria to the most demanding procedures. Once a patient is determined to be ineligible, the screening process stops.

If the patient is currently deemed ineligible but the patient’s condition changes within the recruitment period (e.g., the patient has uncontrolled hypertension that can be treated), the patient may be reconsidered for inclusion if he/she maintains eligibility for three months. Screening procedures are re-initiated and the patient must meet all eligibility criteria at the time of enrollment.

A minimal amount of data are recorded on patients who fail screening (age group, gender, race/ethnicity, duration of diagnosis) for purposes of reporting progress to the NIDDK and for comparing participants versus nonparticipants.

3.2.1 Staged Screening

The staged screening process is intended to accomplish the following:

- identify potentially eligible patients for TODAY,
- verify eligibility of patients,
- accomplish the objectives of the informed consent process,
- complete a run-in period,
- establish a research cohort likely to complete the study and adhere to the protocol, and
- randomize patients into TODAY.

The staged screening process is a series of steps to evaluate the eligibility of children with T2DM, assess whether patients can safely tolerate metformin treatment, demonstrate adherence to medication taking and record keeping, and provide standard diabetes education (SDE) for all patients (see section below). Patients complete a run-in period to explore their ability to participate, ensure that they can achieve glycemic control and tolerate metformin, and sign a final informed assent/consent for randomization. Complete definitions and procedures are in the Manual of Procedures.

- *Initial Contact:* Identified patients are seen in clinic and are given detailed information about TODAY, including research interventions, randomization, masking, test procedures, risks and benefits, and the eligibility process. If they consent/assent, they are first interviewed and records reviewed for personal and medical history. If they pass these completely noninvasive eligibility criteria, then they continue to be evaluated.

- *Eligibility for Run-in:* Eligibility criteria requiring procedures, tests, and measurements are collected.

- *Run-in:* The run-in period (described in detail in section 4.1) is designed to (1) get the child on the maximum tolerable dose of metformin (no more than 1000 mg bid, no less than 500 mg bid), (2) wean all other diabetic medications, and (3) present a standard diabetes education program.

  It also allows patients and their families to make an informed decision about participation in the study. Participants complete tasks similar to those required of study patients (e.g., taking pills on the correct schedule, completing nutrition and activity diaries, and keeping appointments).

  The run-in period allows staff to evaluate a patient's suitability for TODAY. At the end of the run-in, clinic staff assess the patient's adherence to study tasks, assess final eligibility criteria, and determine whether the patient should be randomized to TODAY.

- *Randomization Visit:* If the patient is eligible, final informed assent/consent is obtained and the child is randomly assigned to a treatment group at this visit. Preparations to collect detailed baseline information are scheduled.

3.2.2 Informed Consent and Assent

The TODAY informed consent process occurs in stages in order to (1) maximize potential participant and family understanding of the TODAY study; (2) allow an informed decision regarding participation, including personal risks and benefits; and (3) promote the

efficiency of study procedures. This process is designed to meet the ethical obligations to the patient and improve retention by fostering a progressively increasing understanding of TODAY by the patient and family as well as the development of a positive relationship with the clinic staff. It is an interactive, conversational process, with the ultimate goal of maximum understanding of TODAY and its impact on the family, including the responsibility of the patient to TODAY and the responsibility of the investigators to the patient. It is anticipated that one result of this process is maximized retention of participants in TODAY.

All stages in the informed consent/assent process include provision of information in verbal and written form and the opportunity for discussion and questions. Each stage allows the participant to make a decision whether to proceed to the next phase of screening. After the presentation and discussion, the participant and family member are asked to sign the assent/consent forms relevant to that stage.

3.2.3 Eligibility Criteria Prior to Run-in

Inclusion:

1. Diabetes by ADA criteria (laboratory determinations of fasting glucose ≥ 126 mg/dL, random glucose ≥ 200 mg/dL, or two-hour OGTT glucose ≥ 200 mg/dL) documented and confirmed in medical record. For asymptomatic patients diagnosed with diabetes with a normal fasting glucose but an elevated two-hour glucose during an OGTT, the HbA1c must be ≥ 6%. For patients previously diagnosed with diabetes and on medication at the time of screening for eligibility, a laboratory determination of HbA1c ≥ 8% at the time of diagnosis will be accepted as surrogate evidence of eligibility, <u>if there was no documented laboratory determination of serum glucose.</u>
2. Duration since diagnosis less than two years by date of randomization.
3. BMI ≥ 85th percentile documented at time of diagnosis <u>or</u> at screening.
4. Fasting C-peptide at screening (drawn at least one week after treatment for ketosis or acidosis, if applicable) > 0.6 ng/mL.
5. Absence of pancreatic autoimmunity (both GAD and ICA512 negative).
6. Age 10-17, with randomization prior to the 18th birthday.
7. Signed informed consent/assent forms for the pre-randomization period.
8. A family member or adult closely involved in the daily activities of the child agrees to participate in the child's treatment.
9. Fluency in English or Spanish for both child and family member.
10. Patient and family able to fully participate in trial protocol in the opinion of the investigator.

Exclusion:

11. Participating in another interventional research study protocol in the past 30 days.
12. Genetic syndrome or disorder known to affect glucose tolerance other than diabetes.
13. Patient on inhaled steroids at dose above 1000 mcg daily Flovent equivalent.
14. Patient on a course of oral steroids within the last 60 days or on oral steroids more than 20 days during the past year.
15. Patient on medication(s) that are known to affect insulin sensitivity or secretion within the last 30 days.
16. Patient on medication(s) that are known to cause weight gain within the last 30 days.
17. Patient on any weight-loss medication(s) within the last 30 days.
18. Patient on medication(s) known to affect the metabolism of study drug.
19. Inability to comprehend the lowest grade level at which lifestyle intervention materials are prepared, for both child and participating family member.

20. Females who are pregnant, planning to become pregnant within two years of enrollment, or who admit sexual activity without appropriate contraception.
21. Calculated creatinine clearance < 70 mL/min.
22. Any transaminase > 2.5 ULN. If any transaminase 1.5-2.5 times ULN, then patient must be appropriately evaluated (minimum evaluation includes ceruloplasmin level, alpha-1 antitrypsin phenotype, ANA, anti-smooth muscle antibody, anti-LKM antibody, anti-HCV, and anti-HBc total antibody not IgM, iron, and TIBC) and is eligible if all causes for transaminase elevation (other than NAFLD) are ruled out, and it is presumed that the elevation is due only to non-alcoholic fatty liver disease (NAFLD).
23. DKA at any time after diagnosis unless only a single episode of DKA related to a significant medical illness.
24. Physical limitations preventing patient from being randomized to the lifestyle intervention.
25. Patient plans to leave the geographic area within one calendar year.
26. Abnormal reticulocyte count or HbA1c chromatogram indicating the presence of abnormal hemoglobin variants other than heterozygosity for S and C at time of screening. Patients homozygous for S, C or S/C are excluded.
27. Admitted use of anabolic steroids within the past 60 days.
28. Other significant organ system illness or condition (including psychiatric or developmental disorder) that would prevent participation in the opinion of the investigator.
29. Patient participates in a formal weight-loss program.

3.2.4 Eligibility Criteria After Run-in Prior to Randomization

The list includes criteria confirming protocol adherence during run-in as well as re-confirmation of some eligibility criteria from the first screening.

Inclusion:

1. Duration since diagnosis less than 2 years at randomization.
2. HbA1c < 8% on metformin alone.
3. Age 10-17, with randomization before patient is 18 years old.
4. Signed consent/assent forms for randomization and the post-randomization phase.
5. A family member or adult closely involved in the daily activities of the child agrees to participate in the child’s treatment.
6. Fluency in English or Spanish for both child and family member.
7. Patient and family able to fully participate in trial protocol in the opinion of the investigator.

Exclusion:

8. Refractory hypertension: average systolic blood pressure ≥ 150 mmHg or average diastolic blood pressure ≥ 95 mmHg despite appropriate medical therapy.
9. Refractory hyperlipidemia: total cholesterol > 300 mg/dL or LDL > 190 mg/dL or triglycerides > 800 mg/dL, despite appropriate medical therapy.
10. Refractory anemia: hematocrit < 30% or hemoglobin < 10 gm/dL despite appropriate medical therapy.
11. Patient on a TZD within the last 12 weeks.
12. Patient on non-study diabetes medications within the past 6 weeks.
13. Patient on inhaled steroids at dose above 1000 mcg daily Flovent equivalent.
14. Patient on a course of oral steroids within the last 60 days or on oral steroids more than 20 days during the past year.

15. Patient on medication(s) that are known to affect insulin sensitivity or secretion within the last 30 days.
16. Patient on medication(s) that are known to cause weight gain within the last 30 days.
17. Patient on any weight-loss medication(s) within the last 30 days.
18. Patient on medication(s) known to affect the metabolism of study drug.
19. Inability to comprehend the lowest grade level at which lifestyle intervention materials are prepared, for both child and participating family member, assessed by mastery of standard diabetes education program administered during run-in.
20. Inability to comply with requirements of study during run-in period.
21. Females who are pregnant, planning to become pregnant within two years of enrollment, or who admit sexual activity without appropriate contraception.
22. Calculated creatinine clearance < 70 mL/min.
23. Any transaminase > 2.5 ULN. If any transaminase 1.5-2.5 times ULN, then the subject must be appropriately evaluated, if not already done (see Section 3.2.3, Exclusion #22) and is eligible for randomization if all causes for transaminase elevation (other than NAFLD) are ruled out and it is presumed that the elevation is due only to non-alcoholic fatty liver disease (NAFLD).
24. Physical limitations preventing patient from being randomized to the lifestyle intervention.
25. Patient plans to leave the geographic area within one calendar year.
26. Admitted use of anabolic steroids within 60 days.
27. Other significant organ system illness or condition (including psychiatric or developmental disorder) that would prevent participation in the opinion of the investigator.
28. Patient participates in a formal weight loss program.
29. Episode of DKA during the run-in.
30. Edema at the time of randomization (a participant who experiences edema during run-in must have recovered within 2 weeks and be edema free for 1 week prior to randomization).

3.3 Randomization

Randomization is stratified by clinical center to ensure balance among the treatment groups with respect to anticipated differences in the participant populations.

For each clinical center, the CoC generates a 1:1:1 randomization scheme using a permuted block design. Sample sizes across the three treatment arms remain relatively equivalent as the trial progresses, but the next treatment assignment cannot be anticipated.

The clinical center coordinator uses a computer-based system to input eligibility data and receive a random treatment assignment.

4 Treatment Administration and Patient Management

The goal of study treatment is to reach and maintain an HbA1c level ≤ 6%.

4.1 Pre-randomization (Run-in) Period

One component of eligibility screening is a pre-randomization run-in period of up to 6 months but no less than 2 months. The goals of the run-in are to:

- establish a relatively homogeneous study cohort with regard to therapies prior to randomization;

- determine whether potential participants can tolerate metformin within the dose range used in the study;
- ensure that glycemic control can be maintained with metformin alone in a safe range (< 8%) without ketonuria (after withdrawal of other diabetes medications in those participants who were previously treated with them);
- master the standard diabetes education program; and
- assess the ability of the potential participants and their families to adhere to the protocol.

4.1.1 Medical Management

Given that potential participants may be treated with a variety of medication regimens prior to study entry and that metformin is used in all treatment arms, all participants are required to take a minimum of 500 mg of metformin bid in order to be eligible for randomization. During the run-in, metformin naïve participants and participants who are already treated with metformin have metformin initiated and/or doses adjusted according to the recommended titration schedule (table below) with the goal of being able to be treated with 1000 mg bid without side effects and without ketosis. Since all patients are required to take two capsules (consisting of rosiglitazone, metformin, or placebo) twice daily after randomization (see section on masking), patients are required to take two capsules twice a day during the run-in period as well. During the run-in period, the patient is masked to the dose of metformin.

Participants who are already treated with metformin 1000 mg bid need not adjust their doses unless gastrointestinal or other symptoms associated with metformin occur and require dose adjustment.

Run-in Metformin Titration Schedule (for Metformin Naïve Patients)

Visit	*Pre-breakfast*		*Pre-dinner*	
	Capsule 1	*Capsule 2*	*Capsule 1*	*Capsule 2*
0	placebo	placebo	500 mg	placebo
1	500 mg	placebo	500 mg	placebo
2	500 mg	placebo	500 mg	500 mg
3	500 mg	500 mg	500 mg	500 mg

Baseline and interval HbA1c assays are performed in the central laboratory during the run-in period.

Run-in activities depend on baseline HbA1c as follows:

- If baseline HbA1c < 8% and metformin is already being used in maximal doses (1000 mg bid), no further changes are required.

- If baseline HbA1c < 8% and the participant is not treated with any medication, metformin should be started and increased according to the titration schedule above to achieve the maximum tolerated dose.

- If baseline HbA1c < 8% but the participant is treated with metformin plus other medication(s) such as a sulfonylurea, glitinide, thiazolidinedione, or insulin, metformin should be titrated to maximal tolerated doses and other medications should be tapered and discontinued as glucose levels permit. The schedule for these changes in medications is at the discretion of the investigator.

- If baseline HbA1c ≥ 8%, the subject should be placed on metformin at maximal tolerated doses and other medications should be weaned with the goal of achieving an HbA1c < 8% (checked monthly) and no ketonuria. If glycemic goals are not achieved, insulin should be added but other diabetes medications, such as thiazolidinediones, sulfonylureas, or glitinides, should not be used. Changes in medications are at the discretion of the investigator. Glucose monitoring, at least four times per day while on insulin, and monitoring for ketones should be used to guide medication adjustments and to achieve HbA1c < 8%. Once HbA1c is < 8%, insulin should be tapered and discontinued as glucose and ketone levels permit. HbA1c must be < 8% on metformin alone (at least 500 mg bid) at the time of randomization. The patient must be off other diabetes medications for at least 6 weeks, off TZD for at least 12 weeks, and have no ketonuria.

In summary, the run-in period is between 2 and 6 months. It may be as brief as 2 months if the initial HbA1c is < 8% and the participant is either on metformin alone or is started on metformin at the beginning of the run-in. Assuming that metformin (at least 500 mg bid) is tolerated, the standard diabetes education program is mastered, the adherence tasks are completed satisfactorily, and the metabolic goals (HbA1c < 8% and no ketonuria) achieved, the participant may be randomized after 2 months if the HbA1c at 2 months is also < 8%.

Participants who have had other diabetes medications tapered must be randomized by 6 months. To be eligible for randomization by 6 months, patients must be on metformin alone (at least 500 mg bid), off all other diabetes medications for at least 6 weeks, off TZD for at least 12 weeks, and have HbA1c < 8%.

Participants who are not eligible for randomization will be referred back to their primary diabetologist for resumption of on-going care.

4.1.2 Standard Diabetes Education

All patients who are screened for participation in the study receive standard diabetes education (SDE) because SDE is part of the current standard of care for T2DM and because the knowledge and skills imparted are crucial to the patient’s ability to follow treatment guidelines in each of the treatment arms.

The purpose of the standard diabetes education (SDE) program is to provide the participants and their parent(s) or guardian(s) with basic knowledge about T2DM and to teach basic survival skills and behaviors that are important for the successful management of this disease. Every patient screened for participation in the study receives the SDE program, which is distinct from the intensive lifestyle intervention that participants randomized to that treatment group receive. As has been shown by multiple studies and meta-analyses [Brown 1990, 1992; Padgett et al. 1988], education is a necessary, but not sufficient, intervention to enhance self-care in people with diabetes. Currently, there are no studies of educational programs for youth with T2DM in the literature. The SDE program is based on the extensive literature on T2DM education in adults and T1DM education in children and youth.

Program materials are provided in a workbook format that is easy to read and culturally appropriate. After each session, mastery activities are included. A participant who does not demonstrate mastery initially is provided with a different subset of questions of equivalent difficulty to assess mastery. Material can be reviewed in person, via telemedicine, or on the phone by the team certified diabetes educator (CDE) concurrently with pre-randomization

study visits. Content is based on the recommendations for nutrition, activity, blood glucose monitoring, and medications that are standard for all groups.

The education program during the pre-randomization period consists of a minimum of six sessions, each lasting 60-90 minutes. Major topics include what diabetes is, nutrition, medications, monitoring, and physical activity:

- *T2DM and its treatment:* medications (what they do and how to take them) and simple logs for keeping track of blood sugar on a twice-daily basis.
- *Weight management:* reviewing food logs and understanding how high calorie drinks and foods, especially fast foods, work against weight management. Portion control, healthier choices, and shopping for healthier foods are all discussed.
- *Physical activity:* understanding how activity helps with weight and blood sugar control, discussing potential areas for increasing activity, and understanding hypoglycemia and its relationship to activity.
- *Living with diabetes:* introduces the concept of setting achievable goals and working toward them.

4.1.3 Demonstration of Adherence

Prior to randomization, all patients also need to demonstrate the ability to adhere to the study assessments and interventions. Clinic attendance and completion of a basic set of behavioral tasks during the eligibility assessment phase demonstrate the willingness and ability of potential participants to adhere to the regimen of study assessments and interventions.

During the run-in period, while diabetes medications (including metformin) are adjusted, the clinic staff assesses the ability of the participants and their families to adhere to the protocol. Satisfactory completion of behavioral tasks, including the performance of self-glucose monitoring, medication taking, keeping a glucose diary and nutrition and activity logs, and attending scheduled visits, are examined by the clinic staff for a period of two months.

Adherence to the treatment regimen specified under the section on medical management, including self-monitoring of blood glucose, is measured. Adherence to metformin is measured by pill counts at all visits. Adherence with scheduled blood glucose monitoring is assessed by downloading the glucose meter. In addition, the patient is asked to keep nutrition and activity records during the eligibility assessment phase that are reviewed and assessed prior to randomization.

4.2 Post-randomization Period

4.2.1 Medical Management

In order to be eligible for the study, all patients must have an HbA1c < 8%, tolerate at least 500 mg and at most 1000 mg metformin bid, and take no other diabetes medications. Eligible patients are randomized to one of three treatment groups: (1) metformin alone, (2) metformin plus rosiglitazone, or (3) metformin plus intensive lifestyle intervention.

4.2.1.1 Metformin

Metformin use is the same in all treatment groups. Namely, patients remain on the maximum tolerated dose of metformin from 500 up to 1000 mg bid throughout the study.

4.2.1.2 Rosiglitazone

Patients who are randomized to metformin plus rosiglitazone treatment group are started on a dose of 2 mg rosiglitazone bid. After 8 weeks, the dose is increased to 4 mg bid. Patients who reach the primary endpoint and begin add-on insulin therapy (see section 4.7) have the rosiglitazone dose lowered to 2 mg bid.

4.2.2 Hypoglycemia

If a patient who is not receiving insulin has a severe hypoglycemic event as defined by the need to be treated with glucagon, the need for a third party to resolve a hypoglycemic episode, or loss of consciousness or seizure, then study medications are adjusted downward. If after one month there are no additional hypoglycemic events and if HbA1c values rise to > 6%, the dose of study medications is returned to the previous dose. If a second episode of severe hypoglycemia occurs, the dose is decreased until the end of the trial. If more than two severe hypoglycemic events occur in the same patient in the absence of insulin, then the patient is unmasked and study medications are adjusted downward until hypoglycemia is resolved.

4.2.3 Ongoing Standard Diabetes Education

Standard diabetes education is provided throughout the study for all three treatment groups in the post-randomization follow-up period, as follows:

- Sequenced content is provided by the study's diabetes educator at each medical visit. Content is typically provided in one-to-one sessions, but groups could be used. Sessions are brief and content for each visit is defined in the MOP.
- Additional 'need-to-know' information is provided to address specific educational concerns. Available education handouts are listed in the MOP.
- Adolescent content is taught to participants ages 12 years and older, whether during run-in or after randomization.
- Ongoing educational assessment includes assessment of psychomotor skills (meter use) at each medical visit and annual re-assessment of knowledge mastery.
- Further education and/ or assessment is provided for participants who request further information or who demonstrate poor adherence or poor metabolic control.

4.3 Masking

As noted above, during the run-in period, the patient is masked to the dose of metformin. Due to the established titration schedule for metformin and the need for dose adjustment according to side effects, staff are effectively unmasked to the dose of metformin.

After randomization, both patient and study staff are masked to medical management treatment arm.

After randomization, both patient and study staff are masked to HbA1c levels. Study staff are alerted if HbA1c ≥ 8.0%, or if HbA1c is between 6-8% with an increase between measurements ≥ 0.8%. Study staff are also alerted if HbA1c ≤ 6%.

A series of capsules containing all possible medication combinations is prepared and all types of capsules look identical. Participants take two capsules two times per day, at breakfast and at dinner. The table below presents all possible combinations of study medications.

TODAY Study Medications				
	AM		PM	
Total Daily Dose	*Capsule 1*	*Capsule 2*	*Capsule 1*	*Capsule 2*
• Run-in Period Only				
500 mg metformin	placebo	placebo	500 mg metformin	placebo
• Run-in Period, or Metformin Only Treatment Arm, or Metformin Plus TLP Treatment Arm				
1000 mg metformin	500 mg metformin	placebo	500 mg metformin	placebo
1500 mg metformin	500 mg metformin	placebo	500 mg metformin	500 mg metformin
2000 mg metformin	500 mg metformin	500 mg metformin	500 mg metformin	500 mg metformin
• Metformin Plus Rosiglitazone Treatment Arm				
1000 mg metformin + 4 mg rosiglitazone	500/2 mg avandamet	placebo	500/2 mg avandamet	placebo
1500 mg metformin + 4 mg rosiglitazone	500/2 mg avandamet	placebo	500/2 mg avandamet	500 mg metformin
2000 mg metformin + 4 mg rosiglitazone	500/2 mg avandamet	500 mg metformin	500/2 mg avandamet	500 mg metformin
1000 mg metformin + 8 mg rosiglitazone	500/4 mg avandamet	placebo	500/4 mg avandamet	placebo
1500 mg metformin + 8 mg rosiglitazone	500/4 mg avandamet	placebo	500/4 mg avandamet	500 mg metformin
2000 mg metformin + 8 mg rosiglitazone	500/4 mg avandamet	500 mg metformin	500/4 mg avandamet	500 mg metformin

4.4 TODAY Lifestyle Program (TLP)

4.4.1 Program Goals

The overall goal of the lifestyle intervention is to provide participants randomized to the metformin plus intensive lifestyle arm an evidence-supported, family-based, comprehensive weight management program that is developmentally and culturally appropriate. Sustained weight loss goals for participants are 7-10% of initial body weight. The three key components of the program are nutrition, activity, and behavior modification, and are designed to promote moderate weight loss while maintaining adequate nutrition for growth and development. These components are empirically derived and have produced the most effective obesity treatment for children studied to date [e.g., Epstein et al. 1998]. The goals of the intervention include modification of eating and activity behaviors so that new, healthier behaviors develop and replace less healthy behaviors. The approach to behavior change is positive and successive changes toward desired outcomes are modeled, practiced, and reinforced.

If the TLP is determined to be successful at treating T2DM in children and adolescents, then all children not assigned to the lifestyle intervention arm will be offered a modified version of the program.

4.4.2 Program Description

The TODAY intervention materials are based on the family-based behavioral weight

control program developed by Epstein and colleagues [Epstein et al. 1998]. This program has been studied extensively and has demonstrated efficacy in promoting weight loss that is sustained for a ten-year period. In addition, this approach has been successfully adapted for adolescents [Saelens et al. 2002]. Studies documenting the largest and longest-term decreases in percent overweight (a desirable outcome in treating T2DM) typically include parental or caregiver participation as an integral component [Faith et al. 2001; Goldfield and Epstein 2002; Epstein et al. 1994; Golan et al. 1998]. Additionally, evidence suggests that family-based approaches may be particularly well suited to ethnic minority groups [Dounchis et al. 2001]. Therefore, the program requires participation of the youth and at least one adult who is closely involved with the daily activities of the youth. Youths must agree to participate in treatment sessions and to make changes in eating and activity. The family member must agree to attend all sessions with the youth (if unable to attend, a make-up session must be scheduled) and identify another adult caregiver to transport the child when they are unable to do so. Any adult who plays a role in the youth's eating and/or activity patterns is allowed to participate in the treatment sessions.

Under the supervision of a PhD-level behaviorist, an interventionist called a PAL (Personal Activity/nutrition Leader) works with each youth toward achieving study-specified weight loss behavior change goals in a step-wise fashion using evidence-based behavioral strategies. Although the family-based treatment is standard, its implementation allows considerable flexibility. The FSP is given the opportunity to participate if they so desire, setting their own personal goals around nutrition and activity; however, participation by the FSP in setting activity goals cannot begin until the FSP's physician has signed a release form.

4.4.3 Program Components

The key components of the program are nutrition, activity, and behavior change techniques as they apply to diet and activity [Epstein et al. 1998]. Specifically, the goals of the intervention include modification of eating and physical activity behaviors so that new, healthier behaviors develop and replace unhealthy behaviors. Although it is the modification of energy balance that produces decreases in body weight, the required behavior changes necessary to sustain weight loss are complex; therefore, an emphasis on behavior change strategies is incorporated in each session. The specific behaviors targeted include: (1) decreasing calories; (2) decreasing the intake of high calorie, low nutritive value (RED) foods; (3) decreasing sedentary behaviors; and (4) increasing activity. In addition, the FSP receives information and guidance on relevant parenting principles designed to encourage and facilitate the participant's behavior change. Participation of overweight and non-overweight family members is designed to encourage and support the appropriate behaviors and restructuring of the family environment to support appropriate levels of caloric intake and physical activity [Goldfield and Epstein 2002].

4.4.3.1 Nutrition Component

The nutrition segment is designed to decrease caloric intake and increase nutrient density. The goal is to reduce body weight by a minimum of a 1 pound per week, equivalent to a change in energy balance of 500 kcals per day. Youths are taught to gradually reduce calories from baseline levels to about 1200 to 1500 kcal per day, although this goal is flexible upward dependent on initial weight. Calories are estimated using *The Fat Counter* [Natow and Heslin 1998] and the food reference guide [Epstein and Squires 1988] that are provided to all participants. If children reach a healthy weight range, they are instructed to increase their caloric intake by about 100 kcal/day in order to maintain their weight.

The Updated Traffic Light Diet serves as the basis for the nutrition management component of the intervention. In this approach, foods are divided into the colors of the traffic light. RED foods (*stop*) are high in fat or simple carbohydrates (e.g., soft drink) and low in nutrient density. YELLOW foods (*approach with caution*) are the staples of the diet and supply basic nutrition, but should only be consumed in moderate amounts. GREEN foods (*go*) are those that are nutrient dense (e.g., fruits, vegetables). Free foods include low-calorie foods (e.g., diet soda) that participants can use to substitute for higher calorie foods. Portion sizes for some foods have been adjusted to be consistent with ADA dietary guidelines. Participants are encouraged to decrease the number of servings of RED foods. In *The Traffic Light Diet,* families are encouraged to choose healthy foods based on individual, family, and cultural preferences from lists of foods commonly available, thus allowing participants freedom of choice and encouraging greater adherence than a prescribed diet in which food choice is more limited.

4.4.3.2 Physical Activity Component

The major objective of the physical activity portion of the TODAY lifestyle intervention is to significantly increase each youth's physical activity level above baseline levels. The program is designed to be flexible with a two-tiered goal. The study goal (silver medal level) for participants is 200 minutes per week of moderate-vigorous intensity activity. The gold medal level of 300 minutes per week is available for those participants who can handle the extra challenge later in the intervention or the few whose initial activity levels are close to 200 minutes per week at baseline. Each youth is asked to spread out his or her activity minutes over the course of a week (a minimum of 3 times per week) to maximize both safety and effectiveness.

Participants' weekly activity goals gradually increase until reaching their physical activity goal. Strategies for increasing lifestyle activities (e.g., taking the stairs, walking to school) and increasing involvement in active pastimes while decreasing sedentary behavior (e.g., TV watching and playing computer games) are strongly emphasized. Activities that last at least 10 minutes in duration and are minimally the same intensity as a brisk walk count towards their weekly goals. Otherwise, they are considered lifestyle activities.

The initial phase of the activity intervention involves the use of a simple, inexpensive, objective measure of activity, the pedometer. A pedometer is a means of providing structure to the child and a way to objectively measure changes in both activity and sedentary behavior. The pedometer provides immediate feedback to the child regarding the gain in steps obtained during "highly active" activities relative to sedentary activities such as TV watching.

Each youth is also asked to keep a weekly activity log documenting his/her physical activity minutes. The total number of minutes per week determines adherence to the intervention, allowing more flexibility for the participant's schedule.

4.4.3.3 Behavioral Component

This intervention utilizes behavior therapy. Behavior therapy is based on the assumption that behavior is controlled by its antecedents and consequences, and if the cues that precede and the consequences that follow behavior are changed then the target behavior will be altered. Behavior therapy is characterized by a focus on a careful analysis of behavior and by nonjudgmental, positive attitudes, persistence, and flexibility on the part of interventionists. Core behavioral strategies include self-monitoring, stimulus control, social assertion, goal setting, feedback, and relapse prevention. Supplementary behavioral strategies include discussion of emotional eating, coping with teasing, and body image.

Additional behavioral skills for parents include the appropriate use of praise, positive reinforcement techniques, and modeling. Parents are encouraged to restructure the home environment by implementing changes using a systemic, family-wide approach (i.e., the child with diabetes should not be made to feel ‘singled out’). For parents of younger children (ages 12 and under), interventionists encourage and facilitate greater levels of direct parental involvement in treatment. Parents are encouraged to help with monitoring, to provide praise and reinforcement, and to make environmental changes both inside and outside the home (e.g., not taking their children to fast food restaurants). Interventionists encourage parents of older adolescents to actively praise and provide reinforcement for positive behavioral outcomes (when appropriate), but also to take on a more collaborative role with their children. Parents are encouraged to actively model healthy behavior (i.e., healthy eating and physical activity) and to work with the adolescent to develop a healthy home environment. Parents must also take responsibility for modifying the shared family environment in a way that supports the youth’s changes, as well as establishing and working on goals consistent with improving the family environment. A willingness to support the youth in the completion of the program and in learning skills to facilitate long-term changes is essential.

4.4.4 Treatment Structure

The intensive lifestyle intervention includes three phases: a lifestyle change (LC) phase of weekly sessions for months 1-6, followed by a bi-weekly lifestyle maintenance (LM) phase through months 7-12, and a continued contact (CC) phase from months 13 through the end of the study. The CC phase sessions are scheduled monthly for the initial 12 months (study months 13-24) and then quarterly or 4 times a year to the end of the study. During the LM and CC phases, telephone calls are held between visits. Limited intensification may occur in LM and CC phases when needed due to failure to make or maintain progress toward target goals.

The TLP is primarily an individual-based rather than group-based model of treatment because of the range of developmental stages of the participants as well as the anticipated geographic distances between participants and the clinics. Opportunities for group interactions, such as group walks and special events, may be scheduled over the entire duration of the trial to provide opportunities to incorporate the benefits of group involvement.

The format and structure of weekly family sessions remains the same during all phases of the lifestyle intervention. Each session includes reviewing short- and long-term goals, developing strategies to achieve these goals, praising successes, and problem solving in areas of difficulty. Each session includes a combination of separate and concurrent sessions by the youth and the parents. This format allows individual time for the therapist to address important developmental issues with the adolescent and provide appropriate parenting strategies/interventions for the parent. During “off periods” when not in session with the therapist, the participant completes behavior change related educational activities.

Sessions are audiotaped and some tapes are reviewed by the PAL’s Ph.D. level behaviorist and the Lifestyle Resource Core.

4.4.5 Resource Needs

The educational materials developed for the weight loss intervention are produced by the Lifestyle Materials Core (LMC) and approved by the Lifestyle Intervention Committee. Materials are developed using a mastery-based model used in education and are readable and appropriate for all ethnic groups in the trial. The PAL and the staff Ph.D. level behaviorist participate in the training program developed by the LMC. Periodic training and

monitoring sessions are planned over the course of the trial. There are weekly supervisory conference calls between LMC staff and interventionists at each clinical center during which clinical progress is discussed, problems identified, and solutions generated. Audiotaped sessions may be reviewed by the LMC for quality control purposes.

4.5 Patient Reinforcement

4.5.1 Behaviors Targeted for Reinforcement of Medical Management

Following randomization, participants in each of the three study arms receive incentive ‘points’ at every study visit contingent on having successfully adhered to two targeted behaviors: (1) medication taking and (2) blood glucose monitoring. The study diabetes educator awards study participants the points, which can be redeemed for rewards provided by the clinical center.

4.5.2 Behaviors Targeted for Reinforcement of Intensive Lifestyle Intervention

Four behaviors are targeted to be reinforced for participants in the intensive lifestyle intervention: (1) increasing physical activity, (2) decreasing sedentary behaviors, (3) reducing calories, and (4) reducing high-fat and high simple carbohydrate, high-energy density foods (RED) foods. Earning incentives for these behaviors is contingent upon weight loss and contingent on material completion. Since these behaviors are self-reported, an objective criterion such as weight loss is needed to reduce providing incentives for youth who are reporting improvements in behavior but who have not made behavior changes. Participants earn points for meeting behavior goals related to the aforementioned target behaviors.

In addition, participants earn one point for their parent and child meetings, which is not contingent upon weight loss. In the TLP arm, the PAL awards points for lifestyle adherence which can be used by the participant towards family-based incentives (e.g., special privileges and activities for the youth) awarded by the parent.

4.5.3 Implementation of the Incentive System

In all groups, a ‘reinforcement menu’ is negotiated between the parent and youth so that all the reinforcers that are provided are acceptable. Point values are associated with each available reinforcer. The participant chooses either to spend points immediately after earning them or save them for larger incentives.

4.6 Adjunct Care

In order to standardize treatment of comorbid conditions and to avoid the possibility of bias, treatment of dyslipidemia, hypertension, and microalbuminuria is centralized and follows specified treatment algorithms. A central study medical consultant provides treatment recommendations for individual patients. The study medical consultant is blinded to treatment assignment and reviews lipid, blood pressure, and urine results to assure treatment goals are met according to the treatment algorithms for each of the comorbidities. Regular review of treatment for comorbid conditions and patient outcomes is provided by the Protocol Review Committee.

4.6.1 Treatment of Dyslipidemia

Target goals of therapy are LDL cholesterol < 100 mg/dL and TG < 150 mg/dL. If baseline lipid levels are outside the target range in patients who are not receiving pharmacological therapy for dyslipidemia, initial therapy involves dietary counseling.

If LDL values remain over 130 mg/dL or if TG levels remain over 300 mg/dL after six months of nutrition and diabetes management, pharmacological treatment is initiated and adjusted to achieve target goals according to an algorithm based on lipid levels.

Patients who are being treated with statins have dosage adjusted to achieve target goals according to the algorithm based on lipid levels.

4.6.2 Treatment of Hypertension

While in the study, the target average systolic and diastolic BP is < 90th percentile for age, sex, and height [NHLBI 1996].

High-normal blood pressure is defined as an average systolic or diastolic blood pressure ≥ the 90th percentile and < the 95th percentile for age, gender, and height measured on at least two consecutive study visits and one interim visit. Therapy for high-normal blood pressure includes dietary intervention consisting of elimination of added salt to cooked foods and a reduction in foods high in sodium content.

Hypertension is defined as an average systolic or diastolic blood pressure ≥ the 95th percentile for age, gender, and height measured on at least two consecutive study visits and one interim visit. Diagnostic tests include a routine urinalysis, blood urea nitrogen, and serum creatinine to screen for renal related disease. Femoral pulses should be palpated and the blood pressure of both legs measured to exclude coarctation of the aorta. Run-in management includes monitoring and treatment of hypertension.

Subjects with hypertension are placed on dietary intervention consisting of elimination of added salt to cooked foods and a reduction in foods high in sodium content. In addition, initial pharmacological treatment of hypertension consists of a single ACE inhibitor with dose titrated to achieve target blood pressure. If target is not reached, additional medications may be added at the discretion of the study physician, in consultation with the study central medical consultant.

Patients who develop hypertension while in the study are treated first with dietary sodium restriction as above and then with an ACE inhibitor, then with additional medications as above as needed to achieve target blood pressure in consultation with the central medical consultant as needed.

Patients already being treated with anti-hypertensive agents should remain on such therapy, adjusted as needed to attain treatment goal. Copies of previous medical records are obtained to classify the patient as having high-normal blood pressure or hypertension pretreatment. Patients whose blood pressure remains elevated (systolic ≥ 150 mmHg or diastolic ≥ 95 mmHg) on anti-hypertensive therapy may not be randomized.

Treatment of microalbuminuria with an ACE inhibitor is initiated at the time of diagnosis, regardless of blood pressure.

4.7 Add-on Insulin Therapy

- *Failure to maintain adequate metabolic control:* If HbA1c ≥ 8.0%, therapy is re-invigorated by more frequent telephone contact and visits. If HbA1c remains ≥ 8% throughout the next six months, then the patient is classified as failing to maintain

adequate metabolic control and if the participant's fasting blood glucose values are not in target then add-on therapy with insulin is initiated.

- *Outcome assessments before the initiation of add-on insulin therapy:* Before starting insulin treatment, participants undergo a comprehensive outcomes assessment unless this has been completed within the past three months.

- *Add-on insulin treatment regimen:* Patients who meet criteria for add-on insulin therapy continue to take the study drug metformin but discontinue the study drug rosiglitazone. The patients and clinicians remain masked to the treatment assignment but are unmasked to HbA1c. Initial insulin treatment is glargine insulin, 0.2 units per kilogram given in the evening. The dose is increased up to 1.0 U/kg/day (to a maximum of 100 units), until fasting blood glucose (FBG) values between 70-150 mg/dL are achieved.

  Add-on glargine insulin therapy is considered unsuccessful if (1) 1.0 U/kg/day (to a maximum of 100 units) does not bring FBG to target within 1 month or (2) HbA1c > 8% at 3 months or (3) HbA1c > 7% at 6 months. At that point, insulin therapy—including adding rapid, short, or intermediate acting insulin—is provided at the clinician's discretion.

- *Self-monitoring of blood glucose (SMBG):* During insulin therapy, patients are asked to monitor blood glucose levels at least four times a day. Target fasting glucose and pre-meal glucose is 70-150 mg/dL and the target peak postprandial glucose is ≤ 200 mg/dL.

- *Temporary use of insulin:* Some patients may require temporary suspension of study drug and use of insulin. This is referred to as 'on leave' and may be due to (1) temporary medical conditions such as hospitalization or intercurrent illness (see chapter on safety), (2) a sexually active female not on appropriate contraception, or (3) pregnancy (see section on pregnancy below). While 'on leave', patients and clinicians remain masked to study drug and TLP activities are modified as needed by the intervention team. The patient continues to attend quarterly visits but HbA1c outcome measurement is discontinued during temporary use of insulin, except during pregnancy and nursing when A1c is measured for clinical care. Any case in which the participant has been on temporary insulin therapy for ≥ 2 weeks must be reviewed by the Protocol Review Committee. If the participant has been off study medications for ≥ 1 month, study procedures associated with an annual visit are not collected until the patient is back on study treatment (drug and/or TLP) for an adequate time period.

  Any type or dose of insulin can be used at the discretion of the treatment team. In such cases, an attempt is made to withdraw insulin once the acute event has resolved and study drugs are resumed. In the case of pregnancy, participants are weaned over a 3 month period once pregnancy and lactation are complete. In the case of a temporary medical condition such as hospitalization or intercurrent illness, weaning occurs over 2 weeks if the event lasted 2 weeks or less; if the event lasted more than 2 weeks, weaning occurs over 1 month. Withdrawal of insulin occurs regardless of blood glucose values; if metabolic decompensation occurs, appropriate safety procedures are followed (see next bullet for details).

- *Metabolic decompensation:* Metabolic decompensation is defined as hyperglycemia (BG > 300 mg/dL) accompanied by significant symptoms (e.g., vomiting, dehydration, lethargy) and/or moderate or large urinary ketones, or sustained hyperglycemia during home glucose monitoring [80% of BG tests are > 300 mg/dL (non-fasting) or > 200

mg/dL (fasting) for 1 week]. When this occurs, the participant should be evaluated to determine if temporary use of insulin therapy is required. All patients are educated to contact the study coordinator if they experience metabolic decompensation. This education is reinforced at study visits. Inability to wean the participant within three months, without urinary ketones (moderate or large) or significant symptoms of hyperglycemia, results in classification as a treatment failure.

4.8 Pregnancy and Sexual Activity

Female participants of childbearing age who meet other eligibility requirements and wish to participate in the study are informed of the potential risks to a pregnancy conceived while on any study pharmacological treatment. Such participants are also informed of the potential risks of hyperglycemia to a pregnancy including fetal malformations, pre-term delivery, C-section, and the potential increased risk for maternal progression of renal disease. Participants are informed of the potential for rosiglitazone and metformin to enhance fertility. Those who consent to participate are asked to practice reliable birth control including systemic hormones and/or barrier methods. Patients who are pregnant and/or are sexually active and not using adequate birth control are excluded from enrollment in the trial.

- *Safety monitoring:* Pregnancy tests are obtained from all female participants of childbearing age at each visit and right before DXA scans. Participants are asked to obtain pregnancy tests if pregnancy is suspected. The diagnosis of pregnancy can be made for the study purposes by a positive urine pregnancy test in a patient who has missed one or more periods.

  Sexually active female patients (i.e., admit to having sexual relations) who are not using adequate birth control (i.e., taking contraceptive medications or reliably using barrier method birth control) are ineligible for the study. If an enrolled participant is known to be sexually active without the use of appropriate contraception, the patient is placed ‘on leave’ from the study as described above.

- *Planned pregnancy:* A patient who wishes to become pregnant is advised to come in for pre-pregnancy counseling. Study medications are stopped, and the patient is considered ‘on leave’ from the study while attempting to become pregnant, during pregnancy, or while lactating. Data on pregnancy outcome are collected for safety evaluation and to address the secondary outcome.

- *Unplanned pregnancy:* A patient who has become pregnant is referred to a high-risk obstetrical team with primary responsibility for the management of blood glucose levels. The patient is considered ‘on leave’ from the study while pregnant or while lactating. Neither metformin nor rosiglitazone are indicated in pregnancy. A participant found to be pregnant while taking coded medication has her coded medication discontinued and is immediately unmasked to the pharmacological treatment assignment. Information on the potential teratogenicity of metformin and rosiglitazone is provided to both the participant as well as her provider(s) of obstetrical care. Data on pregnancy outcome are collected for safety evaluation and to address the secondary outcome. The patient may return to the study protocol following delivery and/or lactation.

- *Visits:* Subjects are followed every 3 months during pregnancy and/or lactation. Collection of most study visit outcome data, with the exception of weight and HbA1c for

safety purposes, is suspended during pregnancy and lactation. The course of the pregnancy is monitored but much of the pregnancy-related data are extracted from medical records.

- *Interventions post-pregnancy and breastfeeding:* Study interventions, including the intensive lifestyle intervention, are suspended for the duration of breastfeeding.
  Assessment of the ongoing need for insulin begins in the hospital immediately post-partum. Women discharged on insulin are evaluated with home glucose monitoring to determine the ongoing need for insulin.
  After pregnancy and lactation, attempts are made to withdraw insulin and patients are restarted on their previous study medication. Participants are allowed three months after delivery and lactation to discontinue insulin, and are considered a treatment failure if unable to discontinue insulin therapy without metabolic decompensation during this time period.

5 Research Procedures and Approach

5.1 Data Collection

The table lists the schedule of data collection, measurements, and assessments.

**Data Collection**					
**Measurement/ Assessment**	**Eligibility**		**Baseline**	**Year 1** X = every 2 months	**Post Year 1 Follow-up** Q = quarterly;A = annual 24 = 24 months P = primary outcome E = end of study
	**Initial Screen**	**Run-in** L=last run-in only			
Historical data (a)	X		X		
HbA1c	X	X	(p)	X	Q, A, 24, P, E
Blood for storage			X	6,12	A, 24, P, E
Blood for DNA				(q)	(q)
Urine for storage			X	12	A, 24, P, E
Insulin sensitivity and secretion (b)			X	6, 12	A, 24, P, E
2-hour OGTT			X	6	A, 24, P, E
Fingerstick BG (c)	X		X	X	Q, A, 24, P, E
Pancreatic autoimmunity	X				P, E
HbA1c chromatogram	X				
Reticulocyte count	X				
Serum creatinine (d)	X	L		12	A, 24, P, E
LFTs (e)	X	L		X	Q, A, 24, P, E
Hemoglobin, hematocrit	X	L		2, 6, 12	A, 24, P, E
Height, weight	X	X	X	X	Q, A, 24, P, E
Other anthropometrics (f)			X	6	24, P, E
DXA			X	6	24, P, E
Blood pressure	X	X	X	X	Q, A, 24, P, E
Lipids	X (g)		X	6,12	A, 24, P, E
Physical exam (h)	X		X	X	Q, A, 24, P, E
Pregnancy and sexual activity evaluation	X	X	X	X	Q, A, 24, P, E
Diabetes management	X	X	X	X	Q, A, 24, P, E
Diabetes complications			X	X	Q, A, 24, P, E
Concomitant medications			X	X	Q, A, 24, P, E
Interim history				X	Q, A, 24, P, E
Fitness, nutrition, activity (i)			X	6	24, P, E
Psychosocial and QoL (j)			X	6	24, P, E

**Data Collection**					
**Measurement/ Assessment**	**Eligibility**		**Baseline**	**Year 1**	**Post Year 1 Follow-up**
	**Initial Screen**	**Run-in** L=last run-in only		X = every 2 months	Q = quarterly;A = annual 24 = 24 months P = primary outcome E = end of study
Cardiovascular risk factors (k)			X	6,12	A, 24, P, E
Peripheral neuropathy (MNSI)			X	12	A, 24, P, E
Microalbuminuria			X	12	A, 24, P, E
BGM download		X	X	X	Q, A, 24, P, E
Standard diabetes education		X			
Medication dose		X	X	X	Q, A, 24, P, E
Protocol adherence		X	X	X	Q, A, 24, P, E
Adverse events (l)		X	X	X	Q, A, 24, P, E
Treatment group assignment			X		
Biological parent (m)			X		
FSP height			X		
FSP weight			X	12	A, 24, P, E
FSP surveys (n)			X		24, P
Resource utilization costs (o)			X	X	Q, A, 24, P, E
Retinopathy screening					Final year of study
Echocardiogram					Final year of study

(a) Historical data include family and medical history, births and pregnancies, feeding, and demographics (including socioeconomic status).
(b) Insulin sensitivity and secretion measures include fasting glucose, insulin, C-peptide, and proinsulin. HOMA and QUICKI are computed.
(c) Urine ketones are measured as indicated if BG > 300 mg/dL.
(d) Serum creatinine is used to calculate creatinine clearance.
(e) If transaminases > 1.5 ULN, safety protocol is followed.
(f) Other anthropometric measurements are waist circumference and abdominal height.
(g) Screening lipid values are LDL and TG for determining eligibility. Other lipid assays include free fatty acids, lipoprotein subclass levels, average LDL particle density, and total ApoB levels.
(h) A comprehensive physical exam including Tanner stage and evaluation of acanthosis nigricans is performed at screening, baseline, all annual visits, and outcome. Otherwise a targeted physical exam is performed (every 2 months in year 1 follow-up and then quarterly).
(i) The FFQ is used for nutrition, 3-day PDPAR and 7-day recorded accelerometer for physical activity, and PWC 170 for fitness.
(j) The psychosocial and quality of life battery includes CDI, BDI, EDEQ (and QEWP-R if participant has high score on the EDEQ), PEDS QL, and HUI-2.
(k) Cardiovascular risk factors include measurement of fibrinogen, c-reactive protein, homocysteine (vitamin B-12 will be obtained to assess homocysteine), plasminogen activator inhibitor-1, interleukin-6. Pro-inflammatory and hemostasis markers are assayed at baseline, 6 months, 12 months, and end of study; blood from other draws is stored.
(l) Participants are asked about adverse events (AE) at clinic visits, but AE and SAE (serious adverse events) may be reported by the patient at any time.
(m) Data collected from the biological parent at baseline are height, weight, BDI, and EDEQ (and QEWP-R depending on EDEQ high score).
(n) Survey data include BDI, EDEQ (and QEWP-R depending on EDEQ high score), PEDS QL (adult proxy) and CHQ (P28).
(o) Data are collected every 2 months during the first year and quarterly thereafter from clinic staff on time spent for patient treatment. Logbook data are collected from clinic

staff every four months to further assess time spent providing treatment. Data are collected from patients and families regarding time and resource use related to treatment at baseline, 6 months, annually, primary endpoint, and at study closeout.

(p) The last HbA1c value during run-in is considered the baseline HbA1c.

(q) A sample of blood for DNA may be collected any one time after randomization.

5.2 Outcomes Assessment Pilot

Before randomization and enrollment of participants, each clinical center completes a pilot study of procedures and activities involved in the major outcomes assessment visits (i.e., baseline, 6 month, 24 month, primary endpoint, and end of study). These assessments include (see data collection table in section 5.1):

1. the battery of questionnaires and surveys covering diet, activity, psychosocial factors, and medical history;
2. comprehensive physical examination;
3. blood and urine tests, including OGTT, HbA1c, lipids, liver function, etc.;
4. DXA;
5. PWC170.

The purpose of the pilot study is to provide information regarding:

- the logistics and environment for performing these procedures and activities,
- family and child reaction, and
- transfer of specimens and data among the clinical centers, coordinating center, central labs, and reading centers.

Children who participate in the pilot study meet the same inclusion and exclusion criteria as the study participants, but would not be eligible due to child's age (> 17 years) or duration of diabetes (> 2 years). The parents and child decide which of the five procedures and activities listed above they will agree to complete, and indicate by initialing those procedures and activities on the consent/assent forms. Each clinical center recruits enough children so that two children complete each procedure or activity. Children are paid $25 for each of the five procedures/activities performed. Parents receive reimbursement for costs incurred to attend clinic visits, as for the full-scale trial.

5.3 Participant Retention Program

Retention refers to efforts to prevent participant dropout or withdrawal from the study. It is critically important to successfully engage and retain participation over the course of the trial. For purposes of sample size estimation, investigators have predicted withdrawal rates of 10% over each 6 month follow-up period. However, lower rates of attrition are desirable.

Challenges to retention include:

- *Burden* (e.g., the imposition caused by study procedures, frequent scheduling of procedures and study-related visits, the requirement for record keeping, the necessity of frequent glucose monitoring, the interference of study activities with other things the participant would like to do)

- *Logistics* (e.g., travel required for study participation, care of non-participating children while the parent is at a study visit, difficulty with dietary requirements such as limited access to fresh fruits and vegetables)

- *Environment* (e.g., lack of supportive individuals and institutions, deficits due to locale such as the lack of a grocery store or a safe place to be active)

- *Education* (e.g., low literacy, familial misinformation about diabetes, sociocultural mismatch between family and educator, language barriers)

- *Distrust* (e.g., the perception by the participant and/or family of being used as a 'guinea pig,' general suspicion of medical personnel or organizations, wariness of people of different ethnicity or race.

Specific strategies to systematically address each of these areas are developed. Activities at both the national and local level are utilized and coordinated to maximize retention and minimize attrition. For example, in response to the perception that "the study team is more interested in the study than the individual," the TODAY study maintains a database of ideas about positive approaches to participants. At the local level, study staff make a major effort to personalize the study (mailing out personal notes, birthday cards, etc). Individual sites develop strategies to enhance retention specific to locale (i.e., different strategies may be required in Oklahoma working with rural Native Americans as compared to working with urban African Americans in Philadelphia).

Attrition is monitored regularly by the coordinating center and the Recruitment and Retention Committee. An attempt is made to collect data on the reason for leaving the study in the case of a participant who withdraws. Assistance is offered to any site with a higher than average attrition rate. Sites are also encouraged to share their ideas and experiences via regular communication and conference calls for project and recruitment coordinators.

5.4 Confidentiality

The study complies with HIPAA guidelines regarding confidentiality of patient data.

Patients who participate in the 1-6 month run-in period are assigned a study identification number:

- the first three digits indicate the clinical center and
- the next four digits are individually assigned by patient at each clinical center (0001-9999).

In addition, each patient randomized is associated with an acrostic or 'handle' of up to 6 alpha-numeric characters that is selected by the clinical center coordinator according to the following guidelines:

- neutral, i.e., not offensive, and
- unrelated to personal characteristics or identifiers, e.g., no initials or nicknames.

The purpose of the acrostic is two-fold. First, it acts as a check and back-up of the study ID number in case of transcription or entry error. Second, it facilitates coordinator recall of a specific patient.

All data are labeled with the study ID, including forms and specimens. All data transferred to the CoC for accumulation in the central database identify the patient only with the study ID and acrostic. The CoC does not receive any personal identifiers.

Each clinical center maintains a file on each patient that includes personal identifiers, linking name and contact information to the study ID. These data are not entered into the study data management system or into any file on the study-dedicated computer supplied by the CoC. Patient files are kept in secure locations and the clinical center is responsible for

taking every other reasonable measure (those set by the state, the site, and the study) to ensure and maintain record confidentiality and patient privacy.

Training sessions cover confidentiality principles and procedures.

6 Safety and Monitoring

6.1 Data Safety Monitoring Board (DSMB)

A Data Safety Monitoring Board consisting of appropriately qualified independent experts is appointed to provide review of data on patient safety. The purpose of the board is to assure independent review as to whether study patients are exposed to unreasonable risk because of study participation, and to monitor study progress and integrity. Board members are chosen by NIDDK in consultation with the study investigators, and a report format and reporting frequency are developed before the start of data collection. The study chair and the CoC provide periodic reports on adverse events to the committee, including summary tabulations and narrative summaries on individual events. The contents of reports are determined by the DSMB in its initial deliberations, including whether and when to perform interim efficacy analyses.

The purpose of safety reports is to present the Data Safety Monitoring Board with information regarding adverse events experienced by study patients as a result of undergoing the study procedures. Clinical centers report adverse events to the CoC in a timely fashion, including a narrative summary of the event as well as indication of the duration, perceived relationship to the study procedures, and resolution. The CoC summarizes and reports adverse events to the Data Safety Monitoring Board on a semi-annual basis unless severe or unexpected adverse events occur. These are reported promptly to the DSMB.

Following each DSMB meeting a summary of adverse events and DSMB recommendations is provided to the IRB of each participating clinical center and other institutional monitoring committees/boards as needed.

6.2 Safety Monitoring and Risk Management

If study medications are permanently discontinued for safety reasons, insulin therapy is not initiated unless the participant meets criteria for treatment failure or metabolic decompensation (see definition in section 4.7).

6.2.1 Depression

The child, biological parent, and designated family support person complete standard depression inventories. If severe depression and/or suicidal ideation are detected either from the standard surveys or through interpersonal contact between participant and study staff, the participant is referred for care outside the study.

6.2.2 Laboratory Monitoring

The purpose of periodic laboratory monitoring is to identify changes in health status that increase the risks associated with study medication (metformin and/or TZD), and to identify changes in health status as a result of participation in the trial.

After randomization, all study participants have blood glucose, SGPT/ALT, and SGOT/AST determined at every visit (every two months for the first year followed by every three months thereafter). Calculated creatinine clearance is determined at baseline and

annually. Urine ketones are measured if BG > 300 mg/dL at any clinic visit. Hemoglobin and hematocrit are measured at baseline, 2 and 6 months, and annually.

6.2.3 Potential Risks

Known adverse effects associated with metformin are primarily gastrointestinal (diarrhea, nausea, vomiting, abdominal bloating, flatulence, anorexia), hematologic (reduced vitamin B12 levels and, rarely, megaloblastic anemia), and the rare possibility of lactic acidosis. The risk of lactic acidosis associated with metformin use can be minimized by (1) monitoring liver transaminases, (2) monitoring renal function, and (3) temporary discontinuation of metformin before radiologic studies involving the injection of contrast dye, surgical procedures requiring reduced fluid intake, and serious illness that might be associated with hypoxia, dehydration, or shock.

Known adverse effects associated with rosiglitazone are primarily hematologic (decline in hemoglobin and hematocrit) and fluid retention and edema. Although hepatic toxicity has been observed with troglitazone (Rezulin®), which is no longer commercially available for human use, similar toxicity has not been observed so far with the other TZDs (rosiglitazone or pioglitizone). However, regular monitoring of transaminases is performed.

Potential risks of the lifestyle intervention are minor and include temporary muscle soreness and injuries associated with physical activity and slight hunger when dieting.

6.2.4 Procedures to Minimize Risks

- *Anemia* may be an adverse effect of either metformin or rosiglitazone. Anemia is defined as a hematocrit < 30.0%, a hemoglobin < 10 gm/dL, a decline in hematocrit by 4% from study entry, or a decline in hemoglobin by 2 gm/dL from study entry. If anemia is detected, a CBC with differential is obtained within one month. If anemia is confirmed, determination of a vitamin B12 level, examination of the blood smear, and other tests (as indicated) are performed at the discretion of the primary or study physician to help determine the etiology. Vitamin B12 and/or iron supplementation can be administered as clinically indicated. If anemia persists for more than six months despite appropriate therapy, consideration is given to discontinuing study medication.

- *Renal insufficiency* increases the risk of lactic acidosis associated with metformin. Serum creatinine is determined at baseline and annually. Using the serum creatinine, a creatinine clearance is calculated. If the calculated creatinine clearance is < 70 mL/min, study medication is discontinued for two weeks and the calculated creatinine clearance is repeated. If necessary, insulin may be temporarily used during this time. If the repeat calculated creatinine clearance is normal (≥ 70 mL/min), study medication is resumed at the previous dose. If the repeat calculated creatinine clearance is again abnormal (< 70 mL/min), or if once study medication is resumed the creatinine clearance again falls to < 70 mL/min, study medication is permanently discontinued.

- *Liver complications* increase the risk of metformin-associated lactic acidosis, and drugs of the thiazolidinedione class (but not rosiglitazone or pioglitazone) have been associated with such complications. Study participants are monitored with an SGPT/ALT and an SGOT/AST every two months for the first year and every three months thereafter.

A. If either ALT or AST level rises to 1.5-2.5 times the ULN after having been <1.5 times the ULN at baseline:
   1. Study medication is continued.
   2. Repeat ALT and AST are obtained within two weeks and blood is obtained for possible hepatitis titers that will be run if levels are still elevated. Also, if the levels remain elevated after two weeks, an evaluation must be done (minimum evaluation includes ceruloplasmin level, alpha-1 antitrypsin phenotype, ANA, anti-smooth muscle antibody, anti-LKM antibody, anti-HCV, and anti-HBc total antibody not IgM, iron, and TIBC) to rule out other liver disease (other than NAFLD). If previous evaluation had been done and was normal, then the ceruloplasmin level, Iron, TIBC, and alpha-1-antitrypsin phenotype need not be repeated. All evaluations performed because of elevated ALT and/or AST should be reviewed locally by the PI and also sent to the Safety & Montitoring Committee (SMC) for their review and consultation with a panel of GI consultants, as needed.
   3. If the ALT and AST repeated at two weeks return to < 1.5 times the ULN, resume study medication and transaminase monitoring every 2 months in follow-up year 1 and every 3 months thereafter.
   4. If ALT or AST are still 1.5-2.5 times the ULN after two weeks, hepatitis titers are negative and if all causes for transaminase elevation (other than NAFLD) are ruled out and it is presumed that the elevation is due only to non-alcoholic fatty liver disease (NAFLD), study medication is continued.
   5. While on study medication, ALT and AST should be repeated monthly for 6 months to make sure the levels are not rising. If ALT and AST decrease (to < 1.5 times the ULN) or remain stable (still 1.5-2.5 times the ULN) at 6 months, routine monitoring resumes as dictated by the protocol (every 2 months in follow-up year 1 and every 3 months thereafter).
   6. If either AST or ALT rises to > 2.5 times the ULN, then the procedures listed below in B.1-B.9 are followed.

B. If either ALT or AST or both are > 2.5 times the ULN:
   1. Study medication is stopped immediately.
   2. Repeat ALT and AST in two weeks; blood is obtained for potential hepatitis titers that will be run if levels are still elevated.
   3. If levels remain elevated (ALT and/or AST >1.5 times the ULN) after two weeks, the patient must be appropriately evaluated (see A.2 above) for liver disease other than NAFLD.
   4. If the ALT and AST repeated at two weeks return to < 1.5 times the ULN, resume study medication and transaminase monitoring every 2 months in follow-up year 1 and every 3 months thereafter.
   5. If the ALT and AST repeated at two weeks decrease to 1.5-2.5 times the ULN, resume study medication and continue monitoring as in A.5 above.
   6. If the ALT or AST repeated at two weeks is > 2.5 times the ULN, the subject should remain off study medication (metformin and/or rosiglitazone) for as long as ALT and/or AST are > 2.5 times ULN. Monitoring should occur as clinically indicated, but not less frequently than every 2 months in follow-up year 1 and every 3 months thereafter.
   7. If AST and ALT subsequently return to <2.5 times the ULN and the transaminase elevation was identified as due to a specific transient and reversible etiology, study medication can be resumed.

8. If AST and ALT return to < 2.5 times ULN and no specific transient and reversible cause for the elevation was identified, rosiglitazone should be permanently discontinued, but metformin should be resumed. Monitoring should be performed as in A.5 above.
9. After a second episode of AST and/or ALT rising to > 2.5 times ULN without a specific transient and reversible cause being identified, both rosiglitazone and metformin are permanently discontinued. Continued monitoring should be performed as clinically indicated.

Participants and their families are instructed that in the event they develop malaise, vomiting, dark urine, jaundice, or right upper quadrant abdominal discomfort, they should stop study medication and contact the study clinical center immediately. Upon notification, the center staff must obtain blood for ALT and AST as soon as possible and within one week. Based on these results, the algorithm described above is followed.

- *Gastrointestinal (GI) symptoms* are a common occurrence with metformin. However, these symptoms more commonly occur early in the course of treatment. Since all eligible participants are placed on the maximal tolerated metformin dose (between 500 mg bid and 1000 mg bid) during the run-in period, few additional gastrointestinal side effects are anticipated in these patients. If GI side effects develop and are mild, the patient is encouraged to remain on the study medication. If GI side effects are moderate or difficult to tolerate, metformin (but not rosiglitazone) is reduced to the next lowest dose (for example, 1000 mg bid to 1000 mg + 500 mg; 1000 mg + 500 mg to 500 mg bid; etc.). If symptoms persist, metformin is reduced to the next step. If GI symptoms resolve, metformin is re-escalated by 500 mg per day each week until reaching the previously tolerated dose. If symptoms persist on a dose of metformin that is only 500 mg bid, study medication is discontinued.

- *Edema* is considered clinically significant if there is pitting edema above the patient's ankle. A thorough evaluation of potential causes is conducted, and the dose of study medication is reduced (if necessary, medication is stopped). As with any serious adverse event, if the patient is experiencing severe symptoms along with clinically significant edema, the SAE should be reported according to study procedures.

- *Severe hypoglycemia* is defined by the need to be treated with glucagon, the need for a third party to resolve a hypoglycemic episode, or loss of consciousness or seizure. Study medications are adjusted downward. If after one month there are no additional hypoglycemic events and if HbA1c values rise > 6%, then the dose of study medications is returned to the previous dose. In case of a second episode of severe hypoglycemia, the dose is decreased until the end of the trial. If more than two severe hypoglycemic events occur in the same patient in the absence of insulin, then the patient is unmasked and study medications are adjusted downward until hypoglycemia is resolved.

  If severe hypoglycemia occurs while on insulin, the insulin dose is reduced by 20%. After one month, if there are not additional hypoglycemic events or if HbA1c values rise to > 6%, then the dose is increased by 10% a month until it returns to the previous level. If a second episode of severe hypoglycemia occurs, insulin dosage adjustment is performed at the discretion of the investigator.

- *Study medication* is temporarily discontinued 24 hours before, during, and for 48 hours after any of the following events: 1) procedure involving the injection of contrast dye; 2)

surgery or other procedure requiring general anesthesia; 3) any illness that could be associated with hypoxia, circulatory failure, or dehydration; 4) hospitalization. Serum creatinine should be rechecked and creatinine clearance calculated as soon as feasible (but no sooner than 48 hours after the conclusion of the event) and study medication can be restarted if the calculated creatinine clearance is ≥ 70 mL/min. If values remain < 70 mL/min, a creatinine clearance is repeated at 2, 4, 8, and 12 weeks. Study medication is restarted when the creatinine clearance is ≥ 70 mL/min. If creatinine clearance remains < 70 mL/min at 12 weeks, then study medication is permanently discontinued.

- Other indications for temporary or permanent discontinuation of study medication include:
  - *Pregnancy:* No study medication should be administered for the duration of pregnancy and insulin should be instituted.
  - *Lactation*: No study medication should be administered to women who are nursing a baby.
  - *Lactic acidosis:* Any study patient who experiences a bout of lactic acidosis has study medication permanently discontinued.
  - *DKA:* Any study patient who experiences an episode of DKA follows the safety procedures for a participant who has experienced metabolic decompensation.
  - *Dermatological problems:* Any study patient who experiences severe dermatological problems, such as urticaria, bullous rashes, exfoliative dermatitis, Stevens-Johnson syndrome, thought to be related to study medication has study medication permanently discontinued.

6.3 Unmasking

Unblinded treatment assignments are held by the coordinating center, the Drug Distribution Center, and, if used, the clinical center central pharmacy.

Each TODAY participant is given a card with clear instructions how to contact a TODAY study representative. The TODAY clinical center nurse coordinator is the primary contact during working hours, the TODAY clinical center on-call physician is the primary contact after clinic hours, and the TODAY Drug Distribution Center is the backup contact. In case of emergency, the patient hands the card to the emergency health care provider who makes the contact as needed. The TODAY clinical center nurse or physician contacts the study chair (or the medical consultant, if the study chair is unavailable) in case of an emergency, illness, or condition that may warrant unmasking. The decision to unmask is made by the study chair (or the medical consultant, in the absence of the study chair).

Every attempt is made to avoid unnecessary unmasking.

6.4 Adverse Event and Serious Adverse Event Reporting

6.4.1 Purpose of Adverse Event Reporting

The reporting of adverse events experienced by study participants meets three important purposes:

1. It identifies the frequency and severity of known and unanticipated side effects of the study interventions (metformin, rosiglitazone, TLP) within each study arm of the trial.

2. It provides the mechanism for reporting the occurrence and severity of adverse events to the study group, the NIH, the FDA, and the pharmaceutical company(s) providing the medications.
3. It fulfills the FDA requirements for reporting adverse reactions to medications.

The timely and complete reporting of adverse events is a critical requirement in the conduct of this trial.

6.4.2 Definitions of Adverse Events

- *Adverse Event (AE):* Any unfavorable and unintended change in the structure, function or chemistry of the body experienced by a study participant during the study regardless of the relationship of this change to administration of study intervention or participation in the study. Adverse events include symptoms and changes in laboratory data that are not specifically part of the primary or secondary outcomes of the trial. AEs are reported only at scheduled study visits unless they meet the criteria for being serious.

- *Serious Adverse Event (SAE):* Events are divided into those that are serious (SAEs) and those that are not serious (AEs). The distinction between an SAE and an AE is a regulatory definition established by the FDA, not a clinical definition. The definition of SAE is not always related to clinical severity of the event. An AE is considered serious (SAE) when it satisfies any one of the following criteria:

  a. The event results in an inpatient hospitalization (any overnight stay associated with an admission).
  b. The event results in the prolongation of a hospital stay.
  c. The event results in permanent or severe disability.
  d. The event results in death.
  e. A pregnancy results in a congenital anomaly.
  f. The event results from an overdose (either accidental or experimental) of the study medication.
  g. The event is life-threatening.
  h. Treatment is required to prevent a serious event.
  i. The patient experiences a bout of lactic acidosis.
  j. An episode of severe hypoglycemia occurs.

6.4.3 Non-serious Adverse Events

It is essential that AEs be ascertained in an unbiased manner using standard questions that are identical and identically administered to patients in all three treatment arms. Therefore, AEs are reported on a standard form that is completed by the study staff at each regular follow-up visit. AEs are ascertained by asking targeted questions relating to specific events of import in diabetic patients on any of the study treatment arms. AEs also include any significantly abnormal physical finding identified on examination and any significantly abnormal laboratory result obtained on the patient between visits or at the time of the visit. Questions answered YES and any new abnormal physical findings are pursued by the study staff in order to determine the seriousness of the event and the need for further evaluation, follow-up, or referral. Adverse events reported or ascertained between clinic visits are captured and reported at the time of the next scheduled visit.

Pre-existing conditions (that is, conditions present prior to randomization) are not considered or recorded as AEs or SAEs unless the condition worsens in intensity or frequency after randomization. Likewise, continuing adverse events are not reported as AEs at subsequent visits unless they increase in severity or frequency between the visits, they result in criteria for an SAE, and/or they resolve between visits.

6.4.4 Serious Adverse Events

Study patients are instructed to contact the clinic with any serious adverse event meeting the above criteria. Each SAE is recorded on the study form and sent to the CoC as soon as possible after they occur and preferably within 24 hours of the notification of the clinic staff. This notification should occur even if data are incomplete. Additional data and follow-up information are sent subsequently as an update to the original report. The CoC immediately forwards SAE reports to the study chair, the NIDDK project office, chair of the Safety and Monitoring Committee, and the DSMB, which convenes expeditiously at the discretion of the chair.

SAEs are also reported to the local IRB and any other institutional monitoring committee, as per local requirements. SAEs are also reported to the FDA if the study is operating under an IND.

6.4.5 Tracking of Adverse Events by the Study Group

- *Serious adverse events:* All SAEs are reported to the CoC within 24 hours. The CoC forwards all SAE reports to the chair of the Safety and Monitoring Committee for consideration. The committee chair assesses each event to determine if immediate action is required by the study group in response to the event. If the chair determines that immediate action should be considered, he/she consults with other members of the committee to recommend a course of action. In addition, any SAE that results in death or permanent or severe disability and any SAE judged by the local PI as PROBABLY or DEFINITELY related to study participation are discussed by the committee as soon as feasible. Any actions recommended are communicated to the study chair for consideration of study wide action. If the SAE is not deemed to warrant immediate study wide action, it is discussed at the next scheduled meeting of the Safety and Monitoring Committee.

- *Non-serious adverse events:* Non-serious adverse events (AEs) are tabulated by the Coordinating Center in the same format as is done for the Data and Safety Monitoring Board (DSMB). Summaries of the AEs, tabulated by clinic, are provided to the Safety and Monitoring Committee and discussed by them before each Steering Committee meeting. The committee reviews this summary during one of its regularly scheduled meetings and makes recommendations for action to the study group at the next Steering Committee meeting.

- *Meetings of the Safety and Monitoring Committee (SMC):* The SMC meets in person at the time of each study group meeting and by conference call at least every 4-6 weeks. During these meetings, the committee discusses all SAEs and, when available, the summary reports of the non-serious AEs. The committee considers whether changes in the protocol (monitoring, consent process, etc.) are indicated based on the occurrence, frequency, or severity of AEs and SAEs. The committee also evaluates whether there is any clustering of AEs by clinic. As deemed necessary, a member of the SMC

communicates with the local center PI to obtain additional information about SAEs and observed local trends in non-serious AEs. The SMC remains blinded to patient treatment group. If an SAE or pattern of AEs warrants unblinding, the issue is referred to the DSMB.

7 Data Processing and Management

The CoC develops and maintains a central database integrating all of the project data.

7.1 Data Management System

Data are entered at the clinical centers into a web data entry application that facilitates direct entry of study data into the study's database. The web application guides the study staff member through the data entry process. If an invalid response is entered, the website signals and provides a message about the error and how to solve it. At any point during entry, the staff member can make an electronic note concerning a particular response. Valid individual responses are saved as soon as they are entered. The system includes programmed skip patterns as required by the case report forms, and also includes quality control checks such as lists of valid values for multiple choice items. The system provides automated consistency checking so the study staff can resolve inconsistencies quickly without a lengthy communication with the coordinating center. The same checking is also run on the central database at the coordinating center to verify that centers are resolving consistency checks.

The CoC provides a number of reports that help the study staff manage the study at their clinic, for example, graphs of recruitment versus goal by race and gender, individual patient schedules and summaries, and inventories of forms completed.

7.2 Data Transfer

Newly entered clinical center data are received by the coordinating center immediately upon data entry. The coordinating center merges newly received data with the accumulated data in a SAS database.

7.3 Central Data Management System

After the first level of editing, data transferred from the clinical centers are imported into the official study SAS database on the Biostatistics Center's IBM 390 enterprise server. The coordinating center performs quality control checks on the data (see below), querying the clinical center regarding data issues and adding corrections to the central database.

7.4 Quality Control

Range checks, inter-item checks, cross-table checks, and double data entry verification are used where appropriate to ensure accurate data entry. Specific quality control procedures are run to check for missing, incorrect, and questionable values immediately after they are entered. Reports with the necessary patient identifying information and the problem values are printed and sent to the clinical centers for correction. When returned, corrected values are entered and checked again for consistency with other items. The goals are to make quality control a continuous process, to make the turnaround time between error detection and correction as short as possible, and to document any changes made to the database.

7.5 Backup, Data Security, and Confidentiality

The Biostatistics Center's data backup and security policies ensure the safety and confidentiality of the data. Backup procedures include: twice-weekly system backup, daily incremental backup, and off-site fire proof storage. Security procedures include: logon and link password protection, remote password logon and dial-back modems, and for internet access, separate Web servers which use SSL and encryption algorithms. Regularly updated virus scanning software is used routinely to check personal computers for computer viruses. University computing facilities provide support in the event of a disaster.

The coordinating center maintains confidentiality of patient data and emerging results per a confidentiality policy, which every staff member is required to sign annually.

7.6 Tracking Study Progress

The purpose of tracking reports is to keep the collaborative group informed of study progress, and to report special problems and resolutions. Reports are produced regularly by the coordinating center, as directed by the Steering Committee. These reports are distributed to the study group through the study website.

Tracking reports include the following types of information:

- screening and enrollment (versus goal), by clinical center, gender, and race/ethnicity
- tables describing adherence to the study protocol (attendance at scheduled study visits, study intervention compliance)
- database inventory
- number of data edit queries generated and outstanding, by clinical center
- characteristics of the patient population, by clinical center
- progress of analysis and manuscripts

7.7 Archival and Study Close-out

At the end of the study, after all data have been received and edited, the database is archived in computer readable format, including: readme documentation files, text files of study documents (forms annotated with variable names, protocols, and manuals of procedures), data files in the form of SAS transport files and input statements, data dictionaries, and program code documenting primary derived variables.

After the results have been published, all data will be available to other investigators. Data will be stored at a readily accessible site.

8 Statistical Considerations

8.1 Sample Size

The primary outcome for the TODAY study is time to treatment failure, defined as persistently elevated HbA1c levels (≥ 8.0% for 6 months) or the inability to be weaned safely from temporary use of insulin therapy. No published data exist that allow estimation of the rate of occurrence. From unpublished data collected in the participating clinical centers, it is estimated that between 10-20% of children will have an HbA1c above the threshold by the end of each 6 month period, beginning at the end of the first year. The following assumptions were used to determine the sample size goal for TODAY:

- Primary outcome is time to failure as defined above.
- Eligible patients are randomized evenly to one of three treatment arms and followed for an additional two years following close of randomization.
- Rate of treatment failure within treatment group is constant over time.
- Treatment failure in the reference group (metformin alone) is at least 10% per six-month period, beginning at the first annual visit.
- There is a reduction of at least 40-50% in the treatment failure rate in one of the more intensively treated groups.
- Ten percent of patients are lost to drop-out during each 6 month period.
- The type I error rate (α) is 0.05 (two-sided) with a Bonferroni adjustment [Miller 1981] for three pair-wise comparisons of the three treatment groups.

Given these assumptions, if 10% of the metformin group has an HbA1c above the threshold, 250 participants per arm provides at least 90% power to detect a 50% reduction in the treatment failure rates in at least one combination therapy group (i.e., 5% with HbA1c above threshold during a six-month period). If 20% of the metformin group has an HbA1c above the threshold, 250 participants per treatment arm provides at least 90% power to detect a 40% reduction in hazard rates in at least one of the combination therapy groups (i.e., 12% failing during a six-month period). Therefore, 250 participants per group are recruited.

8.2 Statistical Analysis

The principal analyses of primary and secondary outcomes employ the "intent-to-treat" approach [Peduzzi et al. 1993]. The intent-to-treat analyses include all randomized patients with all patients included in their randomly assigned treatment group; treatment group assignment is not altered based on the patient's adherence to the assigned treatment regimen. All statistical tests are two-sided with the overall significance level of the primary outcome α □□ =0.05. However, b[illegible]
TODAY, the significance levels used in the interim and final analyses of the primary outcome are adjusted to account for the multiplicity of interim analyses.

- *Baseline characteristics:* Comparison of the baseline characteristics among the three treatment groups uses standard nonparametric statistical techniques, such as Fisher's exact test for categorical data and the Kruskal-Wallis test for ordinal or continuous data.

- *Primary outcome:* The principal analysis of TODAY is an analysis of the time to treatment failure. Turnbull's [1976] algorithm is used to calculate and compare Peto's [1973] nonparametric estimates of the interval-censored survival curves for each treatment group. For the primary outcome analysis, patients are considered "administratively censored" if they complete the full duration of TODAY without having failed. Patients who prematurely discontinue their follow-up visits without having reached study endpoint are "censored" as of their last follow-up visit. Failure is considered to have occurred during the interval between the last HbA1c value < 8% and the first HbA1c value used to classify the participant as a treatment failure.

- *Secondary outcomes:* Secondary time to "event" outcomes (e.g., cardiovascular risk factors, microvascular outcomes) are analyzed using the same life-table methods described above for the primary outcome. A proportional-hazards regression model is used to evaluate potential covariates that may modify the primary and secondary time to

event outcomes (e.g., risk population defined by race/ethnicity, age, clinical site). Graphical procedures are used to assess the proportionality assumption. If the proportionality assumption is found to be unreasonable, then other models such as the accelerated failure time model [Wei 1992] or the proportional odds model [McCullagh 1980] are used to evaluate the covariates.

Life-table analysis assesses the risk of the first event in time. Some processes, however, involve recurrent events such as the back-and-forth between elevated and normal blood pressure. For these recurrent events, the family of statistical models based on the theory of counting processes are applied [Fleming and Harrington 1991].

Longitudinal data analysis techniques are used to analyze repeated measures data (e.g., glycemia, fasting lipids, blood pressure, physical activity, quality of life). These include: (1) analyses of the point prevalence of a discrete characteristic (e.g., hypertension) at successive repeated visits over time [Lachin and Wei 1988]; (2) multivariate rank analyses of quantitative (e.g., 2 hour OGTT) or ordinal (e.g., the Child Health Questionnaire subscales) measures over successive visits [Wei and Lachin 1984]; (3) the parametric linear random effects model of Laird and Ware [1982] to compare participant slopes over time (e.g., rate of change in fasting glucose) under linearity and normality assumptions; and (4) techniques developed by Liang and Zeger [1986] to compare participant slopes under a generalized linear models framework.

- *Interim analysis:* The Lan-DeMets [1983] spending function approach is used to adjust the probability of a type I error for testing the primary outcome when interim 'looks' of the data are taken by the Data Safety Monitoring Board. The spending function corresponding to an O'Brien and Fleming [1979] boundary are used. The Lan-DeMets procedure is flexible, in that the number of looks does not have to be specified in advance and the time interval between looks does not have to be the same throughout TODAY. The rate at which the type I error is spent is a function of the fraction of total information available at the time of the interim analysis (i.e., information time). For an interim analysis using the logrank test (i.e., time to confirmed treatment failure), the information time is the fraction of the total number of confirmed treatment failures to be accrued in the entire TODAY. Since the total number of failures to be accrued is unknown, an estimate of the information time is based on the fraction of total patient exposure [Lan and Lachin 1990].

9 Study Administration

9.1 Organization

The major organizational components and their responsibilities are described:

- The *STOPP-T2D Steering Committee*, composed of the principal investigators of the 3 Prevention field centers and the 12 Treatment clinical centers, the coordinating center, the NIDDK project office, the study chair and designated expert investigators, is the primary decision making body for the study with overall responsibility for the design and conduct of study protocols.
- The *Treatment Protocol Committee* is a subgroup of the Steering Committee, and is composed of investigators from the twelve treatment clinical centers, the coordinating center, the NIDDK project office, the study chair and designated experts. The committee is responsible for the design and conduct of the treatment clinical trial.
- The *NIDDK project office* participates in all decision-making activities and selects and oversees the activities of the Data Safety Monitoring Board.

- The *clinical centers* are located at Baylor College of Medicine, Case Western Reserve University, Children's Hospital of Los Angeles, Children's Hospital of Philadelphia, Children's Hospital of Pittsburgh, Massachusetts General Hospital, State University of New York Upstate Medical University, University of Colorado Health Sciences Center, University of Oklahoma Health Sciences Center, University of Texas Health Sciences Center at San Antonio, Washington University at St Louis, and Yale University. They are responsible for recruiting patients and implementing the protocol.
- The *coordinating center* is located at the George Washington University Biostatistics Center with responsibility for coordinating all aspects of the study, including production and distribution of materials and documents, set-up and administration of the data management system, maintenance of the central database, analysis of results, and report of results in collaboration with the other investigators.
- The *Central Blood Laboratory (CBL)* operates under subcontract to the coordinating center. The CBL is responsible for providing procedures for the handling, storage, and shipment of blood specimens, for performing the tests and assays, for performing quality control, and for transferring results to the coordinating center.
- The *Drug Distribution Center (DDC)* operates under subcontract to the coordinating center and is responsible for packaging and distributing study drug, designing masked labels, providing procedures and training for ordering, handling, shipping, and storage, and working with the coordinating center to devise and implement a drug and dosage administration scheme.
- The *Diet Assessment Center (DAC)* operates under subcontract to the coordinating center and is responsible for designing, implementing, and analyzing the diet data collected.
- The *DXA Central Reading Center (DCRC)* operates under subcontract to the coordinating center and is responsible for interpreting and analyzing the DXA scan data.
- The *Data Safety Monitoring Board (DSMB)* is composed of outside experts in the design and conduct of clinical trials, in pediatrics, and in T2DM. The board is responsible for reviewing the study documents, monitoring study progress, and monitoring patient safety.
- The *Lifestyle Materials Core (LMC)* is responsible for providing culturally competent, developmentally appropriate materials to be used in the intensive lifestyle intervention and for providing training and oversight of TLP staff.
- *Working committees* include Objectives and Outcomes, Recruitment and Retention, Safety and Monitoring, Adherence, Standard Education Intervention, Intensive Lifestyle Intervention, Pharmacology, and Protocol Review.

9.2 Central Laboratories and Reading Centers

In collaboration with the coordinating center and study investigators, central laboratories and reading centers perform the following tasks:

1. Establish procedures and standards for training staff involved in the measurement, collection, preparation, handling, transfer, and all other procedures and processes.
2. Conduct training sessions and contribute training materials to the study manuals of procedures.
3. Provide or facilitate the acquisition of equipment and materials, including specifying brands, sizes, and suppliers as applicable.
4. Establish procedures for data entry and transfer of data to CoC.

5. Develop procedures for the internal as well as external quality control, and provide periodic reports on the quality control surveillance.
6. Provide long-term storage of reserve specimens or materials as directed by the Steering Committee for use in ancillary or future studies.

Each director represents the laboratory or center at STOPP-T2D Steering Committee meetings, on Steering Committee conference calls, and on other conference calls where the director's participation is deemed necessary.

9.3 Training and Certification

During the start-up period, the CoC holds a training workshop for study staff. Investigators may provide instruction in various aspects of the study. The purpose of the training workshop is to provide training for study staff in order to assure that the study is conducted in a standardized manner across all participating centers. The training, based on the study manual of procedures, includes the study design, eligibility criteria, conducting patient assessments, patient follow-up schedule, use of the distributed data entry software and electronic forms, transferring data to the CoC, maintaining patient and data confidentiality, and patient treatment guidelines. Throughout the study, new staff are trained by the clinical center principal investigator, study staff and by the CoC. If a new PAL joins the study staff after the trial has begun, two non-study patients will be recruited for PAL training purposes.

Prior to being allowed to recruit patients, each clinical center must pass certification criteria, including supplying the coordinating center with the IRB approval letter and stamped informed consent forms, completion of conflict of interest policy by all investigators, completion of the outcomes assessment pilot, and commencement of the TLP pilot with two non-study patients.

9.4 Site Visits

The two types of site visits are (1) scheduled monitoring and (2) as needed to address specific problems.

The CoC organizes site visits necessary to monitor study procedures and records. The site visit team includes representatives from the CoC, investigator(s) or coordinator(s) from other clinical centers, and if possible, a representative of the NIDDK program office. Each visit follows a predetermined format and site visitors complete a checklist to record findings. The site visit team reviews study procedures and compares data collection records to listings from the central database.

Site visits conducted to address specific problems at the clinical center are attended by the study chair, the NIDDK project office, the CoC, and others as needed.

9.5 Study Website

The CoC maintains the study website, which is a secure site requiring a user ID and password combination for access. The web server utilizes the Secure Socket Layer (SSL) protocol that encrypts all traffic to and from the server. Investigators, coordinators, consultants, and other study staff who would benefit from access to the information on the website are each given a unique user ID and password, which identifies the user to the web server and can be used to restrict access to particular web pages if desired.

The website contains study documents such as the protocol, manual of procedures, and forms, study calendar, directory, meeting and conference call information, links to other sites, tracking reports, minutes, and agendas.

9.6 Conflict of Interest Policy

The STOPP-T2D investigators have adopted a conflict of interest policy similar to that used by other NIDDK collaborative groups. On an annual basis or whenever there is a significant change in status, STOPP-T2D collaborators are required to disclose any financial or related interest that could present an actual conflict of interest or be perceived to present a conflict of interest. Disclosure is required to protect each individual's reputation and career from potentially embarrassing or harmful allegations of inappropriate behavior, and to protect the integrity of STOPP-T2D study research. Forms are kept on file at the CoC.

The STOPP-T2D Ethics Committee determines (1) if the disclosed interests could directly and significantly affect the performance of study responsibilities and (2) the management, reduction, or elimination of the conflict. In addition to complying with the STOPP-T2D conflict of interest policies, collaborators must certify to the Ethics Committee that they have complied with all of their local and institutional requirements regarding conflict of interest and disclosure. This is accomplished by supplying the CoC with copies of the local IRB letter of approval and stamped informed consent form(s).

9.7 Publications and Presentations Policy

The STOPP-T2D investigators have adopted a policy similar to those used by other NIDDK collaborative groups. The policy is administered by the STOPP-T2D Publications Committee with approval from the STOPP-T2D Steering Committee. The policy includes guidelines for authorship, submission and review of proposed publications and presentations, ownership of the data, and setting priorities for CoC statisticians.

9.8 Protocol Amendments

Adoption of protocol amendments requires two-thirds majority approval by voting members of the TODAY Treatment Protocol Committee. The amended protocol is resubmitted to the IRB.

10 Literature Cited

Adams M, Montague CT, Prins JB, Holder JC, Smith SA, Sanders L, Digby JE, Sewer CP, Lazar MA, Chatterjee KK, O'Rahilly S. Activators of peroxisomal proliferator activated receptor gamma have depot-specific effects on human pre-adipocyte differentiation. *J Clin Invest* 1997; 100: 3149-3153.

American Diabetes Association. Type 2 diabetes in children and adolescents consensus statement. *Diabetes Care* 2000; 23:381-389.

American Diabetes Association and National Institute of Diabetes, Digestive and Kidney Disease. The prevention or delay of type 2 diabetes. *Diabetes Care* 2002; 25:742-749.

American Diabetes Association. Evidence-based nutrition principles and recommendations for the treatment and prevention of diabetes and related complications. *Diabetes Care* 2002; 25(Suppl 1):S50-S60.

American Diabetes Association. Economic costs of diabetes in the US in 2002. *Diabetes Care* 2003; 26:917-932.

Aronoff S, Rosenblatt S, Braithwaite, et al. Pioglitazone hydrochloride monotherapy improves glycemic control in their treatment of patients with type 2 diabetes. *Diabetes Care* 2000; 23:1605-1611.

Arslanian SA, Kalhan SC. Correlations between fatty acid and glucose metabolism: potential explanation of insulin resistance during puberty. *Diabetes* 1994; 43:908-914.

Arslanian S. Type 2 diabetes in children: pathophysiology and risk factors. *J Ped Endocrinol Metab* 2000; 13(6):1385-1394.

Bailey CJ, Turner RC. Metformin. *N Engl J Med* 1996; 334(9):574-579.

Beck AT, Ward CH, Mendelson M, Mock J, Erbaugh J. An inventory for measuring depression. *Arch General Psychiatry* 1961; 4:53-63.

Beck AT, Steer RA, Garbin MG. Psychometric properties of the Beck Depression Inventory: twenty-five years of evaluation. *Clin Psychol Rev* 1988; 8:77-100.

Beck AT, Steer RA. *Beck Depression Inventory Manual*. San Antonio, Harcourt Brace & Company, 1993.

Beck AT, Steer RA. *Beck Depression Inventory II*. San Antonio, Psychological Corp, 1996.

Blackburn GL. Effect of degree of weight loss on health benefits. *Obes Res* 1995; 3:211s-216s.

Bonora E, Targher G, Alberiche M, Bonadonna RC, Saggiani F, Zenere MB, Monauni T, Muggeo M. Homeostasis model assessment closely mirrors the glucose clamp technique in the assessment of insulin sensitivity: studies in subjects with various degrees of glucose tolerance and insulin sensitivity. *Diabetes Care* 2000; 23(1):57-63.

Boreham CA, Paliczka VJ, Nichols AK. A comparison of the PWC170 and 20-MST tests of aerobic fitness in adolescent schoolchildren. *J Sports Med Phys Fitness* 1990; 30(1):19-23.

Breda E, Cavaghan MK, Toffolo G, Polonsky KS, Cobelli C. Oral glucose tolerance test minimal model indexes of beta-cell function and insulin sensitivity. *Diabetes* 2001; 50(1):150-158.

Brown SA. Studies of educational interventions and outcomes in diabetic adults: a meta-analysis revisited. *Patient Education and Counseling*, 1990; 16:189-215.

Brown SA. Meta-analysis of diabetes patient education research: variations in intervention effects across studies. *Research in Nursing and Health* 1992; 15(6):409-419.

Brownell KD, Kelman JH, Stunkard AJ. Treatment of obese children with and without their mothers: changes in weight and blood pressure. *Pediatrics* 1983; 71(4):515-523.

Campbell IW, Howlett HC. Worldwide experience of metformin as an effective glucose-lowering agent, a meta-analysis. *Diabetes Metab Rev* 1995; 11:S57-S62.

Chaturvedi N, Fuller JH. Mortality risk by body weight and weight change in people with NIDDM: The WHO Multinational Study of Vascular Disease in Diabetes. *Diabetes Care* 1995; 18:766-774.

Chinali M, de Simone G, Roman MJ, Best LG, Lee ET, Russell M, Howard BV, Devereux RB. Cardiac markers of pre-clinical disease in adolescents with metabolic syndrome. *Journal of the American College of Cardiology* 52: 932-938.

Coditz GA, Willett WC, Stampfer MJ, Manson JE, Hennedkens CH, Arky RA, Speizer FE. Weight as a risk factor for clinical diabetes in women. *Amer J Epidemiol* 1990; 132: 501-513.

Cote KD, Adams WC. Effect of bone density on body composition estimates in young adult black and white women. *Med Sci Sports Exerc* 1993; 25(2):290-296.

Cusi K, Consoli A, DeFronzo RA. Metabolic effects of metformin on glucose and lactate metabolism in non-insulin-dependent diabetes mellitus. *J Clin Endocrinol Metab* 1996; 81:4059-4067.

Dabelea D, Petit DJ, Jones KL, Arslanian SA. Type 2 diabetes mellitus in minority children and adolescents: an emerging problem. *Endocrinol Metab Clin North Am* 1999; 28(4): 709-729.

DCCT Research Group. Lifetime benefits and costs of intensive therapy as practiced in the diabetes control and complications trial. *JAMA* 1996; 276:1409-1415.

Dean HJ, Young TK, Flett B, Wood-Steinmen P. Screening for type 2 diabetes in northern Canada. *Lancet* 1998; 352:1523-1524.

Dean H, Flett B. Natural history of type 2 diabetes diagnosed in childhood: long term follow-up in young adult years. *Diabetes* 2002; 22(Suppl 1):S5-S19.

DeFronzo RA. Pharmacologic therapy in type 2 diabetes mellitus. *Ann Intern Med* 1999; 131:281-303.

DeFronzo RA, Goodman AM. Efficacy of metformin in patients with non-insulin-dependent diabetes mellitus. *N Engl J Med* 1995; 333:541-549.

DeFronzo RA, Barzilai N, Simonson DC. Mechanism of metformin action in obese and lean non-insulin-dependent diabetes mellitus. *J Clin Endocrinol Metab* 1991; 73:1294-1301.

de Maat MP, Pietersma A, Kofflard M, Sluiter W, Kluft C. Association of plasma fibrinogen levels with coronary artery disease, smoking and inflammatory markers. *Atherosclerosis.* 1996; 121(2):185-191.

Diabetes Prevention Program Research Group. Reduction in the incidence of type 2 diabetes with lifestyle intervention or metformin. *NEJM* 2002; 346:393-403.

Dietz WH. Health consequences of obesity in youth: childhood predictors of adult disease. *Pediatrics* 1998; 101(3):518-524.

Dounchis JZ, Hayden HA, Wilfley, DE. Obesity, body image, and eating disorders in ethnically diverse children and adolescents. In Thompson JK, Smolak L (eds), *Body Image, Eating Disorders, and Obesity in Youth: Assessment, Prevention, and Treatment*, Washington DC, American Psychological Association, 2001, 76-98.

Drummond MF, O'Brien B, Stoddart GL, Torrance GW. *Methods for the Economic Evaluation of Health Care Programmes*, 2nd edition, Oxford University Press, New York, 1999.

Ellis KJ, Shypailo RJ. Bone mineral and body composition measurements: cross-calibration of pencil-beam and fan-beam dual-energy X-ray absorptiometers. *J Bone Miner Res* 1998; 13:1613-1618.

Epstein LH, Klein KR, Wisniewski L. Child and parent factors that influence psychological problems in obese children. *Int J Eat Disord* 1994; 15:151-158.

Epstein LH, Myers MD, Raynor HA, Saelens BE. Treatment of pediatric obesity. *Pediatrics* 1998; 101(3):554-570.

Epstein LH, Squires S. *The Stoplight Diet for Children: An Eight-week Program for Parents and Children*, Boston, MA, Little, Brown and Company, 1998.

Estelles A, Dalmau J, Falco C, Berbel O, Castello R, Espana F, Aznar J. Plasma PAI-1 levels in obese children--effect of weight loss and influence of PAI-1 promoter 4G/5G genotype. *Thromb Haemost* 2001; 86(2):647-652.

Fagot-Campagna A, Petit DJ, Engelgau M. Type 2 diabetes among North American children and adolescents: an epidemiologic review and a public health perspective. *J Pediatr* 2000; 136:664-672.

Faith MS, Saelens BE, Wilfley DE, Allison DB. Behavioral treatment of childhood and adolescent obesity: current status, challenges, and future directions. In Thompson JK, Smolak L (eds), *Body Image, Eating Disorders, and Obesity in Youth: Assessment, Prevention, and Treatment,* Washington DC, American Psychological Association, 2001, 313-340.

Feldman EL, Stevens MJ, Thomas PK, Brown MB, Canal N, Greene DA. A practical two-step quantitative clinical and electrophysiological assessment for the diagnosis and staging of diabetic neuropathy. *Diabetes Care* 1994; 17(11):1281-1289.

Ferguson MA, Gutin B, Owens S, Litaker M, Tracy RP, Allison J. Fat distribution and hemostatic measures in obese children. *Am J Clin Nutr* 1998; 67(6):1136-1140.

Fleming TR, Harrington DP. *Counting process and survival analysis*. New York: Wiley, 1991.

Fonseca V, Rosenstock J, Patwardhan R, Salzman A. Effect of metformin and rosiglitazone combination therapy in patients with type 2 diabetes mellitus: a randomized controlled trial. *JAMA* 2000; 283:1695-1702.

Fontbonne A, Charles MA, Juhan-Vague I, Bard JM, Andre P, Isnard F, Cohen JM, Grandmottet P, Vague P, Safar ME, Eschwege E for the BIGPRO Study Group. The effect of metformin on the metabolic abnormalities associated with upper-body fat distribution. *Diabetes Care* 1996; 19(9):920-6.

Furlong WJ, Feeny DH, Torrance GW, Barr RD. The Health Utilities Index (HUI) system for assessing health-related quality of life in clinical studies. *Annals of Medicine* 2001;33:375-384.

Gallistl S, Sudi KM, Aigner R, Borkenstein M. Changes in serum interleukin-6 concentrations in obese children and adolescents during a weight reduction program. *Int J Obes Relat Metab Disord* 2001; 25(11):1640-3

Garber AJ, Duncan TG, Goodman AM, Mills DJ, Rohlf JL. Efficacy of metformin in type II diabetes: results of a double-blind, placebo-controlled, dose-response trial. *Am J Med* 1997; 103:491-497.

Girard J. Fatty acid and beta cells. *Diabetes Metab* 2000; 26(3):6-9.

Golan M, Weizman A, Apter A, Fainaru M. Parents as the exclusive agents of change in the treatment of childhood obesity. *Am J Clin Nut* 1998; 67:1130-1135.

Gold MR, Siegel JE, Russell LB, Weinstein MC. *Cost-effectiveness in Health and Medicine*, Oxford University Press, New York, 1996.

Goldfield GS, Epstein LH. Management of obesity in children. In Fairburn CG, Brownell KD (eds), *Eating Disorders and Obesity*, 2nd edition, New York, NY, Guilford Press, 2002, 573-577.

Goldstein DJ. Beneficial health effects of modest weight loss. *Int J Obes* 1992; 16:397-415.

Goran MI, Driscoll P, Johnson R, Nagy TR, Hunter G. Cross-calibration of body-composition techniques against dual-energy X-ray absorptiometry in young children. *Am J Clin Nutr* 1996; 63(3):299-305.

Goran MI, Figueroa R, McGloin A, Nguyen V, Treuth MS, Nagy TR. Obesity in children: recent advances in energy metabolism and body composition. *Obesity Res* 1995; 3:277-289.

Gray A, Raikou M, McGuire A, Fenn P, Stevens R, Cull C, Stratton I, Adler A, Holman R, Turner R. Cost effectiveness of an intensive blood glucose control policy in patients with type 2 diabetes: economic analysis alongside randomised controlled trial (UKPDS 41). *BMJ* 2000; 320:1373-1378.

Greene DA. Rosiglitazone: a new therapy for type 2 diabetes. *Expert Opin Investig Drugs* 1999; 8:1709-1719.

Guzzaloni G, Grugni G, Mazzilli G, Moro D, Morabito F. Comparison between beta-cell function and insulin resistance indexes in prepubertal and pubertal obese children. *Metabolism* 2002; 51(8):1011-6.

Henry RR, Wallace P, Olefsky JM. Effects of weight loss on mechanisms of hyperglycemia in obese non-insulin-dependent diabetes mellitus. *Diabetes* 1986; 35(9):990-8

Hermans MP, Levy JC, Morris RJ, Turner RC. Comparison of tests of beta-cell function across a range of glucose tolerance from normal to diabetes. *Diabetes* 1999; 48(9):1779-86.

Howley ET, Bassett DR Jr, Welch HG. Criteria for maximal oxygen uptake: review and commentary. *Med Sci Sports Exerc* 1995; 27(9):1292-301.

Jacobs DR Jr, Ainsworth BE, Hartman TJ, Leon AS. A simultaneous evaluation of 10 commonly used physical activity questionnaires. *Med Sci Sports Exerc* 1993; 25(1):81-91.

Janz KF. Validation of the CSA accelerometer for assessing children's physical activity. *Med Sci Sports Exerc* 1994*;* 26(3):369-75.

Jeppesen J, Zhou MY, Chen YD, Reaven GM. Effect of metformin on postprandial lipemia in patients with fairly or poorly controlled NIDDM. *Diabetes Care* 1994; 17:1099.

Johnson AB, Webster JM, Sum CF, Hesltine L, Argyraki M, Cooper BG. The impact of metformin therapy on hepatic glucose production and skeletal muscle glycogen synthase activity in overweight type 2 diabetic patients. *Metabolism* 1993; 42:1217-1222.

Jones KL, Arslanian S, Peterokova VA, Park JS, Tomlinson MJ. Effect of metformin in pediatric patients with type 2 diabetes: a randomized controlled trial. *Diabetes Care* 2002; 25:89-94.

Jones KL. Non-insulin dependent diabetes in children and adolescents: the therapeutic challenge. *Clin Pediatr* 1998; 37:103-110.

Juhan-Vague I, Morange P, Christine Alessi M. Fibrinolytic function and coronary risk. *Curr Cardiol Rep* 1999 Jul; 1(2):119-24.

Kalbfleisch JD, Prentice RL. *The Statistical Analysis of Failure Time Data*, New York, John Wiley, 1980.

Katz A, Nambi SS, Mather K, Baron AD, Follmann DA, Sullivan G, Quon MJ. Quantitative insulin sensitivity check index: a simple, accurate method for assessing insulin sensitivity in humans. *J Clin Endocrinol Metab* 2000 Jul;85(7):2402-10.

Kaufman FR. Type 2 diabetes in children and youth. *Review Endo Metab Disor* 2003; 4:33-42.

Kay JP, Alemzadeh R, Langley G, D'Angelo L, Smith P, Holshouser S. *Metab* 2001; 50: 1457-1461.

Kelly DE, Killian D. Troglitazone. *Curr Opin Endocrinol Diabetes* 1998; 5:90-96.

Kemnitz JW, Elson DF, Roecker EB, Baum ST, Bergman RN, Meglasson MD. Pioglitazone increases insulin sensitivity, reduces blood glucose, insulin and lipid levels, and lowers blood pressure in obese, insulin-resistant rhesus monkeys. *Diabetes* 1994; 43:204-211.

Kendall PC, Hollon SD, Beck AT, Hammern CH, Ingram RE. Issues and recommendations regarding the use of the Beck Depression Inventory. *Cog Ther Res* 1987; 11:289-299.

Kim C, McHugh C, Kwok Y, Smith A. Type 2 diabetes mellitus in Navajo adolescents. *West J Med* 1999; 170:210-213.

Kimm SYS, Glynn NW, Kriska AM, Fitzgerald SL, Aaron DJ, Similo SL, McMahon RP, Barton BA. Longitudinal changes in physical activity in a biracial cohort during adolescence. *Med Sci Sports Exer* 2000; 32;1445-1454.

Kovacs M, Beck AT. An empirical-clinical approach toward a definition of childhood depression. In Schulterbrandt JG, Raskin A (eds), *Depression in Childhood: Diagnosis, Treatment, and Conceptual Models*, Raven Press, 1997, 1-25.

Kovacs M. Rating scales to assess depression in school-age children. *Acta Paedopsychiatr* 1981; 46:305-310.

Kovacs M. The Children's Depression Inventory. *Psychopharm Bull* 1985; 21(4); 995-998.

Kovacs M. *Children's Depression Inventory Manual*, North Tonawanda, NY, Multi-Health Systems Inc, 1992.

Kriska AM, WC Knowler, RE LaPorte, AL Drash, RR Wing, SN Blair, PH Bennett, and LH Kuller. Development of questionnaire to examine relationship of physical activity and diabetes in Pima Indians. *Diabetes Care* 1990; 13: 401-411.

Fitzgerald SJ, Kriska AM, Pereira MA, de Courten MP. Associations among physical activity, television watching, and obesity in adult Pima Indians. *Med Sci Sports Exerc* 1997 Jul; 29(7):910-5

Kullo IJ, Gau GT, Tajik AJ. Novel risk factors for atherosclerosis. *Mayo Clin Proc* 2000 Apr; 75(4):369-80.

Lachin JM, Wei LJ. Estimators and tests in the analysis of multiple nonindependent 2 x 2 tables with partially missing observations. *Biometrics* 1988; 44:513-528.
Lan KK, DeMets DL. Discrete sequential boundaries for clinical trials. *Biometrika* 1983; 64:191-199.
Lan KK, Lachin JM. Implementation of group sequential logrank tests in a maximum duration trial. *Biometrics* 1990; 46:759-770.
Laird NM, Ware JH. Random-effects models for longitudinal data. *Biometrics* 1982; 38:963-974.
Landgraf JM, Abetz L, Ware JE. *The CHQ User's Manual*, 1st edition, Boston, MA, The Health Institute New England Medical Center, 1996.
Lean MEJ, Powrie JK, Anderson AS, Garthwaite PH. Obesity, weight loss and prognosis in type 2 diabetes. *Diabet Med* 1990; 7:228-233.
Lebovitz HE. Insulin secretogogues: old and new. *Diabetes Rev* 1999; 19:785-830.
Liang K, Zeger SL. Longitudinal data analysis using generalized linear models. *Biometrika* 1986; 73:13-22.
Libman IM, LaPorte RE, Becker D, Dorman JS, Drash AL, Kuller L. Was there an epidemic of diabetes in nonwhite adolescents in Allegheny County, Pennsylvania? *Diabetes Care* 1998; 21(8):1278-1281.
Maggio C, Pi-Sunyer FX. The prevention and treatment of obesity: application to type 2 diabetes. *Diabetes Care* 1997; 20:1744-1766.
Manson JE, Rimm EB, Stampfer MJ, Colditz GA, Willett WC, Krolewski AS, Rosner B, Hennekens CH., Speizer FE. Physical activity and incidence of non-insulin-dependent mellitus in women. *Lancet* 1991; 338:774-778.
Masse LC, Fulton JE, Watson KL, Heesch KC, Kohl HW 3rd, Blair SN, Tortolero SR. Detecting bouts of physical activity in a field setting. *Res Q Exerc Sport* 1999; 70(3):212-9.
Mayer-Davis EJ, Vitolins MZ, Carmichael SL, Hemphill S, Tsaroucha G, Rushing J, Levin S. Validity and reproducibility of a food frequency interview in a multi-cultural epidemiology study. *Ann Epidemiol* 1999; 9(5):314-24.
McCullagh P. Regression models for ordinal data. *J Royal Stat Soc* 1980; 42:109-142.
McGarry JD, Dobbins RL. Fatty acids, lipotoxicity and insulin secretion. *Diabetologia* 1999; 42:128-138.
Meigs JB, Mittleman MA, Nathan DM, Tofler GH, Singer DE, Murphy-Sheehy PM, Lipinska I, D'Agostino RB, Wilson PW. Hyperinsulinemia, hyperglycemia, and impaired hemostasis: the Framingham Offspring Study. *JAMA* 2000; 283(2):221-8.
Melanson EL Jr, Freedson PS. Validity of the Computer Science and Applications, Inc. (CSA) activity monitor. *Med Sci Sports Exerc* 1995; 27(6):934-40.
Miller RG. *Simultaneous Statistical Inference*, New York, Springer-Verlag, 1981.
Miyazaki Y, Mahankali A, Matsuda M, Glass L, Mamankali S, Ferraninie E, Cassi K, Mandarino L, DeFronzo RA. Improved glycemic control and enhanced insulin sensitivity in type 2 diabetic subjects treated with pioglitazone. *Diabetes Care* 2001; 24:710-719.
Miyazaki Y, Mahankali A, Matsuda M, Mahankali S, Hardies J, Cusi K, Mandarino L, DeFronzo RA. Effect of pioglitazone on abdominal fat distribution and insulin sensitivity in type 2 diabetic patients. *J Clin Endocrinol Metab* 2002; 87: 2784-2791.
Nadeau KJ, Zeitler PS, Bauer TA, Brown MS, Dorosz JL, Draznin B, Reusch JE, Regensteiner JG. Insulin resistance in adolescents with type 2 diabetes is associated with impaired exercise capacity. *J Clin Endocrinol Metab* 2009 ;94(10):3687-95.
National Center for Health Statistics. *Plan and Operation of the Third National Health and Nutrition Examination Survey, 1988-1994*, Hyattsville, MD, National Center for Health Statistics, 1994 (Vital and Health Statistics Ser. 1, No.32).

National Heart, Lung, and Blood Institute. Update on the task force report (1987) on high blood pressure in children and adolescents: a working group report from the national high blood pressure education program. National Institutes of Health Publication No. 96-3790, 1996.

Natow AB, Heslin J. *The Fat Counter*, 4th edition, Pocket Books, 1998.

Neufield ND, Raffel LJ, Landon C, Chen YD, Vadheim CM. Early presentation of type 2 diabetes in Mexican-American youth. *Diabetes Care* 1998; 21:80-86.

Nichols JF, Morgan CG, Chabot LE, Sallis JF, Calfas KJ. Assessment of physical activity with the Computer Science and Applications, Inc., accelerometer: laboratory versus field validation. *Res Q Exerc Sport* 2000; 71(1):36-43.

O'Brien PC, Fleming TR. A multiple testing procedure for clinical trials. *Biometrics* 1979; 35:549-556.

Olefsky JM, Saltiel AR. PPARγ and the treatment of insulin resistance. *Trends Endocrinol Metab* 2000; 11:362-368.

Padgett D, Mumford E, Hynes M, Carter R. Meta-analysis of the effects of educational and psychosocial interventions on management of diabetes mellitus. *J Clinical Epid* 1988; 41(10):1007-1030.

Peduzzi P, Wittes J, Detre K, Holford T. Analysis as-randomized and the problem of non-adherence: an example from the Veterans Affairs Randomized Trial of Coronary Artery Bypass Surgery. *Statistics in Medicine* 1993; 12:1185-1195.

Perriello G, Misericordia P, Volpi E, Santucci C, Ferrannini E. Acute antihyperglycemic mechanisms of metformin in NIDDM: evidence for suppression of lipid oxidation and hepatic glucose production. *Diabetes* 1994; 43:920-928.

Peto R. Experimental survival curves for interval-censored data. *Applied Statistics* 1973; 22:86-93.

Phillips LS, Grunberger G, Miller E, et al. Once- and twice-daily dosing with rosiglitazone improves glycemic control in patients with type 2 diabetes. *Diabetes Care* 2001; 24:308-315

Pinhas-Hamiel O, Dolan LM, Daniels SR, Staniford D, Khoury P, Zeitler P. Increased incidence of non-inuslin-dependent diabetes mellitus among adolescents. *J Pediatr* 1996; 128:608-615.

Pouliot MC, Despres JP, Lemieux S, Moorjani S, Bouchard C, Tremblay A, Nadeau A, Lupien PJ. Waist circumference and abdominal sagittal diameter: best simple anthropometric indexes of abdominal visceral adipose tissue accumulation and related cardiovascular risk in men and women. *Am J Cardiol* 1994; 73(7):460-8.

Reaven GM. Banting Lecture 1988: role of insulin resistance in human disease. *Diabetes*. 1988; 37(12):1595-607.

Reynolds WM. Depression in children and adolescents. In Reynolds WM (ed), *Internalizing Disorders in Children and Adolescents*, New York, John Wiley & Sons Inc, 1992.

Rios Burrows N, Acton K, Geiss L, Engelgau M. Trends in diabetes prevalence among American Indian and Alaska Native children, adolescents and young adults, 1991-1997. 11th Annual Indian Health Service Research Conference, Albuquerque, NM, April 1999.

Rockett HR, Colditz GA. *Am J Clin Nutr*. 1997 Apr;65(4 Suppl):1116S-1122S. Review.

Roemmich, J, Clark, P, Lusk, M, Friel, A, Weltman, A, Epstein, L, Rogol A. *Int J Obes Relat Metab Disord* 2002; 26:701-709.

Rosenbloom AL, Je JR, Young RS, Winter WE. Emerging epidemic of type 2 diabetes in youth. *Diabetes Care* 1999; 22:345-354.

Rowland TW, Cunningham LN. Oxygen uptake plateau during maximal treadmill exercise in children. *Chest* 1992; 101(2):485-489

Saelens BE, Sallis JF, Wilfley DE, Patrick K, Cella JA, Buchta R. Behavioral weight control for overweight adolescents initiated in primary care. *Obes Res* 2002; 10:22-32.

Saltiel AL, Olefsky JM. Thiazolidinediones in the treatment of insulin resistance and type 2 diabetes. *Diabetes* 1996; 45:661-669.

Savage PJ, Bennett PH, Senter G, Miller M. High prevalence of diabetes in young Pima Indians: evidence of phenotypic variation and in a genetically isolated population. *Diabetes* 1979; 28:837-842.

Silverstein JH, Rosenbloom AL. Type 2 diabetes in children. *Current Diabetes Reports* 2001; 1:19-27.

Silverstein JH, Rosenbloom AL. Treatment of type 2 diabetes mellitus in children and adolescents. *J Pediatr Endocrinol Metab* 2000; 13:1403-1409.

Sinha R, Dufour S, Petersen KF, LeBon V, Enoksson S, Ma YZ, Savoye M, Rothman DL, Shulman GI, Caprio S. Assessment of skeletal muscle triglyceride content by (1)H nuclear magnetic resonance spectroscopy in lean and obese adolescents: relationships to insulin sensitivity, total body fat, and central adiposity. *Diabetes* 2002; 51(4):1022-1027.

Srinivasan SR, Myers L, Berenson GS. Predictability of childhood adiposity and insulin for developing insulin resistance syndrome (Syndrome X) in young adulthood: the Bogalusa Heart Study. *Diabetes* 2002; 51(1):204-209.

Stumvoll M, Mitrakou A, Pimenta W, Jenssen T, Yki-Jarvinen H, Van Haeften T, Renn W, Gerich J. Use of the oral glucose tolerance test to assess insulin release and insulin sensitivity. *Diabetes Care* 2000; 23(3):295-301.

Stumvoll N, Nurjhan N, Perrieloo G, Dailey G, Gerich JE. Metabolic effects of metformin in non-insulin-dependent diabetes mellitus. *New Engl J Med* 1995; 333:550-554.

Sudi K, Gallistl S, Payerl D, Aigner R, Moller R, Tafeit E, Borkenstein MH. Interrelationship between estimates of adiposity and body fat distribution with metabolic and hemostatic parameters in obese children. *Metabolism* 2001; 50(6):681-7.

Takahashi C, Nagai N, Ujihara N, Babazono T, Nakanishi K, Yokoyama H, Sanaka T, Hirata Y. Clinical profile of Japanese dialysis patients with diabetic nephropathy, diagnosed as having diabetes before the age of thirty. *Diabetes Res Clin Pract* 1990; 10(2):127-31.

Tontonoz B, Hu E, Spiegelman BM. Stimulation of adipogenesis in fibroblasts by PPAR γ2, a lipid-activated transcription factor. *Cell* 1994; 79: 1147-1156.

Troiano RP, Flegal KM. Overweight children and adolescents: description, epidemiology and demographics. *Pediatrics* 1998; 101(3):497-504.

Turnbull BW. The empirical distribution function from arbitrarily grouped, censored, and truncated data. *Journal of the Royal Statistical Society Series B* 1976; 38:290-295.

UK Propsective Diabetes Study Group. Intensive blood glucose control with sulphonylureas or insulin compared with conventional treatment and risk of complications in patients with Type 2 diabetes (UKPDS 33). *Lancet* 1998; 352:837-853.

UK Prospective Diabetes (UKPDS) Group. Effect of intensive blood-glucose control with metformin on complications in overweight patients with type 2 diabetes (UKPDS 34). *Lancet* 1998; 352:854-865.

Unger RH, Orci L. Lipotoxic disease of nonadipose tisses in obesity. *Int J Obes Relat Metab Disord* 2000; 24:S28-32.

U.S. Department of Health and Human Services (DHHS) National Center for Health Statistics. *NHANES III Reference Manuals and Reports*, Hyattsville, MD, Centers for Disease Control and Prevention, 1996.

Uwaifo GI, Fallon EM, Chin J, Elberg J, Parikh SJ, Yanovski JA. Indices of insulin action, disposal, and secretion derived from fasting samples and clamps in normal glucose-tolerant black and white children. *Diabetes Care* 2002 25(11):2081-7.

Varni JW, Burwinkle TM, Jacobs JR, Gottschalk M, Kaufman F, Jones KL. The PedsQL in type 1 and type 2 diabetes: reliability and validity of the Pediatric Quality of Life inventory generic core scales and type 1 diabetes module. *Diabetes Care* 2003; 26(3):631-637.

Wei LJ. The accelerated failure time model: a useful alternative to the Cox regression model in survival analysis. *Statistics in Medicine* 1992;11:1871-1879.

Wei LJ, Lachin JM. Two-sample asymptotically distribution-free tests for incomplete multivariate observations. *J Amer Stat Assoc* 1984; 79:653-661.

West KM, Kalbfleisch JM. Influence of nutritional factors on prevalence of diabetes. *Diabetes* 1971; 20:99-108.

Weston AT, Petosa R, Pate RR. Validation of an instrument for measurement of physical activity in youth. *Med Sci Sports Exerc* 1997; 29(1):138-43.

Wing RR, Koeske R, Epstein LH, Nowalk MP, Gooding W, Becker D. Long-term effects of modest weight loss in type 2 diabetic patients. *Arch Intern Med* 1987; 147:1749-1753.

Wollen N, Bailey CJ. Inhibition of hepatic glyconeogenesis by metformin: synergism with insulin. *Biochem Pharmacol* 1988; 37:4353-4358.

Yokoyama H, Okudaira M, Otani T, Sato A, Miura J, Takaike H, Yamada H, Muto K, Uchigata Y, Ohashi Y, Iwamoto Y. Higher incidence of diabetic nephropathy in type 2 than in type 1 diabetes in early-onset diabetes in Japan. *Kidney Int* 2000; 58(1):302-11.

Yoshida Y, Hagura R, Hara Y, Sugasawa G, Akanuma Y. Risk factors for the development of diabetic retinopathy in Japanese type 2 diabetic patients. *Diabetes Res Clin Pract* 2001; 51(3):195-203.

Zavaroni I, Bonora E, Pagliara M, Dall'Aglio E, Luchetti L, Buonanno G, Bonati PA, Bergonzani M, Gnudi L, Passeri M, et al. Risk factors for coronary artery disease in healthy persons with hyperinsulinemia and normal glucose tolerance. *N Engl J Med* 1989; 320(11):702-6.

Zavaroni I, Mazza S, Luchetti L, Buonanno G, Bonati PA, Bergonzani M, Passeri M, Reaven GM. High plasma insulin and triglyceride concentrations and blood pressure in offspring of people with impaired glucose tolerance. *Diabet Med* 1990; 7(6):494-8.

Zieske AW, Malcom GT, Strong JP. Natural history and risk factors of artherosclerosis in children and youth: the PDAY study. *Pediatr Pathol Mol Med* 2002; 21(2): 213-237.
