# Supplementary material for: Growth Hormone Mediators and Glycemic Control in Youths With Type 2 Diabetes: A Secondary Analysis of a Randomized Clinical Trial
Source: JAMA Netw Open. 2024 Feb 29;7(2):e240447. doi: 10.1001/jamanetworkopen.2024.0447 (PMC10905312; doi:10.1001/jamanetworkopen.2024.0447)
Supplement: Supplement 2. — eFigure 1. CONSORT Study Flow Diagram of the TODAY Secondary Analysis eFigure 2. Time-to-Event Survival Analysis of the TODAY Substudy Cohort eTable 1. Anthropometric, Demographic, and Clinical Characteristics at Study Entry Based on Primary Outcome in TODAY eTable 2. Anthropometric, Demographic, and Clinical Characteristics Compared Between Subcohort With Only Baseline Samples and Subcohort With Available Samples at Baseline and 36 Months eTable 3. Glycemic Measures at 36-Month Study Timepoint Based on Primary Outcome in TODAY eTable 4. Glycemic Measures at 36-Month Study Timepoint Compared Between Subcohort With Only Baseline Samples and Subcohort With Available Samples at Baseline and 36 Months eFigure 3. Correlations Between GH Mediators [file jamanetwopen-e240447-s002.pdf]

Supplementary Online Content

Lu C, Wolfs D, El ghormli L, et al. Growth hormone mediators and glycemic control in youths with type 2 diabetes: a secondary analysis of a randomized clinical trial. *JAMA Netw Open.* 2024;7(3):e240447. doi:10.1001/jamanetworkopen.2024.0447

eFigure 1. CONSORT Study Flow Diagram of the TODAY Secondary Analysis
eFigure 2. Time-to-Event Survival Analysis of the TODAY Substudy Cohort
eTable 1. Anthropometric, Demographic, and Clinical Characteristics at Study Entry Based on Primary Outcome in TODAY
eTable 2. Anthropometric, Demographic, and Clinical Characteristics Compared Between Subcohort With Only Baseline Samples and Subcohort With Available Samples at Baseline and 36 Months
eTable 3. Glycemic Measures at 36-Month Study Timepoint Based on Primary Outcome in TODAY
eTable 4. Glycemic Measures at 36-Month Study Timepoint Compared Between Subcohort With Only Baseline Samples and Subcohort With Available Samples at Baseline and 36 Months
eFigure 3. Correlations Between GH Mediators

This supplementary material has been provided by the authors to give readers additional information about their work.

© 2024 Lu C et al. *JAMA Network Open.*

eFigure 1. CONSORT Study Flow Diagram of the TODAY Secondary Analysis

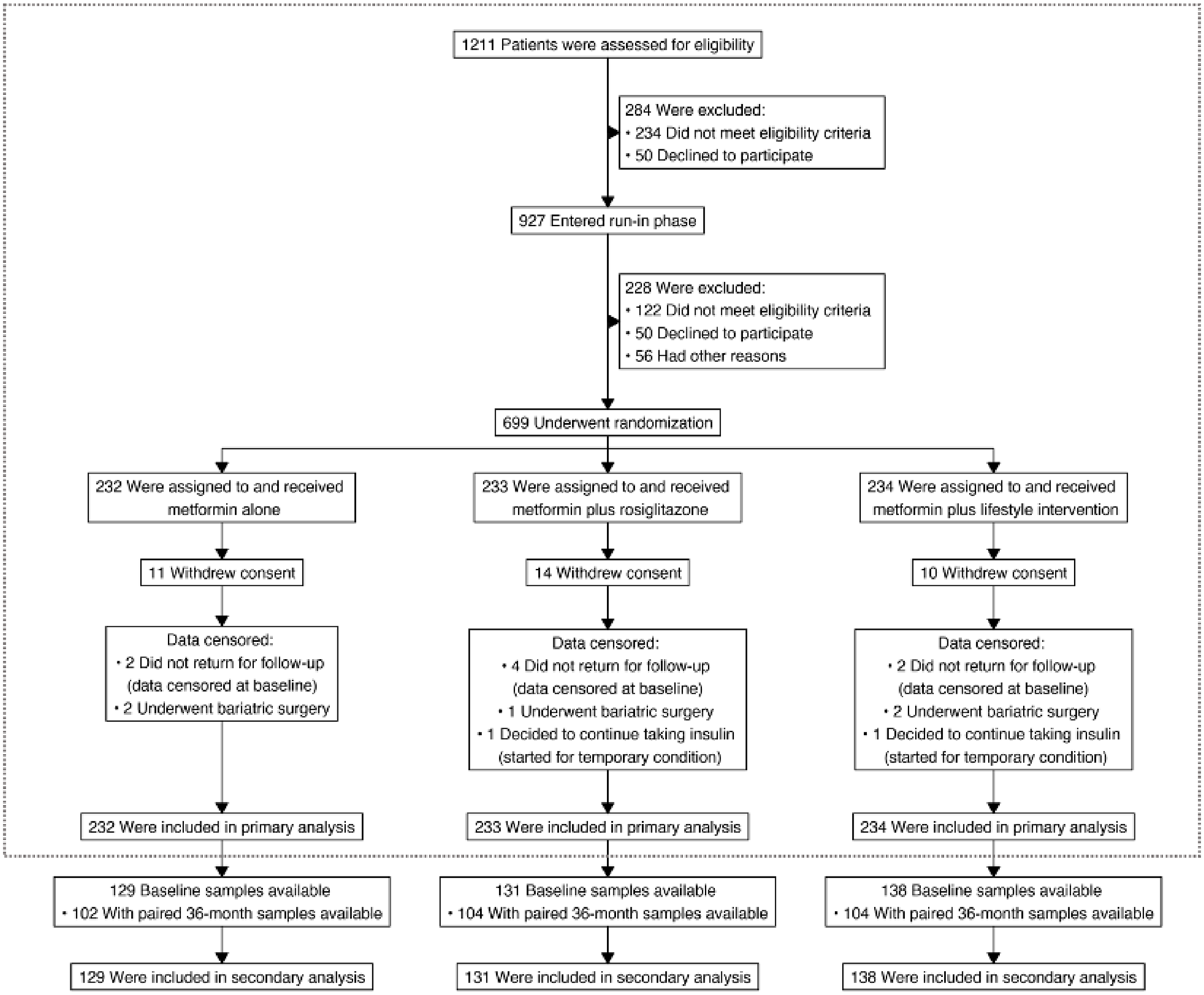

Enclosed in dashed lines is CONSORT diagram adapted from original publication from the TODAY Study Group; Bjornstad P, Drews KL, Caprio S, Gubitosi-Klug R, Nathan DM, Tesfaldet B, Tryggestad J, White NH, Zeitler P. Long-Term Complications in Youth-Onset Type 2 Diabetes. N Engl J Med. 2021 Jul 29;385(5):416-426. doi: 10.1056/NEJMoa2100165. PMID: 34320286; PMCID: PMC8697255.

eFigure 2. Time-to-Event Survival Analysis of the TODAY Substudy Cohort

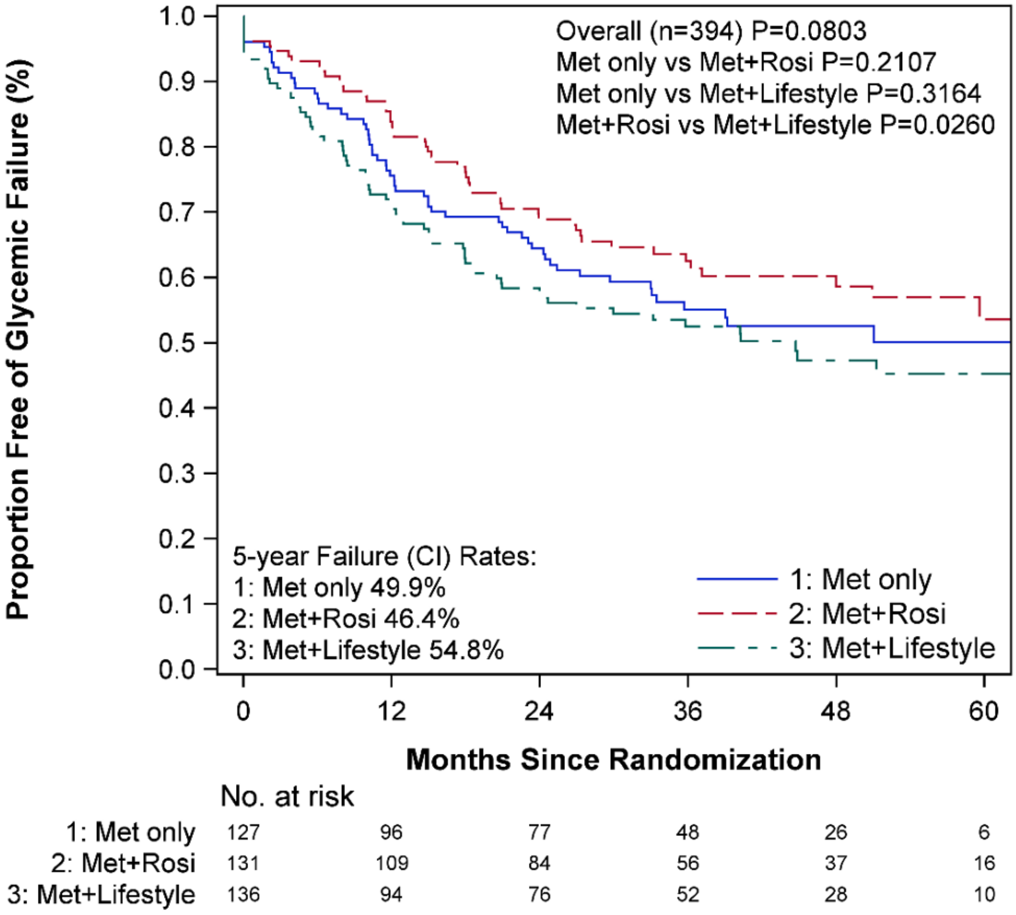

© 2024 Lu C et al. *JAMA Network Open.*

eTable 1. Anthropometric, Demographic, and Clinical Characteristics at Study Entry Based on Primary Outcome in TODAY

	**All (N=398)**	**Maintained glycemic control (N=216)**	**Loss of glycemic control (N=182)**	**P-value**
Female sex (%)	248 (62%)	141 (65%)	108 (59%)	0.26
Age, years	13.9 ± 2.0	13.8 ± 1.9	14.1 ± 2.0	
BMI, kg/m^2	34.7 ± 8.2	34.8 ± 8.6	34.7 ± 7.7	0.94
BMI z-score	2.2 ± 0.5	2.2 ± 0.5	2.2 ± 0.5	0.80
Weight percentile	97.0 ± 6.0	96.9 ± 6.5	97.1 ± 5.3	0.87
Height percentile	63.6 ± 30.6	64.2 ± 30.1	63.0 ± 31.1	0.71
**Tanner stage**				0.61
Tanner stage I-III	39 (10%)	23 (11%)	16 (9%)	
Tanner stage IV, V	353 (90%)	189 (89%)	164 (91%)	
**Race and ethnic group, %**				0.02
Non-Hispanic Black	134 (34%)	66 (31%)	68 (37%)	
Hispanic	166 (42%)	84 (39%)	82 (45%)	
Non-Hispanic White	84 (21%)	57 (26%)	27 (15%)	
Other	14 (3%)	9 (4%)	5 (3%)	
**Household income**				0.18
<$24,999	151 (43%)	82 (42%)	69 (44%)	
$25K-49,999	122 (34%)	62 (32%)	60 (38%)	
>$50K	81 (23%)	52 (26%)	29 (18%)	
**Highest household education**				0.33
Less than HS	114 (29%)	59 (28%)	55 (31%)	
HS, GED, business/technical school	89 (23%)	45 (21%)	44 (24%)	
Some college	122 (31%)	65 (31%)	57 (32%)	
College	67 (17%)	43 (20%)	24 (13%)	
**Insurance (% yes)**	369 (94%)	201 (95%)	168 (94%)	0.67
Medicare	225 (61%)	117 (58%)	108 (64%)	0.21
Private	154 (42%)	89 (44%)	65 (39%)	0.26
**Treatment assignment at start of clinical trial - %**				0.14
Metformin alone	129 (32%)	70 (32%)	59 (32%)	
Metformin plus rosiglitazone	131 (33%)	79 (37%)	52 (29%)	
Metformin plus lifestyle intervention	138 (35%)	67 (31%)	71 (39%)	
Time since diagnosis of type 2 diabetes, months	7.5 ± 5.7	7.0 ± 5.4	8.2 ± 6.0	0.04
**Baseline glycemic measures**				
HbA1c, %	6.1 ± 0.8	5.7 ± 0.6	6.4 ± 0.8	<0.001
Fasting glucose, mg/dL	111 ± 24	101 ± 16	122 ± 27	<0.001
2hr OGTT glucose, mg/dL	204 ± 63	181 ± 49	231 ± 66	<0.001
2hr OGTT C-peptide, ng/mL	11.0 ± 5.3	12.1 ± 5.7	9.6 ± 4.3	<0.001
1/fasting C-peptide, mL/ng	0.32 ± 0.15	0.32 ± 0.14	0.32 ± 0.17	0.65
C-peptide index, ng/mL per mg/dL	0.08 ± 0.07	0.10 ± 0.07	0.05 ± 0.05	<0.001
CODI, mL/uU x ng/mL per mg/dL	0.003 ± 0.004	0.004 ± 0.004	0.002 ± 0.004	<0.001
HMWA, ng/mL	2848 ± 1815	2919 ± 1780	2763 ± 1857	0.40

Continuous data are reported as mean ± SD, and categorical variables are reported as number (%).
Abbreviations: OGTT, oral glucose tolerance test; CODI, C-peptide oral disposition index; HMWA, high-molecular-weight adiponectin

© 2024 Lu C et al. *JAMA Network Open.*

eTable 2. Anthropometric, Demographic, and Clinical Characteristics Compared Between Subcohort With Only Baseline Samples and Subcohort With Available Samples at Baseline and 36 Months

	All (N=398)	Baseline only (N=88)	Paired samples (N=310)	P-value
Female sex (%)	248 (62%)	58 (66%)	190 (61%)	0.43
Age, years	13.9 ± 2.0	14.4 ± 1.9	13.8 ± 2.0	**0.008**
BMI, kg/m^2	34.7 ± 8.2	34.8 ± 7.9	34.7 ± 8.3	0.93
BMI z-score	2.2 ± 0.5	2.2 ± 0.4	2.2 ± 0.5	0.38
Weight percentile	97.0 ± 6.0	96.5 ± 5.6	97.2 ± 6.1	0.39
Height percentile	63.6 ± 30.6	57.1 ± 31.2	65.5 ± 30.3	**0.02**
**Tanner stage**				0.79
Tanner stage I-III	39 (10%)	8 (9%)	31 (10%)	
Tanner stage IV, V	353 (90%)	79 (91%)	274 (90%)	
**Race and ethnic group, %**				0.13
Non-Hispanic Black	134 (34%)	30 (34%)	137 (44%)	
Hispanic	166 (42%)	29 (33%)	104 (34%)	
Non-Hispanic White	84 (21%)	24 (27%)	60 (19%)	
Other	14 (3%)	5 (6%)	9 (3%)	
**Household income**				0.81
<$24,999	151 (43%)	31 (40%)	120 (43%)	
$25K-49,999	122 (34%)	29 (37%)	93 (34%)	
>$50K	81 (23%)	18 (23%)	63 (23%)	
**Highest household education**				0.27
Less than HS	114 (29%)	34 (39%)	94 (31%)	
HS, GED, business/technical school	89 (23%)	20 (23%)	88 (29%)	
Some college	122 (31%)	18 (21%)	71 (23%)	
College	67 (17%)	15 (17%)	52 (17%)	
**Insurance (% yes)**	369 (94%)	79 (91%)	290 (95%)	0.10
Medicare	225 (61%)	42 (48%)	183 (63%)	0.11
Private	154 (42%)	40 (51%)	114 (61%)	0.07
**Treatment assignment at start of clinical trial - %**				0.67
Metformin alone	129 (32%)	27 (30.5%)	102 (33%)	
Metformin plus rosiglitazone	131 (33%)	27 (30.5%)	104 (33.5%)	
Metformin plus lifestyle intervention	138 (35%)	34 (39%)	104 (33.5%)	
Time since diagnosis of type 2 diabetes, months	7.5 ± 5.7	6.9 ± 5.2	7.7 ± 5.8	0.21
**Baseline glycemic measures**				
HbA1c, %	6.1 ± 0.8	6.1 ± 0.7	6.0 ± 0.8	0.71
Fasting glucose, mg/dL	111 ± 24	112 ± 25	110 ± 24	0.58
2hr OGTT glucose, mg/dL	204 ± 63	203 ± 65	204 ± 62	0.90
2hr OGTT C-peptide, ng/mL	11.0 ± 5.3	10.8 ± 5.5	11.0 ± 5.2	0.75
1/fasting C-peptide, mL/ng	0.32 ± 0.15	0.32 ± 0.16	0.32 ± 0.15	0.60
C-peptide index, ng/mL per mg/dL	0.08 ± 0.07	0.07 ± 0.07	0.08 ± 0.07	0.52
CODI, mL/uU x ng/mL per mg/dL	0.003 ± 0.004	0.003 ± 0.002	0.004 ± 0.004	0.24
HMWA, ng/mL	2848 ± 1815	2944 ± 1738	2820 ± 1838	0.58

Continuous data are reported as mean ± SD, and categorical variables are reported as number (%).
Abbreviations: OGTT, oral glucose tolerance test; CODI, C-peptide oral disposition index; HMWA, high-molecular-weight adiponectin

© 2024 Lu C et al. *JAMA Network Open.*

eTable 3. Glycemic Measures at 36-Month Study Timepoint Based on Primary Outcome in TODAY

	All (N=398)	Maintained glycemic control (N=216)	Loss of glycemic control (N=182)	P-value
**36 months**				
HbA1c, %	7.7 ± 2.6	6.1 ± 1.2	9.6 ± 2.4	<0.001
Fasting glucose, mg/dL	156 ± 75	116 ± 34	202 ± 82	<0.001
2hr OGTT glucose, mg/dL	244 ± 99	196 ± 76	317 ± 85	<0.001
2hr OGTT C-peptide, ng/mL	8.1 ± 5.3	10.1 ± 5.4	5.0 ± 3.3	<0.001
1/fasting C-peptide, mL/ng	0.56 ± 1.4	0.34 ± 0.2	0.81 ± 2.0	<0.001
C-peptide index, ng/mL per mg/dL	0.05 ± 0.05	0.07 ± 0.06	0.02 ± 0.03	<0.001
CODI, mL/uU x ng/mL per mg/dL	0.002 ± 0.003	0.003 ± 0.003	0.0007 ± 0.001	<0.001
HMWA, ng/mL	3566 ± 3436	3991 ± 3873	3058 ± 2765	0.06

Abbreviations: OGTT, oral glucose tolerance test; CODI, C-peptide oral disposition index; HMWA, high-molecular-weight adiponectin

© 2024 Lu C et al. *JAMA Network Open.*

eTable 4. Glycemic Measures at 36-Month Study Timepoint Compared Between Subcohort With Only Baseline Samples and Subcohort With Available Samples at Baseline and 36 Months

	**All (N=398)**	**Baseline only (N=88)**	**Paired samples control (N=310)**	**P-value**
**36 months**				
HbA1c, %	7.7 ± 2.6	8.7 ± 3.1	7.7 ± 2.5	0.07
Fasting glucose, mg/dL	156 ± 75	170 ± 89	155 ± 74	0.46
2hr OGTT glucose, mg/dL	244 ± 99	268 ± 119	243 ± 98	0.41
2hr OGTT C-peptide, ng/mL	8.1 ± 5.3	8.7 ± 8.9	8.0 ± 5.1	0.70
1/fasting C-peptide, mL/ng	0.56 ± 1.4	0.49 ± 0.30	0.56 ± 1.4	0.87
C-peptide index, ng/mL per mg/dL	0.05 ± 0.05	0.05 ± 0.09	0.05 ± 0.05	0.97
CODI, mL/uU x ng/mL per mg/dL	0.002 ± 0.003	0.002 ± 0.002	0.002 ± 0.003	0.42
HMWA, ng/mL	3566 ± 3436	3450 ± 1593	3568 ± 3467	0.95

Abbreviations: OGTT, oral glucose tolerance test; CODI, C-peptide oral disposition index; HMWA, high-molecular-weight adiponectin

© 2024 Lu C et al. *JAMA Network Open.*

eFigure 3. Correlations Between GH Mediators

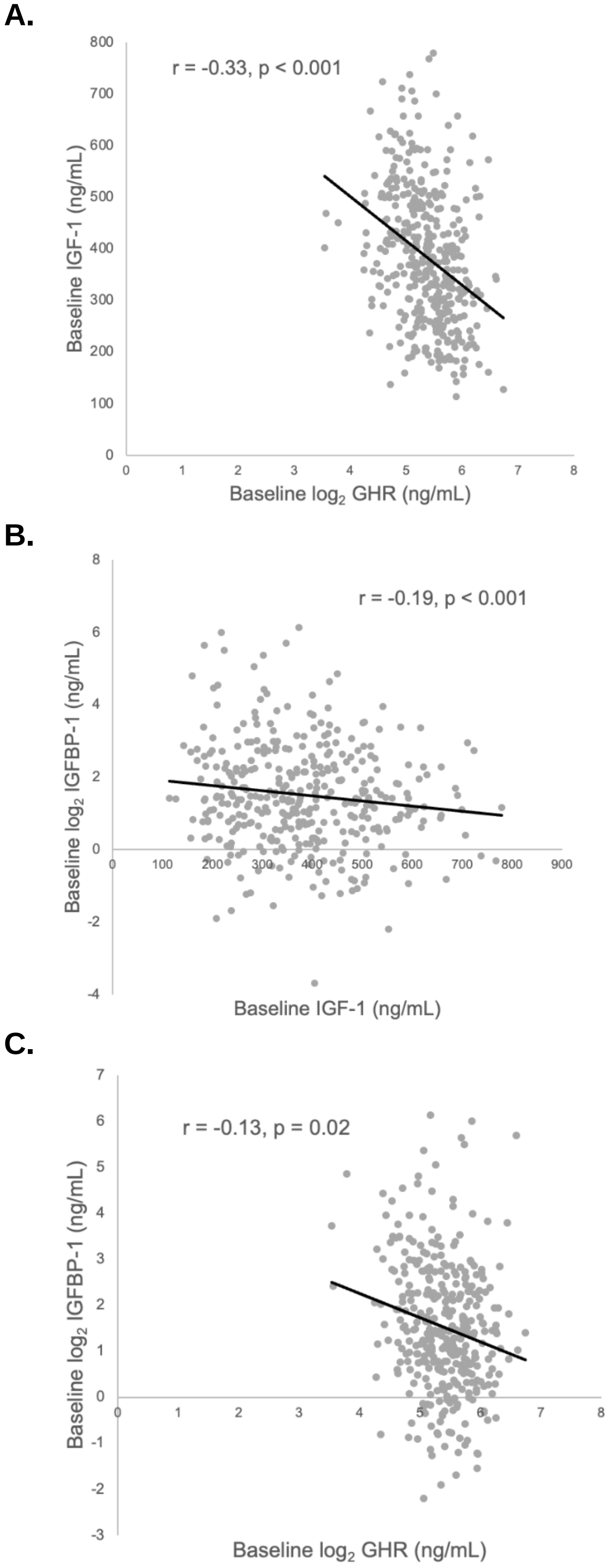

Baseline plasma concentrations of IGF-1 (N=310), GHR (N=310), and IGFBP-1 (N=286) were correlated with one another using Pearson correlation. R is Pearson's correlation coefficient with corresponding p value.

© 2024 Lu C et al. *JAMA Network Open.*
