# Supplementary material for: Growth Hormone Mediators and Glycemic Control in Youths With Type 2 Diabetes: A Secondary Analysis of a Randomized Clinical Trial
Source: JAMA Netw Open. 2024 Feb 29;7(2):e240447. doi: 10.1001/jamanetworkopen.2024.0447 (PMC10905312; doi:10.1001/jamanetworkopen.2024.0447)
Supplement: Supplement 3. — Data Sharing Statement [file jamanetwopen-e240447-s003.pdf]

Data Sharing Statement

Lu. Growth Hormone Mediators and Glycemic Control in Youths With Type 2 Diabetes. *JAMA Netw Open*. Published March 01, 2024. doi:10.1001/jamanetworkopen.2024.0447

Data
Data available: Yes
Data types: Deidentified participant data
How to access data: Deidentified participant data are available in the NIDDK Central Repository, as linked above.
When available: beginning date: 04-01-2023

Supporting Documents
Document types: None

Additional Information
Who can access the data: Anyone requesting the data
Types of analyses: For any purpose
Mechanisms of data availability: Data are publicly available
